# Phagocytosing differentiated cell-fragments is a novel mechanism for controlling somatic stem cell differentiation within a short time frame

**DOI:** 10.1007/s00018-022-04555-0

**Published:** 2022-10-06

**Authors:** Shohei Wakao, Yo Oguma, Yoshihiro Kushida, Yasumasa Kuroda, Kazuki Tatsumi, Mari Dezawa

**Affiliations:** 1 Department of Stem Cell Biology and Histology, Tohoku University Graduate School of Medicine, 2-1, Seiryo-Machi, Aoba-Ku, Sendai, 980-8575 Japan; 2 Regenerative Medicine Division, Analytical Research Department, Technology Development Unit, Life Science Institute, Inc., Tokyo, Japan

**Keywords:** Single-cell RNA sequencing, Apoptotic cells, Somatic stem cells, Muse cells, Differentiation, Phagosomal release

## Abstract

Stem cells undergo cytokine-driven differentiation, but this process often takes longer than several weeks to complete. A novel mechanism for somatic stem cell differentiation via phagocytosing ‘model cells’ (apoptotic differentiated cells) was found to require only a short time frame. Pluripotent-like Muse cells, multipotent mesenchymal stem cells (MSCs), and neural stem cells (NSCs) phagocytosed apoptotic differentiated cells via different phagocytic receptor subsets than macrophages. The phagocytosed-differentiated cell-derived contents (e.g., transcription factors) were quickly released into the cytoplasm, translocated into the nucleus, and bound to promoter regions of the stem cell genomes. Within 24 ~ 36 h, the cells expressed lineage-specific markers corresponding to the phagocytosed-differentiated cells, both in vitro and in vivo. At 1 week, the gene expression profiles were similar to those of the authentic differentiated cells and expressed functional markers. Differentiation was limited to the inherent potential of each cell line: triploblastic-, adipogenic-/chondrogenic-, and neural-lineages in Muse cells, MSCs, and NSCs, respectively. Disruption of phagocytosis, either by phagocytic receptor inhibition via small interfering RNA or annexin V treatment, impeded differentiation in vitro and in vivo. Together, our findings uncovered a simple mechanism by which differentiation-directing factors are directly transferred to somatic stem cells by phagocytosing apoptotic differentiated cells to trigger their rapid differentiation into the target cell lineage.

**Supplementary Information:**

The online version contains supplementary material available at 10.1007/s00018-022-04555-0.

## Introduction

Stem cells are generally considered to initiate differentiation in response to factors delivered from outside the cell, such as cytokines, morphogens, cell adhesion molecules, and the extracellular matrix. These factors bind to receptors on the stem cells to activate specific signal transduction pathways and induce the expression of genes that characterize the differentiated cells [1]. Therefore, in vitro differentiation of stem cells is usually based on a series of cytokine treatments and/or gene introduction that takes longer than several weeks [1].

Mesenchymal stem cells (MSCs), a somatic stem cell type collectable as adherent cells from mesenchymal tissues such as the bone marrow and adipose tissue, exhibit multipotency as they can efficiently differentiate into adipogenic-, chondrogenic-, and osteogenic-lineage cells by induction with specific sets of cytokines, but scarcely differentiate into other mesodermal-lineage cells or into ectodermal/endodermal-lineage cells [2, 3]. Neural stem cells (NSCs), locating in the ventricular–subventricular zone and dentate gyrus in the mammalian adult brain, are capable of generating neurons and glial cells, both in vivo and in vitro [4].

Multilineage-differentiating stress enduring (Muse) cells comprise another somatic stem cell type identified as pluripotent surface marker stage-specific embryonic antigen-3 (SSEA-3)-positive that distributes in the bone marrow, peripheral blood, and organ connective tissue as cells; Muse cells are pluripotent-like as they express pluripotent markers, including POU class 5 homeobox 1 (OCT3/4), Nanog homeobox (NANOG), and SRY-box transcription factor 2 (SOX2), and are capable of differentiating into triploblastic-lineages and self-renewing at the single-cell level [5–7]. In vitro, Muse cells efficiently differentiate into triploblastic lineage cells, including melanocyte-, cardiac-, neural-, and hepatic-lineage cells following induction by specific cytokines [8–10]. In vivo, Muse cells also differentiate into various cell types, but they exhibit a unique differentiation process: after selectively migrating to the site of damage by sensing the general damage signal sphingosine-1-phosphate (S1P) via S1P receptor 2 located on the surface of the Muse cells, they differentiate into multiple tissue-constituent cells in the homed tissue without prior induction; i.e., they differentiate into cardiomyocytes and endothelial cells in post-infarct heart; neurons and glial cells in post-infarct brain; and hepatocytes and cholangiocytes in damaged liver [11–13]. The in vivo differentiation proceeds rapidly; for example, cytokine-induced differentiation into melanocytes and cardiomyocytes in vitro requires 6 weeks and 4 weeks, respectively [9, 10], while in vivo, Muse cells express progenitor markers such as *NEUROD* and achaete-scute homolog 1 (*MASH1*) within 3 days and maturity markers such as microtubule-associated protein 2 (*MAP2*) and neuronal nuclei (*NEUN*) within 7 days after homing into the post-infarct region in a rat stroke model [12]. Therefore, the mechanisms of differentiation of Muse cells in vivo are suggested to differ from those of cytokine-induced differentiation in vitro.

As represented by macrophages, professional phagocytes are indispensable for tissue homeostasis by removing dying cells and releasing anti-inflammatory mediators, as well as for the innate immune response, protecting the body from invading microbes [14]. Recent studies, however, demonstrated that normal healthy somatic cells, such as mammary and bronchial epithelial cells, myocardiocytes, and fibroblasts, as well as somatic stem cells such as MSCs and NSCs, are capable of ‘non-professional’ phagocytosis to remove apoptotic cells/bodies, thereby contributing to tissue homeostasis and avoiding inflammation [15–21]. We also previously observed phagocytosis-like activity in Muse cells (unpublished data).

In the present study, we explored whether phagocytosis could be utilized to control the differentiation of various types of somatic stem cells within a short time frame. Muse cells, MSCs, and NSCs phagocytosed apoptotic differentiated cells as ‘model cells,’ which activated lineage-specific differentiation into the same lineage as the ‘model cells’ but not into other lineages. The lineage-specific marker was expressed within 24 ~ 36 h after phagocytosis, while the differentiation range was confined to the inherent differentiation potential of each stem cell type; Muse cells differentiated into triploblastic-lineages; MSCs differentiated into adipogenic-/chondrogenic-lineages, but not into other mesodermal cells or ectodermal-/endodermal-lineages; and NSCs differentiated into neuronal and glial cells. The gene expression profiles of the stem cells 1 week after phagocytosis were similar to those of the authentic differentiated cells and clearly distinct from those of naïve stem cells or cells of other lineages. One potential mechanism of the phagocytosis-induced differentiation is the rapid discharge of the contents of the engulfed phagosome into the cytoplasm after the docking of 2 cell membranes wrapping phagosomes, which enables the stem cells to directly utilize machineries such as transcription factors originally functioning in the phagocytosed apoptotic differentiated cells. Disrupting phagocytosis impeded the lineage-specific differentiation both in vitro and in vivo.

While the main function of phagocytosis has long been considered to be the clearance of tissue debris and microbes, the findings of the present study demonstrated another biologically significant function of non-professional phagocytosis. Furthermore, this mechanism can be utilized as a simple quick method for controlling the differentiation of some somatic stem cells with fewer errors.

## Results

Muse cells are collectable from many tissue sources and are also obtainable as SSEA-3(+) cells as several percent of MSCs [13, 22–24]. We collected human (h)-Muse cells from h-bone marrow MSCs as described previously [6, 25]. SSEA-3(−) MSCs, i.e., cells other than Muse cells among MSCs, exhibit a repertoire of surface marker and gene expression patterns similar to those of conventional MSCs, with pluripotency marker expression levels under the limits of detection or at very low levels as well as a differentiation ability comparable to that of conventional MSCs and differentiate into adipogenic-, chondrogenic-, and osteogenic-lineage cells by the same protocol as used for general MSC populations, but not into other mesodermal-lineage cells or ectodermal-/endodermal-lineage cells [8, 25, 26]. Therefore, in this study, we refer to SSEA-3(−)-h-bone marrow MSCs as ‘h-MSCs.’ NSCs were collected from green fluorescent protein (GFP)-rat E14.5 embryonic brain.

To analyze lineage-specific marker expression in h-Muse cells and h-MSCs, differentiated cells prepared from mouse (m-) and rat (r-) were treated with either etoposide, antimycin, or rotenone to generate m- and r-apoptotic cell fragments. To analyze the neural differentiation of r-NSCs, h-neural cells induced from the h-neural progenitor cell line ReNcell were used as ‘differentiated cells’ and treated with antimycin and rotenone to generate h-apoptotic neural cell fragments for incubation with r-NSCs.

### Human Muse cells incubated with apoptotic-cell fragments committed to the same lineage as that of the apoptotic cells

**Fig. 1:**
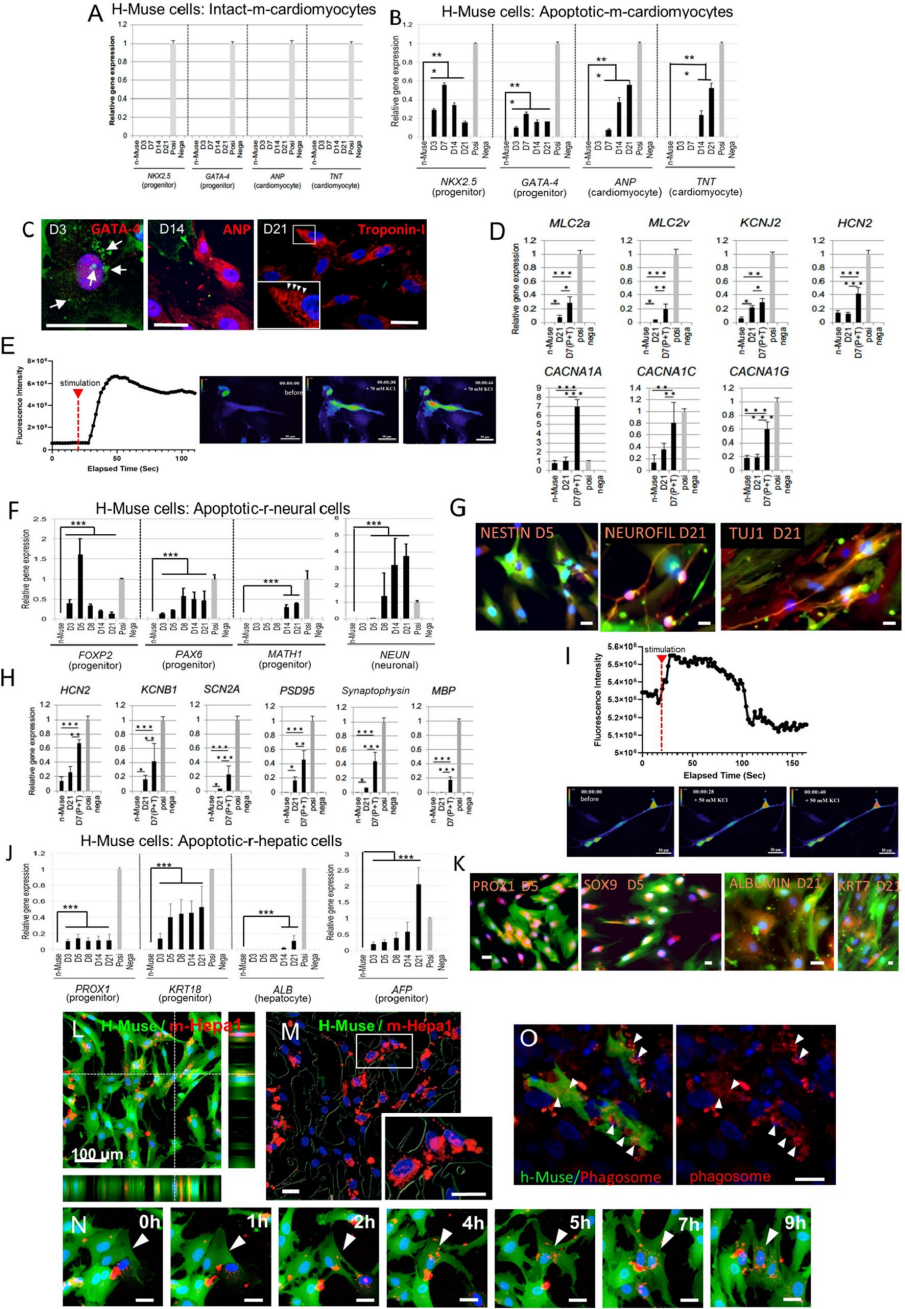
Lineage-specific differentiation and phagocytic activity in h-Muse cells. (**A**, **B**, **D**, **F**, **H**, **J**) (**A**) qPCR of human-cardiac markers in naïve-Muse cells (n-Muse) and h-Muse cells after co-culture with intact m-cardiomyocytes from D3 to D21 (mean ± SEM). qPCR of human-cardiac (**B**, **D**), -neural (**F**, **H**), and -hepatic (**J**) markers in naïve h-Muse cells (n-Muse) and h-Muse cells after incubating with apoptotic fragments derived from m-cardiomyocyte, r-neural cells, and r-hepatic cells from D3 to D21 (mean ± SEM). In **D** and **H**, D7 (P + T) indicates h-Muse cells incubated with apoptotic-cell fragments for 3 days and then co-cultured with a damaged tissue slice for 7 days (mean ± SEM). Confirmed species-specific primers were used in qPCR. Apoptotic cell fragments were used as a negative control (Nega). For the positive control, human fetal heart total RNA (**A**, **B**), h-adult heart (**D**), and h-fetus whole total RNA (**F**, **H**, **J**) were used (Posi). *p < 0.05, **p < 0.01, ***p < 0.001. **C**, **G**, **K** Immunocytochemistry in h-Muse cells after incubating with apoptotic fragments. **C** Non-labeled h-Muse cells were incubated with apoptotic GFP-m-cardiomyocyte fragments. Arrows: GFP-positive fragments/particles in the cytoplasm. Inset arrowheads: striated-like pattern of troponin-I in h-Muse cells. **G**, **K** h-Muse cells were transduced with GFP-lentivirus for identification. Apoptotic fragments were from non-labeled cells. Low magnification images of (**G**) are shown in Fig. S2B. **E**, **I** Intracellular calcium dynamics in green fluorescent protein (GFP)-based Ca calmodulin probe (GCaMP)-h-Muse cells after biochemical depolarization with 70 mM (**E**) and 50 mM (**I**) KCl, respectively (added at 20 s) (see Movies 1). The change in the fluorescence intensity over time was demonstrated. **L**–**N** Laser confocal microscopy of GFP-h-Muse cells incubated with mCherry-m-Hepa1 DDCs. **L** Live imaging at 20 h (see Movie 2) shows Z-series 3D construction. **M** Analysis of (**L**) by Bitplane Imaris software revealed phagocytosed fragments in h-Muse cells. Inset: magnification of the box area. **M** Transition of large mCherry-fragments to smaller particles after phagocytosis (see Movie 2). **N** GFP-h-Muse cells in the post-infarct rat heart tissue at D5 contained LAMP-1( +) phagosomes (red, arrowheads). Bars: **C**, **G**, **K** = 25 μm; **E**, **I**, **M**–**O** = 50 μm; **L** = 100 μm

Human-specific cardiac markers h-NK-2 transcription factor related, locus 5 (*NKX2.5*), h-GATA binding protein 4 (*GATA-4*), h-atrial natriuretic peptide (*ANP*), and h-troponin-T (*TNT*), under the detection limits in naive h-Muse cells, remained undetectable in h-Muse cells after co-culture with intact primary cultured neonatal m-cardiomyocytes at a cell number ratio of 1:2 from day (D) 3 to D21 in quantitative polymerase chain reaction (qPCR) (Fig. 1A).

Floating dead cell fragments were collected from apoptotic m-cardiomyocytes by centrifugation. We confirmed for up to 7 days that these fragments contained no living cells (Supplementary Information [SI] 1). When h-Muse cells were incubated for 3 days with fragments derived from apoptotic m-cardiomyocytes in the medium and then cultured further after washing out the fragments, they newly expressed h*-NKX2.5* and h*-GATA-*4 at D3 after starting the incubation, h*-ANP* at D7, and h*-TNT* at D14 in qPCR (all with *p* < 0.001) (Fig. 1B). Immunocytochemistry also showed the expression of GATA-4 (D3), ANP (D14), and TNT (D21) in h-Muse cells (Fig. 1C, positive and negative controls for immunocytochemistry are all shown in S2A-C). The positive ratio for each marker is shown in SI3 (Fig. S3I). These findings indicate that cardiac-marker expression in h-Muse cells was activated by incubation with apoptotic m-cardiac cell fragments. On the other hand, other lineage markers, such as neural (h-forkhead box protein P2 [*FOXP2*], h-paired box 6 [*PAX6*], protein atonal homolog 1 [*MATH1*], and h-*NEUN)* and hepatic (h-prospero homeobox protein-1 [*PROX1*], h-Cytokeratin 18 [*KRT18*], h-albumin [*ALB*]*,* and h-alpha fetoprotein [*AFP*]) markers, were consistently under the qPCR detection limit (data not shown). The same tendency was observed when apoptotic m-cardiac muscle cell line (m-HL-1) fragments were used; expression of h*-NKX2,5*, h*-GATA-4*, h*-ANP*, and h*-TNT*, but not of neural (h-*FOXP2*, h-*PAX6*, h-*MATH1*, and h-*NEUN)* or hepatic (h-*PROX1*, h-*KRT18*, h-*ALB,* and h-*AFP*) markers, was detected (Fig. S4).

At D21, expression of cardiac function markers, such as myosin light chain 2 (*MLC2a* and *MLC2v*) and potassium channel (potassium inwardly rectifying channel subfamily J member 2 [*KCNJ2*]) [27], was upregulated (*p* < 0.05), while expression of potassium/sodium hyperpolarization-activated cyclic nucleotide-gated ion channel 2 (*HCN2*) and Ca channels (calcium voltage-gated channel subunit alpha 1 A [*CACNA1A*], calcium voltage-gated channel subunit alpha 1 C [*CACNA1C*], and calcium voltage-gated channel subunit alpha 1 G [*CACNA1G*]) [27] remained at levels similar to those in naive Muse cells in qPCR (Fig. 1D). We then investigated whether or not h-Muse cells incubated with apoptotic m-cardiomyocyte fragments for the first 3 days and then co-cultured with damaged m-cardiac tissue slices in the Boyden chamber called (D7 [P + T]) exhibited increased functional marker gene expression levels. Expression of all these markers was upregulated at D7 (P + T) compared with D21 (*MLC2a* and *KCNJ2*: *p* < 0.05, *MLC2v* and *CACNA1C*: *p* < 0.01, *HCN2*, *CACNA1A*, and *CACNA1G*: *p* < 0.001; Fig. 1D). In addition, the calcium-influx response to biochemical depolarization was enhanced (Fig. 1E, Movie 1).

Incubation of h-Muse cells with apoptotic neural cell fragments derived from primary culture of fetal r-hippocampus (r-neural cells) newly induced the expression of neural cell markers h*-FOXP2* and h*-PAX6* at D3, and h*-MATH1* at D14 in qPCR (all with *p* < 0.001). Human *NEUN* levels reached a maximum at D21 (*p* < 0.001) (Fig. 1F). Other lineage markers, such as cardiac (h*-NKX2,5*, h*-GATA-4*, h*-ANP*, and h*-TNT*) and hepatic (h-*PROX1*, h-*KRT18*, h-*ALB,* and h-*AFP*) markers, were consistently under the detection limits (data not shown). NESTIN (D5), NEUROFILAMENT (D21), and TUJ1 (D21) were detected by immunocytochemistry (Fig. 1G, S I2, Fig. S3I).

With regard to functional neural markers, *HCN2,* expressed at a low level in naïve h-Muse cells, was slightly but nonsignificantly upregulated at D21, and other markers, such as potassium voltage-gated channel (potassium voltage-gated channel subfamily B member 1 [*KCNB1*]), sodium channel (sodium voltage-gated channel alpha subunit 2 [*SCN2A*]), postsynaptic density protein 95 (*PSD95*), and *synaptophysin,* originally undetectable in naive h-Muse cells, were newly expressed at D21 (all with *p* < 0.05) in qPCR (Fig. 1H) [28, 29]. When h-Muse cells incubated with apoptotic-r-neural cell fragments for the first 3 days were then co-cultured with a damaged r-brain tissue slice for 7 days (D7 [P + T]), the levels of all the above markers were significantly upregulated compared with those at D21 (*HCN2*, *KCNB1* and *PSD95*; *p* < 0.01, *SCN2A* and *synaptophysin*; *p* < 0.001), and myelin basic protein (*MBP*), undetectable in both naïve h-Muse cells and at D21, became positive in qPCR (Fig. 1H), and a calcium-influx response to biochemical depolarization was promoted (Fig. 1I).

Apoptotic fragments of hepatic cells derived from primary culture of rat fetal liver (r-hepatic cells) newly induced expression of the endoderm markers *h-PROX1,* h- *KRT18*, and h-*AFP* at D3; and h-*ALB* at D14 in qPCR (*p* < 0.001) (Fig. 1J). Other lineage markers, such as cardiac (h*-NKX2,5*, h*-GATA-4*, h*-ANP*, and h*-TNT*) and neural (h-*FOXP2*, h-*PAX6*, h-*MATH1*, and h-*NEUN)* markers, were consistently under the detection limits (data not shown). Immunohistochemistry revealed the expression of PROX1 (D5), SRY-box transcription factor 9 (SOX9) (D5), ALB (D21), and Cytokeratin 7 (KRT7) (D21) in h-Muse cells (Fig. 1K, SI-2, Fig. S3I). Lineage-specific marker expression in h-Muse cells incubated with rat renal cells, keratinocytes, intestinal cells, and alveolar cells is shown in SI3.

Fusion of apoptotic cell fragments and h-Muse cells did not appear to be a major mechanism of lineage-specific marker expression in h-Muse cells (SI5-Fig. S5). Conditioned medium and cell extracts obtained from an h-Muse cell: apoptotic cell ratio of 1:100 or 200 μg/ml extracellular vesicles from intact and apoptotic cells did not induce differentiation marker expression in h-Muse cells (SI6-Fig. S6).

In the following experiments, cell fragments from m- and r-apoptotic differentiated cells are referred to as “differentiation-directing cells” (DDCs).

### Phagocytic activity of h-Muse cells

We next examined the reaction of h-Muse cells after incubation with DDCs. Live cell images obtained using laser confocal microscopy revealed that GFP-labeled h-Muse cells phagocytosed DDCs derived from the mCherry-introduced m-hepatic cell line Hepa1-6 (m-Hepa1) (Fig. 1L, Movie 2). Only the DDCs taken up into the GFP-h-Muse cells were analyzed and visualized using Bitplane Imaris software (Fig. 1M and Movie 2). When cells containing even 1 mCherry(+) particle were counted as positive, 86.9 ± 4.5% and 91.0 ± 3.2% of GFP-h-Muse cells had phagocytosed DDCs at 5 h and 20 h, respectively. In many cases, h-Muse cells initially phagocytosed larger DDCs of several micrometers, and then, the DDCs were dispersed into smaller particles several hundred nanometers in size within the cytoplasm (Fig. 1N, Movie 2).

To examine whether h-Muse cells also exhibit phagocytic activity in vivo, GFP-h-Muse cells were intravenously administered to a rat acute myocardial infarction model according to the previously reported method [13]. At D5, the GFP-h-Muse cells that homed to the infarcted heart area contained lysosome-associated membrane protein-1 (LAMP-1) -positive phagosomes in their cytoplasm, suggesting that h-Muse cells also have phagocytic activity in vivo (Fig. 1O).

The expression profile of macrophage-related markers in h-Muse cells was somewhat similar to that of macrophages in qPCR [30, 31], and some receptors were upregulated by incubation with m-cardiomyocyte DDCs. Expression of C–C motif chemokine ligand 2 (*CCL2*), cluster of differentiation 80 (*CD80*), interleukin-10 (*IL10*), interleukin-1 receptor 1 (*IL1R1*), and toll-like receptor 2 (*TLR2*) was upregulated more than tenfold at D1 or D2 compared with naïve h-Muse cells. In contrast, expression of cluster of differentiation 163 (*CD163*)*,* major histocompatibility complex, class II, DQ alpha 1 *(HLA-DQA1),* major histocompatibility complex, class II, DQ beta 1 *(HLA-DQB1),* interferon gamma *(IFNG)*, and toll like receptor 8 *(TLR8*) was consistently under the detection limits (SI 7-Fig. S7).

### Phagocytic activity and adipocyte- and chondrocyte-marker expression in h-MSCs

**Fig. 2:**
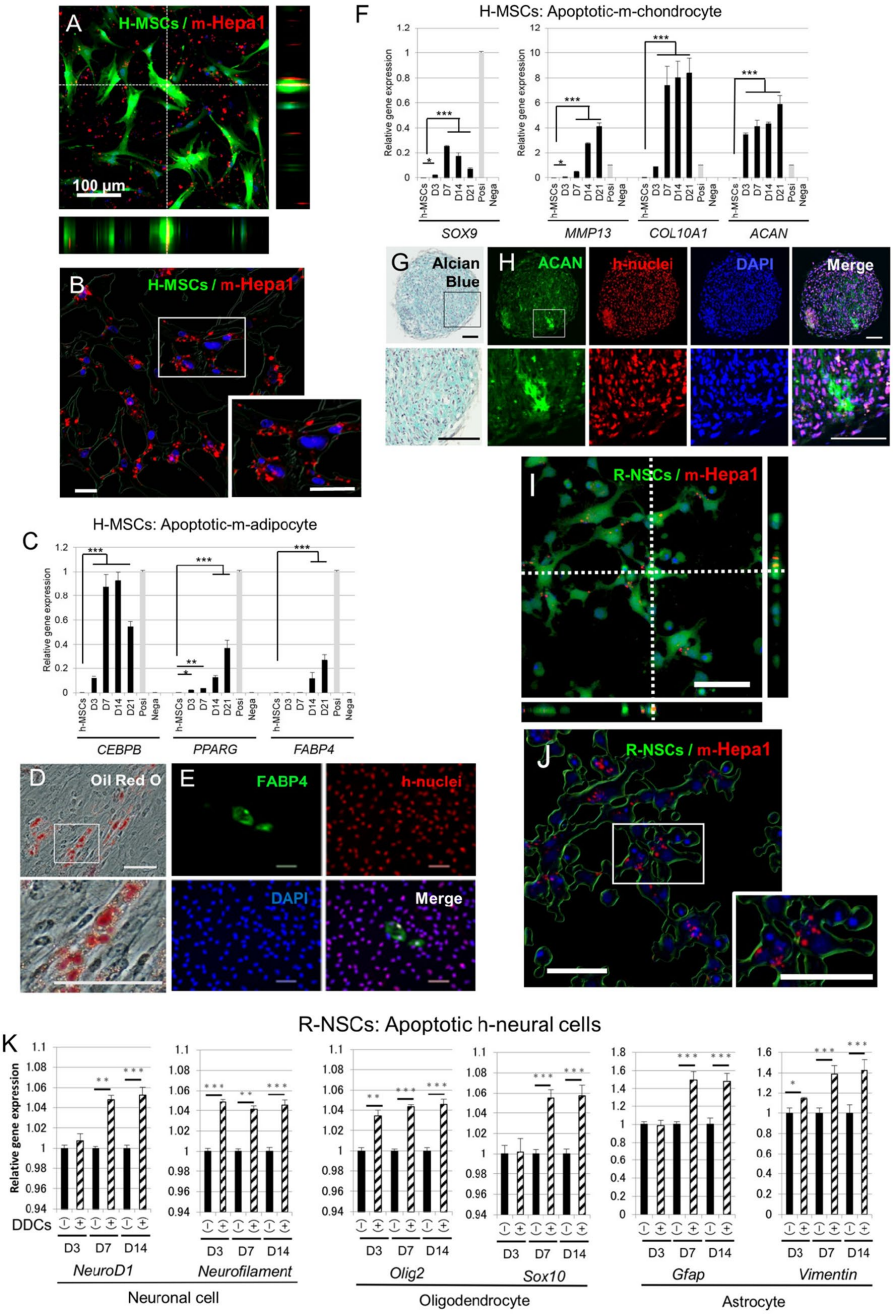
Phagocytic activity and lineage-specific differentiation of h-MSCs and r-NSCs. **A**, **B** Laser confocal microscopy of GFP-h-MSCs incubated with mCherry-m-Hepa1 DDC (**A**) at 20 h was used for Z-series 3D reconstruction. **B** Analysis of (**A**) by Bitplane Imaris software revealed phagocytosed fragments in h-MSCs. Inset is the high magnification of the box area. **C**, **F** qPCR of h-adipocyte (**C**) and -chondrocyte (**F**) markers in naïve h-Muse cells (n-Muse) and h-MSCs after incubating with m-adipocyte- (**C**) and m-chondrocyte-DDCs (**F**) from D3 to D21 (mean ± SEM). Confirmed species-specific primers were used in qPCR. Apoptotic cell fragments were used as a negative control (Nega). For the positive control, total RNA obtained from adipocytes and chondrocytes induced from human MSCs were used (Posi). **D**, **E**, **G**, **H** Histological assessment. **D**, **E** h-MSCs incubated with m-adipocyte DDCs contained lipid droplets in the cytoplasm stained by Oil Red at D21 (**D**). FABP4 was confirmed to be expressed in h-nuclei-positive h-MSCs at D21 (**E**). **G**, **H** h-MSCs incubated with m-chondrocyte DDCs and cultured in suspension exhibited positivity for Alcian blue at D21 (**G**). ACAN was confirmed to be expressed in h-nuclei-positive h-MSCs at D21 (**H**). **I** Laser confocal microscopy of GFP-r-NSCs incubated with mCherry-m-Hepa1 DDC (**A**) at 20 h. **J** Analysis of (**I**) by Bitplane Imaris software. **K** qPCR (mean ± SEM) of rat-neural markers in r-NSCs with [DDC( +)] or without [DDCs (−)] incubation with h-neural cell DDCs from D3 to D14. Positivity of each marker in adult rat brain total RNA and negativity in h-neural cells were confirmed in each primer. Bars: **A** = 100 μm, **B**, **E**–**J** = 50 μm. *p < 0.05, **p < 0.01, ***p < 0.001

Phagocytic activity of h-MSCs was examined by incubating h-MSCs with mCherry-m-Hepa1 DDCs. Laser confocal microscopy images and live cell images showed that 91.2 ± 3.5% and 93.5 ± 3.8% of GFP-h-MSCs had phagocytosed DDCs at 5 h and 20 h, respectively (Fig. 2A, B).

When h-MSCs were incubated with DDCs derived from m-cardiomyocytes, r-neural cells, or r-hepatic cells, no detectable levels of h-cardiac- (h*-NKX2,5*, h*-GATA-4*, h*-ANP*, and h*-TNT*), h-neural- (h-*FOXP2*, h-*PAX6*, h-*MATH1*, and h-*NEUN)*, and -hepatic-(h-*PROX1*, h-*KRT18*, h-*ALB,* and h-*AFP*) lineage markers were observed at any time-point through D21 in qPCR (data not shown).

On the other hand, h-MSCs incubated with apoptotic m-adipocyte DDCs newly expressed h-CCAAT enhancer binding protein beta (*CEBPB*) (*p* < 0.001) and h-peroxisome proliferator-activated receptor g (*PPARG*) (*p* < 0.05) at D3, reaching maximum levels at D14 (h*-CEBPB*; *p* < 0.001) and D21 (h*-PPARG*; *p* < 0.001), as assessed by qPCR (Fig. 2C). In addition, h-fatty acid binding protein 4 (*FABP4*) was newly expressed at D14 (*p* < 0.001) and reached the maximum level at D21 (*p* < 0.001). H-MSCs exhibited Oil Red O-positive lipid droplets in the cytoplasm (Fig. 2D) and immunostaining revealed that h-nuclei antibody(+) h-MSCs expressed FABP4 at D21 (Fig. 2E, S2).

When h-MSCs were incubated with apoptotic m-chondrocyte DDCs for 3 days and transferred to suspension culture, they newly expressed h-*SOX9* (*p* < 0.05), h-matrix metalloproteinase 13 (*MMP-13*) (*p* < 0.05), h-collagen type X alpha 1 chain (*COL10A1*) (*p* < 0.001), and h-aggrecan (*ACAN*) (*p* < 0.001) at D3 compared with naïve h-MSCs, which was further increased over time, except for h*-SOX9*, in qPCR (*p* < 0.001) (Fig. 2F). Expression of h-cardiac-(h*-NKX2,5*, h*-GATA-4*, h*-ANP*, and h*-TNT*), -neural-(h-*FOXP2*, h-*PAX6*, h-*MATH1*, and h-*NEUN)*, and -hepatic-(h-*PROX1*, h-*KRT18*, h-*ALB,* and h-*AFP*) lineage markers was consistently under the detection limits (data not shown). Alcian blue staining suggested the presence of glycosaminoglycans in h-Muse cells (Fig. 2G) and immunostaining revealed the expression of ACAN in h-MSCs confirmed to be positive for the h-nuclei antibody (Fig. 2H, S2), both at D21.

When h-MSCs were incubated with apoptotic-m-chondrocyte DDCs for 3 days and then co-cultured in suspension with a mouse-articular cartilage slice for 7 days, expression of the mature chondrocyte markers h-*MMP13*, h-*COL10A1,* h-*ACAN,* h-thrombospondin 4 (*THBS4*)*,* and h-SIX homeobox 1 (*SIX1*) was significantly upregulated compared with that at D21 (h-*COL10A1*, h-*ACAN*, and h-*SIX1*; *p* < 0.05, h-*MMP13* and h-*THBS4*; *p* < 0.01). Expression of h-collagen type II alpha 1 chain (*COL2A1*), undetectable in both naïve h-MSCs and h-MSCs at D21, became positive in qPCR (SI 8-Fig. S8A).

### Acceleration of neural cell marker expression in r-NSCs after incubating with apoptotic neural cell DDCs

GFP( +) r- NSCs were transferred to a poly-l-lysine-coated culture dish in medium without growth factors for 24 h and incubated with mCherry-*m*-Hepa1 DDCs. Consequently, 80.8 ± 7.5% and 88.5 ± 5.1% of GFP-r-NSCs showed phagocytic activity at 5 h and 20 h, respectively (Fig. 2I, J).

Markers for neuronal cells (r-neurogenic differentiation 1 [*NeuroD1*] and r-*Neurofilament*), oligodendrocytes (r-oligodendrocyte transcription factor [*Olig2*] and r-SRY-box transcription factor 10 [*Sox10*]), and astrocytes (r-glial fibrillary acidic protein [*Gfap*] and r-*Vimentin*) expressed in r-NSCs in adherent culture were all significantly upregulated after incubating with apoptotic h-neural cell DDCs, either at D3 (r-*Neurofilament*: *p* < 0.001, r-*Olig2*: *p* < 0.01, r-*Vimentin*: *p* < 0.05) or D7 (r-*NeuroD1*: *p* < 0.01, r-*Sox10* and r-*Gfap*: *p* < 0.001) and were maintained at D14 (all with *p* < 0.001), in qPCR (Fig. 2K).

When r-NSCs incubated with apoptotic-h-neural cell DDCs for the first 3 days were then co-cultured with a rat-brain tissue slice for 7 days, expression of the functional neural markers r-*Hcn2*, r-*Kcnb1*, and r-*Scn2a* was significantly upregulated compared with that at D14 (*p* < 0.001). R-*Psd95*, r-synaptophysin, and r-*Mbp*, undetectable in both naïve r-NSCs and r-NSCs at D14, became positive in qPCR (SI 8-Figure S8B). When r-NSCs were incubation with apoptotic m-cardiomyocyte- or apoptotic m-adipocyte-DDCs, not detectable levels of r-cardiac- (*Nkx2,5*, *Gata-4*, *Anp*, and *Tnt*) or r-adipocyte (*Sox9*, *Mmp13*, *Col10a1*, and *Acan*) markers were observed at any time-point through D21 in qPCR (data not shown).

### Relation between phagocytosis and differentiation in vitro

**Fig. 3:**
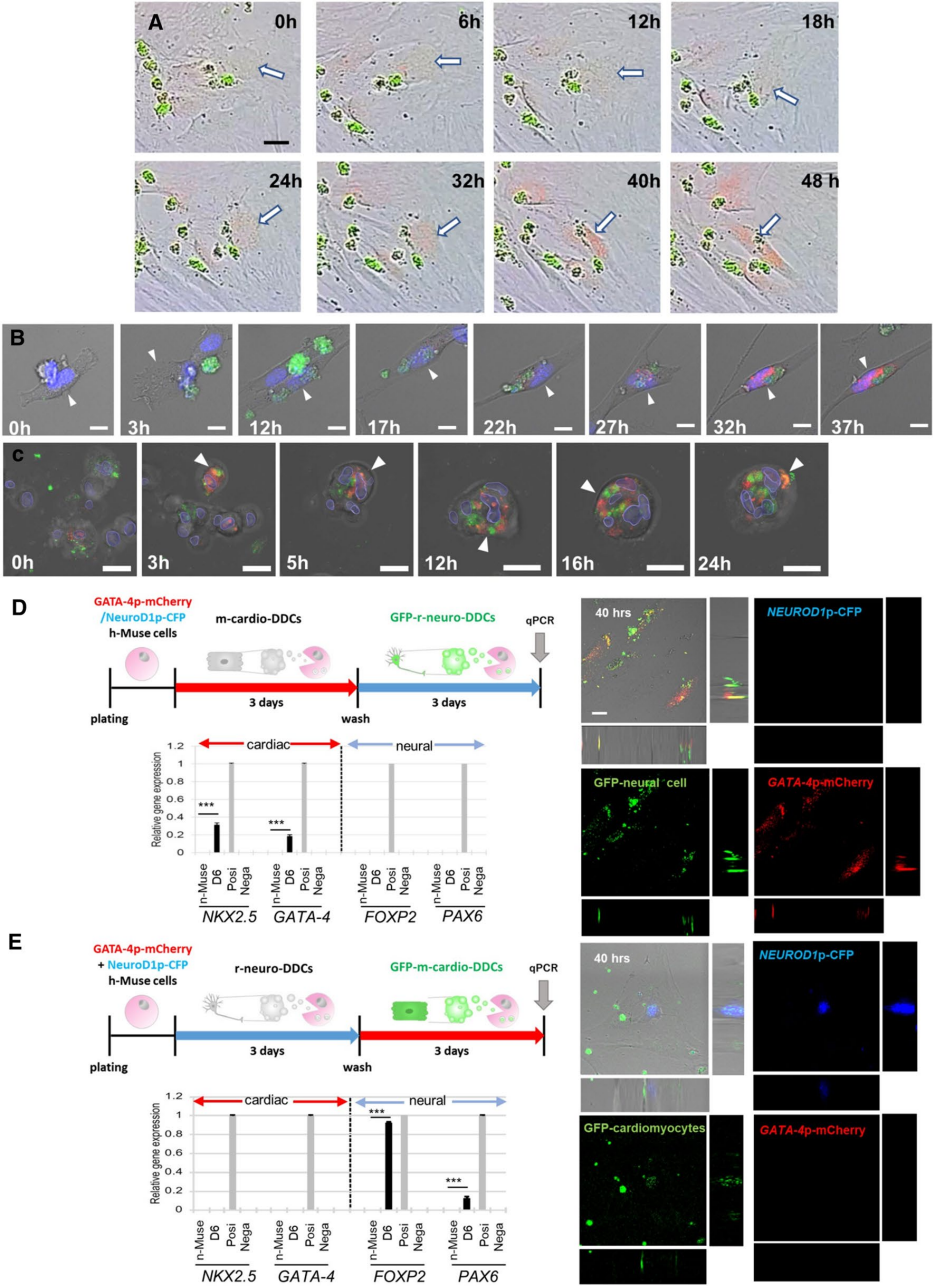
Lineage-specific differentiation of h-Muse cells and h-MSCs after phagocytosis in vitro. **A** *GATA-4*p-mCherry-h-Muse cells expressed mCherry and changed their morphology after uptake of apoptotic GFP-m-cardiomyocyte DDCs (see Movie 3). **B**, **C** Laser confocal microscopy live images of the same experiment as (**A**) in h-Muse cells (B) (see Movie 4). **C** *SOX9*p-mCherry-h-MSCs were incubated with m-chondrocyte DDCs (see Movie 5). **D**, **E** Cardiac- and neural-marker expression at D6, and laser confocal images at 40 h of (**D**) *GATA-4*p-mCherry/*NEUROD1*p-CFP-co-introduced h-Muse cells incubated with non-labeled-m-cardiomyocyte DDCs for 3 days, washed, and then incubated with GFP-rat-neural DDCs for another 3 days, and then, **E** the incubation order was reversed with non-labeled-rat-neural DDCs, followed by GFP-m-cardiomyocyte DDCs (mean ± SEM). qPCR; D6: *GATA-4*p-mCherry/*NEUROD1*p-CFP-co-introduced h-Muse cells at day 6, Positive control; human fetal-heart (for *NKX2.5* and *GATA-4*) and -brain (for *FOXP2* and *PAX6*) total RNA, Negative control: apoptotic cell fragments. Bars **A**–**E** = 25 μm. ***p < 0.001

The h-Muse cells transduced with the *GATA-4* promoter-mCherry (*GATA-4*p-mCherry) lentivirus were incubated with GFP-m-cardiomyocyte DDCs. Live images showed that h-Muse cells newly expressed mCherry after uptake of DDCs. Subsequently, the h-Muse cell morphology became similar to that of cardiomyocytes (Fig. 3A, Movie 3). The approximate time lag between the DDC uptake and mCherry expression in h-Muse cells was 24–36 h. Similarly, the expression of mCherry after uptake of DDCs in h-Muse cells was reproduced in laser confocal microscopy (Fig. 3B, Movie 4).

H-MSCs transduced with the *SOX9* promoter-mCherry (*SOX9*p-mCherry) lentivirus were incubated with GFP-m-chondrocyte DDCs and cultured in suspension. Live images in laser confocal microscopy demonstrated that the h-MSCs newly expressed mCherry after uptake of DDCs in suspension culture (Fig. 3C, Movie 5).

When h-Muse cells co-transduced with *GATA-4*p-mCherry and *NEUROD1*-promoter-CFP (*NEUROD1*p-CFP) were first incubated for 3 days with non-labeled m-cardiomyocyte DDCs, washed, and then incubated for another 3 days with GFP-r-neural cell DDCs, the h-Muse cells expressed mCherry but not CFP at D6, although they phagocytosed the GFP fragments. The cells were newly positive for h*-NKX2.5* and h*-GATA-4* by qPCR (both at *p* < 0.001, compared with naïve h-Muse cells), but not h*-FOXP2* or h*-PAX6* at D6 (Fig. 3D). When the incubation order was reversed with non-labeled-r-neural cell DDCs and GFP-m-cardiomyocyte DDCs, h-Muse cells expressed CFP but not mCherry, although they phagocytosed the GFP fragments, and expression of only h*-FOXP2* and h*-PAX6* was detected at D6 (*p* < 0.001) (Fig. 3E), suggesting that once the lineage-specific differentiation is triggered, subsequent phagocytosis of new DDCs from another cell lineage has limited effects on the direction of differentiation.

In the following in-depth analyses, h-Muse cells were used under the assumption that lineage-specific differentiation can be clearly traced in the triploblastic differentiation system.

### Single-cell RNA sequence analysis (scRNA-seq)

**Fig. 4:**
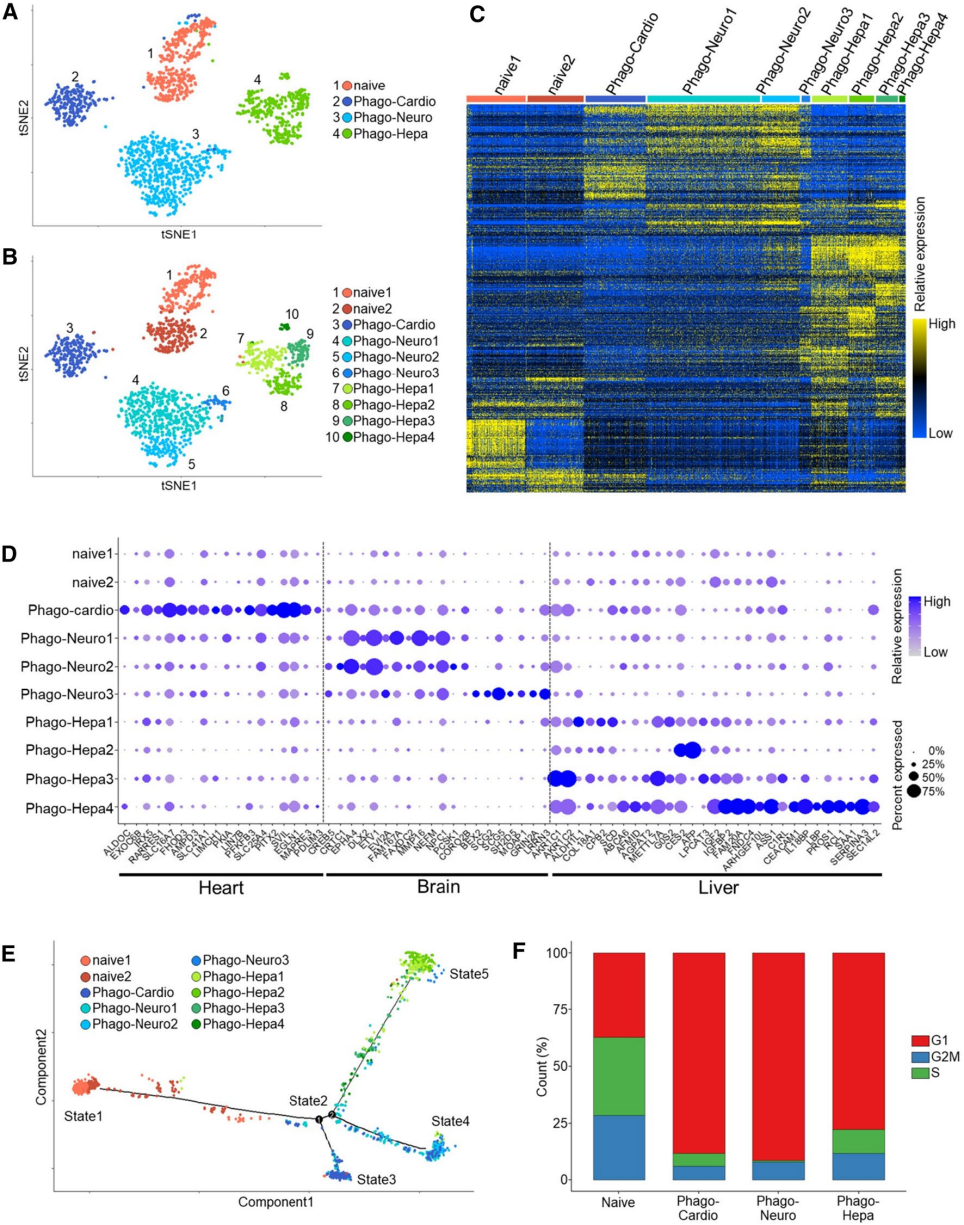
Single-cell RNA sequencing of h-Muse cells after incubating with mouse/rat-apoptotic fragments. **A**, **B** t-SNE visualization of naïve h-Muse cells (Naïve) and h-Muse cells after incubating with m-cardiomyocyte DDCs (Phago-cardio), r-neural cell DDCs (Phago-Neuro), and r-hepatic cell DDCs (Phago-Hepa). **C**, **D** Hierarchy heatmap (**C**) and dot plot of the lineage-specific marker genes (**D**) in each of the 10 clusters. In **D**, the minimum value of its accessibility is subtracted for each gene, and the result is divided by the maximum value of its accessibility. The dot size indicates the percent of cells in each cluster in which the gene of interest is accessible. The standardized accessibility level is indicated by the color intensity. **E** Monocle trajectory plots show 5 states. **F** The rate of G1, G2M, and S phases in Naïve-, Phago-cardio-, Phago-neuro-, and Phago-hepa-Muse cells

To verify the lineage-specific differentiation induced by phagocytosing DDCs, naïve h-Muse cells (Naïve-Muse cells) as well as h-Muse cells incubated with m-cardiomyocyte DDCs (Phago-cardio-Muse), r-neural cell DDCs (Phago-neuro-Muse), or r-hepatic cell DDCs (Phago-hepa-Muse) were subjected to scRNA-seq at 1 week. Single cells from each sample were processed for scRNA-seq using the TAS-seq protocol [32]. Overall, 1752 cells passed quality control with a mean of 5939 genes/cell and 87,322 reads/cell. Then, 120 low-quality cells comprising doublets and dying cells were removed from the analysis. Consequently, a total of 1632 single cells (404 Naïve-Muse cells, 250 Phago-cardio-Muse, 621 Phago-neuro-Muse, and 357 Phago-hepa-Muse) were used for further analysis. A t-distributed stochastic neighbor embedding (t-SNE) process revealed that Phago-cardio-Muse, Phago-neuro-Muse, and Phago-hepa-Muse cells were distinct from Naïve-Muse cells and also differed from each other (Fig. 4A). The number of differentially expressed genes (DEGs) in Phago-cardio-Muse cells, Phago-neuro-Muse cells, and Phago-hepa-Muse cells was 1235 (Up 732, Down 503), 1503 (Up 454, Down 1049), and 2497 (Up 1178, Down 1319), compared with naive Muse cells, respectively. According to the gene expression pattern, Naïve-Muse cells were further separated into two clusters, Phago-neuro-Muse into three clusters, and Phago-hepa-Muse into four clusters, while Phago-cardio-Muse remained a single cluster (Fig. 4B). The majority of Naive-Muse cell-cluster 1 was at G2M/S, whereas the majority of Naive-Muse cluster 2 was at G1, suggesting that the two clusters could be distinguished on the basis of their proliferative activity (SI 9-Fig. S9A). Subpopulation analysis demonstrated that Phago-neuro-Muse-clusters 1 and 2 predominantly expressed glial markers while Phago-neuro-Muse-cluster 3 expressed neuronal markers; Phago-hepa-Muse-cluster 1 tended to express endothelial markers, Phago-hepa-Muse-cluster 2 tended to express Kupffer cell markers, and Phago-hepa-Muse-clusters 3 and 4 tended to express hepatocyte-related markers (SI 9-Fig. S9B).

The differences in the gene expression signatures among the ten clusters are shown in the hierarchy heatmap in Fig. 4C. To explore the gene expression profiles across the ten clusters, we examined the accessibility of selected markers by referring to The Human Protein Atlas (http://www.proteinatlas.org) [33] (Fig. 4D). As the dot plot shows, the two Naïve-Muse cell clusters had lower gene accessibility associated with distinct lineages. Interestingly, Phago-cardio-Muse, Phago-neuro-Muse, and Phago-hepa-Muse cells exhibited a clear separation of clusters in terms of the overall accessibility of lineage-specific markers corresponding to the human heart, brain, and liver tissues, respectively (Fig. 4D). Notably, functional markers of the heart (such as PDZ and LIM domain 3 [*PDLIM3*], microtubule-associated protein RP/EB family member 3 [*MAPRE3*], Egl-9 family hypoxia inducible factor 1 [*EGLN1*]) [34–36], the brain (leucine-rich repeat neuronal 3 [*LRRN3*], glutamate ionotropic receptor NMDA type subunit 2A [*GRIN2A*], SH2 domain containing 5 [*SH2D5*], neuroendocrine convertase 1 [*NPC1*], secretogranin V [*SCG5]*) [37–40], and the liver (aldo–keto reductase family 1 member C1 [*AKR1C1*], *AKR1C2*, serpin family A member 3 [*SERPINA3*], protein S [*PROS1*], *AFP*) [41–44], were detected in Phago-cardio-Muse, Phago-neuro-Muse, and Phago-hepa-Muse cells, respectively (Fig. 4D).

Next, the Monocle R package was used to reconstruct the branched trajectory and revealed five cell states and two branch points (Fig. 4E). The two Naïve-Muse cell clusters mainly belonged to State 1. State 2 was located between the two branches corresponding to the branch points in States 3, 4, and 5, mainly comprising Phago-cardio-Muse, Phago-neuro-Muse, and Phago-hepa-Muse cells, respectively (Fig. 4E). When Naïve Muse cells branched from state 2 to state 3, state 4, and state 5, the number of genes varied by 457, 1222, and 298 genes, respectively. Cell cycle analysis revealed that cells at the G1 phase were overall higher among Phago-cardio-Muse, Phago-neuro-Muse, and Phago-hepa-Muse cells, than among Naïve-Muse cells (Fig. 4F, SI 9-Fig. S9A).

**Fig. 5:**
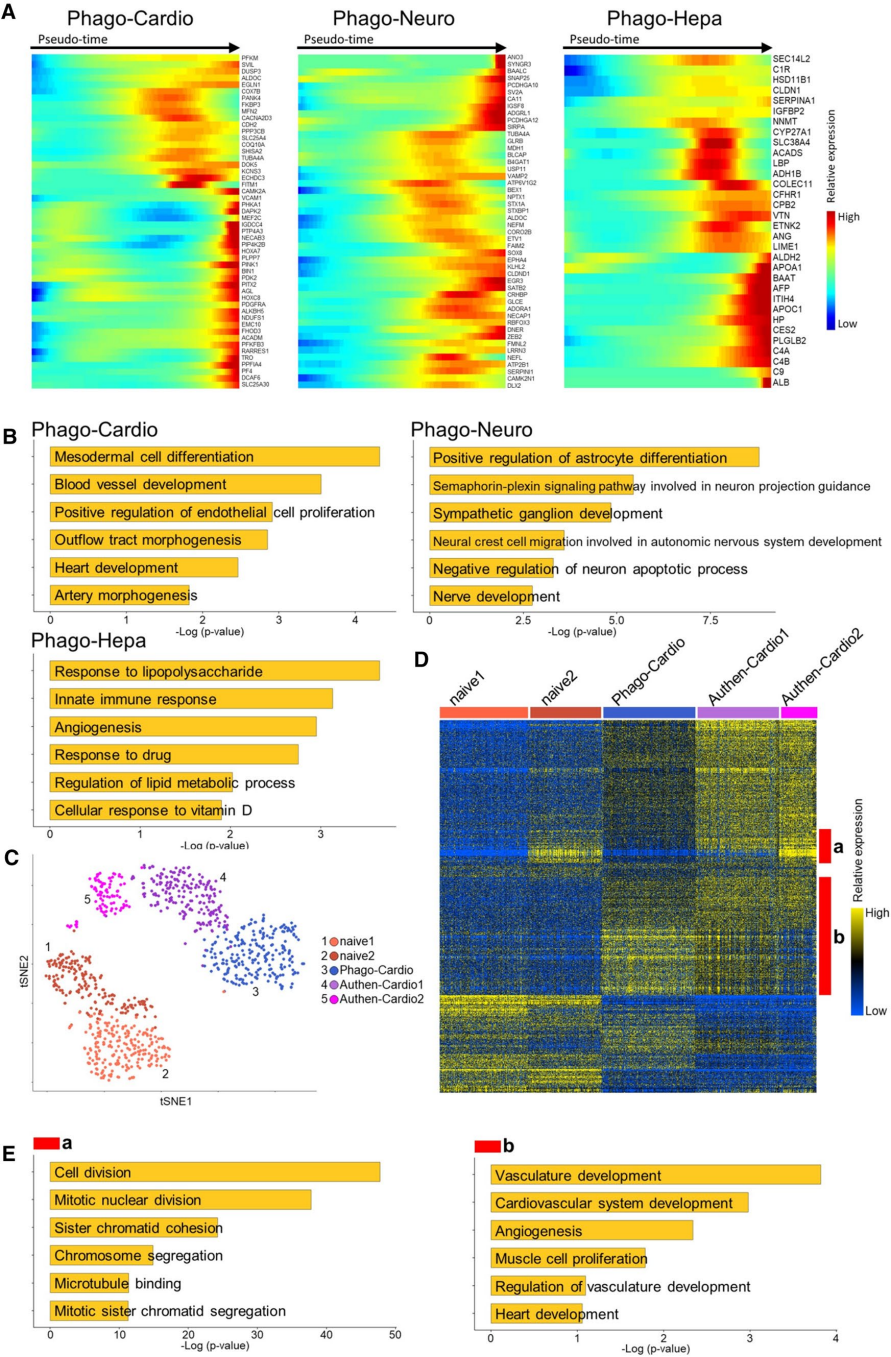
Single-cell RNA sequencing of h-Muse cells and comparison with authentic differentiated cells. **A** Pseudotemporal depiction of heatmap showing the expression level of lineage-specific genes (referenced to The Human Protein Atlas and BioGPS databases) during the trajectory. **B** GO term of DEGs between Naïve-Muse cells and each Phago-Muse group. **C**–**E** t-SNE visualization (**C**) and hierarchy heatmap (**D**) of 2 Naïve h-Muse cell clusters (Naïve-1, -2), Phago-cardio-Muse (Phago-cardio), and adult human cardiomyocyte cell line (Authen-Cardio-1, -2). **E** GO term for red line A (signals detected in Naïve-2 and Authen-Cardio-2) and b (Phago-cardio-Muse and Authen-cardio-clusters 1 and 2) in (**D**) is shown

Differential expression analysis with a pseudo-time line referring to The Human Protein Atlas and BioGPS (http://biogps.org/#goto=welcome) [45] demonstrated that cardiac-, neural-, and hepatic-related genes were activated in the Phago-cardio-Muse, Phago-neuro-Muse, and Phago-hepa-Muse cells, respectively (Fig. 5A, SI 9-Fig. S9B). Gene Ontology (GO) term analyses of DEGs between Naïve-Muse cells and each Phago-Muse group revealed lineage-related GO terms (Fig. 5B, SI 9-Fig. S9C). The expression of complement-related genes (complement C1r [*C1R*], complement C1s [*C1S*], complement C4B [*C4B*], complement factor B [*CFB*], and complement factor H related 1[*CFHR1*]) was increased in Phago-hepa-Muse cells compared with naïve Muse cells (SI 9-Fig. S9D).

To compare the similarities and differences between the committed h-Muse cells after phagocytosis and authentic differentiated cells, Phago-cardio-Muse and adult human cardiomyocyte cell line (Authen-Cardio) were analyzed. The t-SNE analysis revealed that Phago-cardio-Muse and the two Authen-Cardio clusters were similar to each other, whereas they were distinct from the Naïve-Muse cell clusters (Fig. 5C).

In the hierarchy heatmap and GO terms, genes related to cell division were detected in Naive-Muse cell-cluster 2 and Authen-Cardio-cluster 2 (red line a in Fig. 5D, E), while genes related to cardiac differentiation were identified in both Phago-cardio-Muse and Authen-cardio-clusters 1 and 2 (red line b in Fig. 5D, E).

To compare the similarities and differences between the committed h-Muse cells after phagocytosis and human fetal cells, human fetal transcriptome data obtained by scRNA seq (NCBI Gene Expression Omnibus [GSE15732]) was compared with Phago-cardio-, -neural-, and -hepa-Muse cells. In the hierarchy heatmap and GO terms, cardiac-related genes were found in both the human fetal transcriptome and Phago-cardio-Muse cells (SI 10-Fig. S10A, red line), neural-related genes were found in the human fetal transcriptome and Phago-neuro-Muse cells (SI10-Fig. S10B, red line), and hepatic-related genes were found in the human fetal transcriptome and Phago-hepa-Muse cells (SI10-Fig. S10C, red line).

In order to verify the validity of the scRNA seq data of one biological replicate, we select several genes that were upregulated in the scRNA seq data (Phago-Cardio-Muse: *EGLN1*, *SVIL*, *SLC16A7*; Phago-neuro-Muse: *EPHA4*, *ETV1*, *FAM167A*; Phago-hepa-Muse: *AKR1C1*, *SERPINA3*, *CEACAM1*) and those genes were analyzed in qPCR with three biological replicates at 7 days after phagocytosis. These results showed that all genes were upregulated in Phago-Muse compared to naïve Muse cells with statistical significance (Fig. S11).

### Next-generation sequencing (NGS)

To confirm global gene expression after DDCs phagocytosis, NGS analysis was performed in naïve h-MSCs (Naïve MSCs) and h-MSCs incubated with m-adipocyte DDCs (Phago-MSCs). More than 90 million reads were obtained from each replicate. Unmapped reads were excluded, and the remaining reads were mapped to 28,695 genes. Gene expression was calculated individually for each sample and then, compared between samples. The DEGs results between Naïve-MSCs and Phago-MSCs are shown in Fig. S12A. The expression of mesenchymal markers was decreased and that of adipogenesis markers was increased in Phago-MSCs compared with naïve MSCs (SI 12-Fig. S12B). GO term analysis of DEGs between Naïve-MSCs and Phago-MSCs revealed adipogenesis-related GO terms (SI 12-Fig. S12C).

Similarly, NGS analysis was conducted in naïve r-NSCs (Naïve-NSCs) and r-NSCs incubated with h-neural cell DDCs (Phago-NSCs). More than 70 million reads were obtained from each replicate. Unmapped reads were excluded, and the remaining reads were mapped to 20,333 genes. The DEG results between Naïve-NSCs and Phago-NSCs are shown in Figure S12D. The expression of neurogenesis markers was increased in Phago-NSCs compared with Naïve-NSCs (SI 12-Fig. S12E). GO term analysis of DEGs between Naïve-NSCs and Phago-NSCs revealed neurogenesis-related GO terms (SI 12-Fig. S12F).

### Effect of phagocytosis inhibition on in vitro differentiation

**Fig. 6:**
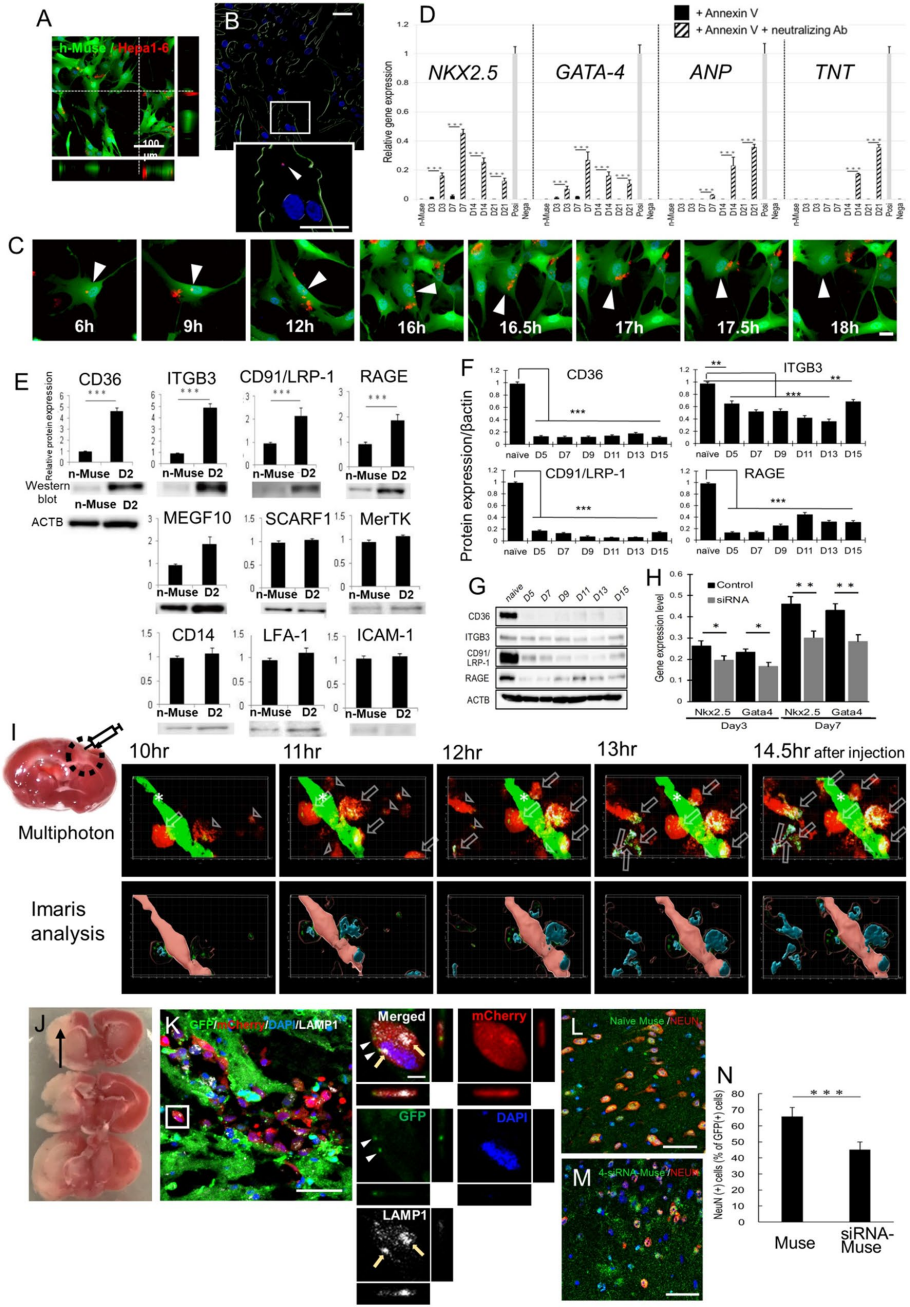
Effect of phagocytosis suppression on h-Muse cell differentiation in vitro and in vivo. **A**–**C** GFP-h-Muse cells incubated with annexin V-treated apoptotic mCherry-m-Hepa1 DDCs. **A** at 20 h with Z-series 3D construction (see Movie 6). **B** Analysis of (**A**) by Bitplane Imaris software. The inset shows high magnification of the box. Arrowhead: phagocytosed signal. **C** mCherry-phagosome in GFP-h-Muse cells was released back to the extracellular space (see Movie 6). **D** qPCR (mean ± SEM) of h-cardiac markers in naïve h-Muse cells (n-Muse) and h-Muse cells after incubation with m-cardiomyocyte DDCs pretreated with annexin V or with annexin V + anti-annexin V neutralizing antibody. DDCs were used as a negative control (Nega), and human fetal heart total RNA as a positive control (Posi). **E** Western blot (mean ± SEM) of each phagocytosis receptor in h-Muse cells in the naïve state and D2 after incubation with m-cardiomyocyte DDCs. Beta-actin (ACTB) is common to all the blots. The signal in the naïve h-Muse cells is set as 1. **F**, **G** Western blot (mean ± SEM) demonstrated reduction in phagocytosis receptor expression in 4-siRNA-h-Muse cells until D15. **H** h-cardiac marker expression (mean ± SEM) in 4-siRNA-h-Muse cells after incubation with m-cardiomyocyte DDCs. **I**–**M** Neuronal differentiation of h-Muse cells after phagocytosis in vivo. C57BL/6-Tg (CAG-EGFP) mouse focal stroke model 10 ~ 14.5 h after topical injection of mCherry-h-Muse cells transfected with *NEUROD1*-promoter-CFP. **I** TTC staining of the mouse focal brain ischemia model. Multiphoton laser scanning microscopy images and Bitplane Imaris software analysis of the infarct area (see Movie 7). *: vessels, white arrows: *NEUROD1*-CFP-positive mCherry-h-Muse cells, white arrowheads: *NEUROD1*-CFP-negative mCherry-h-Muse cells. **J** TTC staining of cerebral ischemia at 2 days after stroke. **K** Left: Large image of mCherry-h-Muse cells in the post-infarct mouse brain tissue at D1 contained GFP(+)-host-derived fragments (arrowheads) and LAMP-1(+) phagosomes (arrows). Right: Enlarged image of the white square. **L**, **M** NeuN (+) in GFP + cells in naïve-GFP-h-Muse cell injected- (L) and GFP-4-siRNA-h-Muse cell injected- (M) brain at D7. **M** The ratio of NEUN + in naïve-GFP-h-Muse cells and GFP-4-siRNA-h-Muse cells at D7 (mean ± SEM). Bars: **A** = 100 μm, **B**, **C**, **I**, **L**, **M** = 50 μm. **K** left = 50 μm, **K** right = 5 μm. *p < 0.05, **p < 0.01, ***p < 0.001

Uptake of apoptotic cells is known to be inhibited by annexin V, which interferes with phosphatidyl serine (PS)-dependent recognition of dying cells [46]. Annexin V administration did not affect the survival rate of h-Muse cells (SI 13-Figure S13A). mCherry-m-Hepa1 DDCs were first incubated with annexin V and then supplied to GFP-h-Muse cells. Compared to cells without annexin V, the rate of phagocytosis by h-Muse cells incubated with annexin V-treated DDCs decreased from ~ 87% to 13.6 ± 2.4% at 5 h and from ~ 91% to 14.0 ± 4.0% at 20 h (*p* < 0.01) when cells containing even 1 mCherry( +) particle were counted as positive (Fig. 6A, B, Movie 6). Even though a small number of h-Muse cells still exhibited phagocytic activity, larger phagosomes in many cases were not fragmented into particles, and this tendency was maintained even at D3. In some cases, the large phagocytosed DDCs were released into the extracellular space without fragmentation (Fig. 6C, Movie 6).

In contrast to Fig. 1B, incubation of h-Muse cells with annexin V-treated m-cardiomyocyte DDCs showed very faint h-*NKX2.5* and h-*GATA-4* expression at D3 and D7, and h-*ANP* and h-*TNT* remained under the detection limit up to D21 (Fig. 6D). Similarly, incubation of h-MSCs with annexin V-treated m-adipocyte DDCs and incubation of r-NSCs with annexin V-treated h-neural cell DDCs led to a statistically significant reduction in the gene expression levels of h-adipogenic (SI 13-Fig. S13B) and r-neural cell markers (SI 13-Fig. S13C), respectively, compared to those without annexin V treatment.

In h-Muse cells, the expression of human-cardiac markers was revived, however, by incubation of m-cardiomyocyte DDCs with a mixture of annexin V and annexin V-neutralizing antibody, followed by incubation with h-Muse cells (all with *p* < 0.001) (Fig. 6D), suggesting the involvement of phagocytosis in the commitment to the phagocytosed cell lineage.

NGS data showed that incubation of h-MSCs with annexin V-treated m-adipocyte DDCs (Phago-MSCs + Annexin V) showed a gene expression pattern similar to that of naïve MSCs rather than to Phago-MSCs, suggesting that h-MSC differentiation was inhibited by pretreatment of DDCs with annexin V (SI 12-Fig. S12B). Incubation of r-NSCs with annexin V-treated h-neural cell DDCs (Phago-NSCs + Annexin V) showed a gene expression trend similar to that of naïve-NSCs rather than to Phago-NSCs (SI 12-Fig. S12E).

### Phagocytic receptor expression

Receptors related to phagocytosis in macrophages [47, 48] were investigated in h-Muse cells by Western blotting. Naïve h-Muse cells expressed the receptor for advanced glycation end-products (RAGE), which directly binds to PS; as well as Mer Tyrosine Kinase (MERTK), integrin alpha-V/beta-3 (ITGB3), cluster of differentiation 36 (CD36), and cluster of differentiation 91/low density lipoprotein receptor-related protein 1 (CD91/LRP-1), which indirectly bind to PS. Multiple EGF-like domains 10 (MEGF10) and scavenger receptor class F member 1 (SCARF1), which directly bind to C1q on apoptotic cell membranes, as well as cluster of differentiation 14 (CD14), lymphocyte function-associated antigen 1/cluster of differentiation 11a (LFA-1/CD11a), and intercellular adhesion molecule 1 (ICAM-1), were also detected in naïve h-Muse cells (Fig. 6E, Fig. S19). All of these receptors were either maintained at a similar level or upregulated at D2 after exposure to m-cardiomyocyte DDCs. Notably, CD36 and ITGB3 expression was ~ 4.5-fold higher (*p* < 0.001) and CD91/LRP-1 and RAGE expression was ~ twofold higher (*p* < 0.001) in h-Muse cells at D2 after DDC exposure compared with naïve h-Muse cells (Figs. 6E, S19). On the other hand, T cell immunoglobulin and mucin domain containing 4 (TIMD4-1, -2, and -3), as well as brain angiogenesis inhibitor 1 (BAI1), CD300 molecular like family member F (CD300LF), stabilin-2 (STAB2), and intercellular adhesion molecule 3 (ICAM-3), the major phagocytic receptors in macrophages [49], were under the detection limits in h-Muse cells in both qPCR and Western blotting (data not shown). The expression profiles of h-MSCs and r-NSCs were similar to those in h-Muse cells, but the major receptors activated by exposure of h-MSCs to m-adipocyte DDCs were CD36 and SCARF1; and of r-NSCs to h-neural cell DDCs were CD36, CD91/LRP-1, MERTK, CD14, and ICAM (SI 13-Figs. S13D, E, S20). Therefore, the phagocytosis receptor subsets expressed by h-Muse cells, h-MSCs, and r-NSCs are different from those expressed by macrophages.

To assess the involvement of phagocytosis receptors, CD36, ITGB3, CD91/LRP-1, and RAGE, in lineage-specific differentiation of h-Muse cells, small interference RNA (siRNA) for these receptors was introduced into h-Muse cells and then, the cells were exposed to m-cardiomyocyte DDCs. Both h-*NKX2.5* and h-*GATA-4* showed significant decreases in CD36- and ITGB3-siRNA (*p* < 0.05), and significant decreases in either h-*NKX2.5* or h-*GATA-4* (p < 0.05) in CD91/LRP-1- and RAGE-siRNA on D7, on the basis of an on-target effect (SI 13-Figs. S13F, S21). We also investigated the effect of simultaneous suppression of 4 receptors: CD36, ITGB3, CD91/LRP-1, and RAGE, in h-Muse cells (4-siRNA-h-Muse) (Figs. 6F, G, S19). By using m-cardiomyocyte DDCs, we confirmed that marker expression of both h-*NKX2.5* and h-*GATA-4* at D3 (*p* < 0.05) and D7 (*p* < 0.01) was suppressed in 4-siRNA-h-Muse cells (Fig. 6H).

### Phagocytosis and differentiation in vivo

For the in vivo experiment, mCherry-h-Muse cells introduced with *NEUROD1*p-CFP were topically injected into the infarct area of a C57BL/6-Tg (CAG-EGFP) mouse focal ischemic stroke model 24 h after ischemia onset (Fig. 6I). In time-lapse imaging with multiphoton laser scanning microscopy followed by Bitplane Imaris software analysis, mCherry-h-Muse cells newly expressed *NEUROD1*p-CFP after phagocytosing GFP( +) host brain tissue fragments, while a small number of mCherry-h-Muse cells that did not phagocytose the fragments remained *NEUROD1*p-CFP-negative (Fig. 6I, Movie 7). In the sham group, no autofluorescence or artifact in the blue-color code (background for *NEUROD1*p-CFP) and red-color code (background for mCherry) was observed (SI14-Fig. S14A).

Next, 5 × 10^4^ GFP-labeled naïve h-Muse cells and GFP-labeled 4-siRNA-h-Muse cells were stereotaxically injected into the post-infarct area in a bilateral common carotid artery occlusion (BCCAO) model 2 days after onset (Fig. 6J). At D1, injected mCherry-h-Muse cells contained both GFP(+)-host brain tissue fragments and LAMP-1-positive phagosomes in the cytoplasm, suggesting that h-Muse cells have phagocytic activity in vivo (Fig. 6K). At D7, 4-siRNA-h-Muse cells showed significantly lower NEUN-positivity than naïve h-Muse cells in the post-infarct tissue (*p* < 0.001; Fig. 6L–N). In the sham group, no autofluorescence or artifact in the green-color code was observed (SI14-Fig. S14B, C).

### Detection of DDC-derived contents in the h-Muse cell cytoplasm and nucleus after phagocytosis

**Fig. 7:**
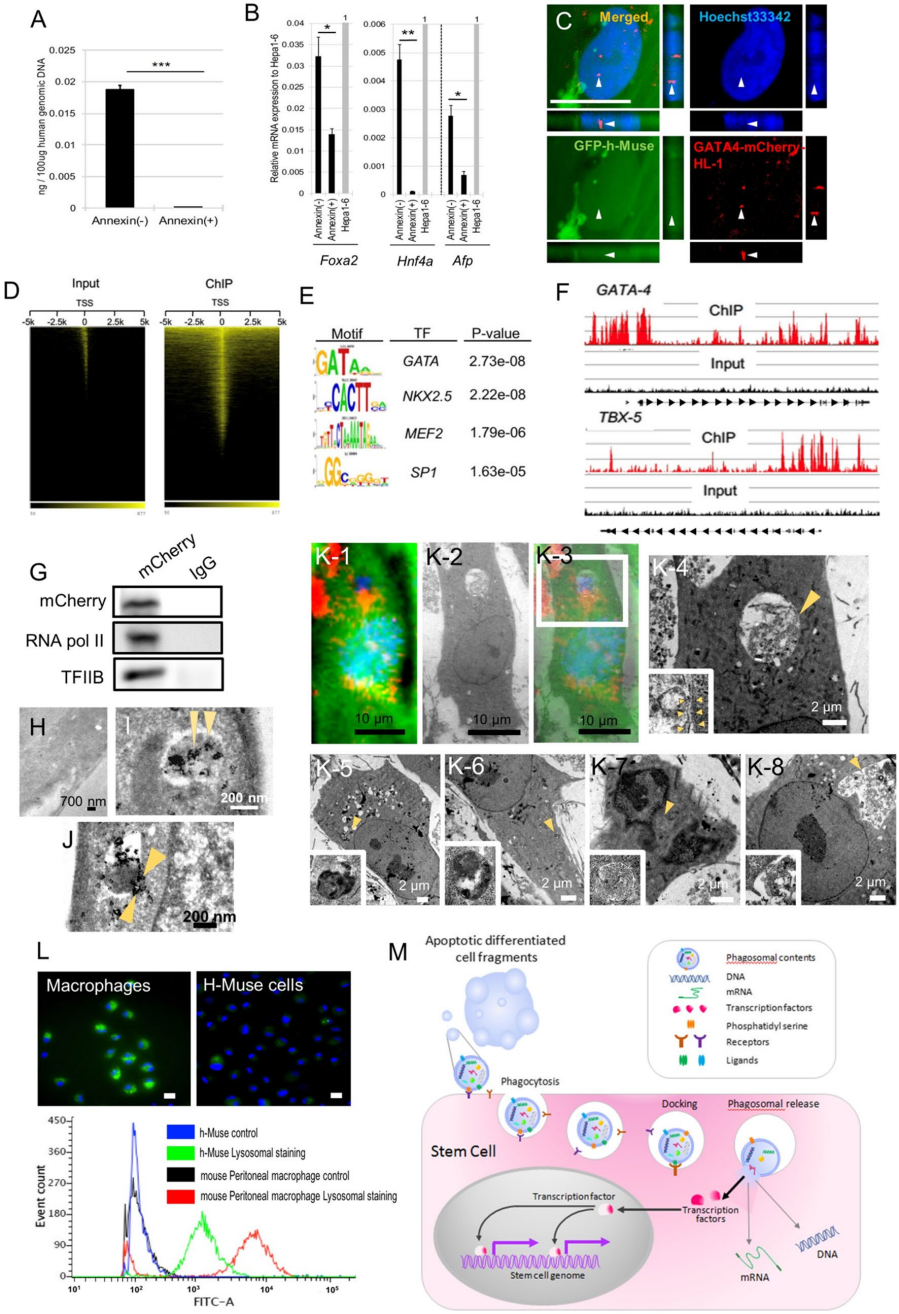
Detection of DDC-derived DNA, mRNA, and transcription factors in h-Muse cells after phagocytosis. **A** Mouse genome and **B** mouse mRNA, *Foxa2, Hnf4*, and a-fetoprotein (*Afp*), in h-Muse cells incubated with m-Hepa1 DDCs either with or without annexin V-pretreatment (1 day). m-Hepa1-6 cells (Hepa1-6) as positive control. Graphs: mean ± SEM. **C** Phagocytosed signal derived from GATA-4-m-HL-1 DDCs located in the nucleus of GFP-h-Muse cells at 36 h. Bar = 25 μm. **D**–**G** H-Muse cells were incubated with GATA-4-m-HL-1 DDCs. **D**–**F** ChIP seq for HA tag-GATA-4 in h-Muse cells after incubation with HA tag-GATA-4-m-HL-1 DDCs. **D** Heatmap analysis of ChIP-seq signals ± 5 kb around the nearest TSS. **E** Transcription factor recognition motifs most significantly enriched in HA tag-*GATA4* peaks. **F** Enrichment of sequencing tags was identified upstream of GATA-4 and Tbx5 in the ChIP samples (red) compared with input (black). **G** GATA-4-mCherry fusion protein in the h-Muse nuclear fraction of 8 h after incubating with apoptotic GATA-4-m-HL-1 DDC fragments was co-immunoprecipitated with anti-mCherry antibody or control IgG. mCherry, RNA Polymerase II and transcription factor II B were detected. **H**–**J** Immunoelectron microscopy for anti-mCherry in h-Muse cells incubated with apoptotic mCherry-m-Hepa1-6 DDC fragments for 1 day. Arrowheads indicate mCherry labeled by nanogold. **H** Is negative control. **I** Shows a mCherry(+) (arrowheads) phagosome separated by a narrow space in the h-Muse cell cytoplasm. **J** Arrowheads suggest the fusion of outer and inner cell membranes of phagosomes to release mCherry-positive contents into the cytoplasm of h-Muse cells. **K** TEM (K-2, K-4 ~ K8) merged with laser confocal microscopic image (K-1, K-3) in GFP-h-Muse cells incubated with apoptotic mCherry-m-Hepa1-6 DDC fragments for 12 ~ 24 h. H-Muse cells showed a phagosome containing DDCs. Double cell membrane of the phagosome was observed in K-4 (inset). **L** Green fluorescence indicates lysosomal activity of mouse peritoneal macrophages and h-Muse cells. Lysosomal activity is also shown by cell sorting. **M** Putative mechanism of how phagocytosis regulates commitment of stem cells (for example, Muse cells) to the phagocytosed cell lineage. DDC-derived transcription factors, proteins, DNA, and mRNA are released into the cytoplasm. Apoptotic cell-derived transcription factors might translocate to the nucleus and function directly in the stem cell. Bars: **H** = 700 nm, **I**, **J** = 200 nm, **K**1-3 = 10 μm, **K**4-8 = 2 μm. *p < 0.05, **p < 0.01, ***p < 0.001

DNA, RNA, and proteins (i.e., transcription factors) are the major components of phagosomes. We first examined whether DNA, represented by the DDC-derived genome, is detectable in h-Muse cells. GFP-h-Muse cells were incubated with mCherry-m-Hepa1 DDCs for 1 day, then the GFP-h-Muse cells containing mCherry-phagosomes were selected by cell sorting, and the DNA was collected from the whole cell fraction. The mouse genome was detected using a mouse-specific probe that was confirmed to not cross-react with the human genome. The h-Muse cells contained the mouse genome at D1, and the amount of mouse genome was substantially reduced by pretreating the DDCs with annexin V (*p* < 0.001; Fig. 7A).

Similarly, mouse mRNA was investigated at D1 after incubation with mCherry-m-Hepa1 DDCs. In this case, the cytoplasmic fraction of h-Muse cells was collected and then analyzed. mRNA for mouse-specific forkhead box A2 (*Foxa2*), hepatocyte nuclear factor 4 alpha (*Hnf4a*), and *Afp* was detected, while these signals were substantially reduced (*Foxa2* and *Afp*; *p* < 0.05, *Hnf4a*; *p* < 0.01) when the DDCs were pretreated with annexin V (Fig. 7B).

DDC-derived transcription factors are assumed to be present in h-Muse cells because the DDC-derived genome was detected in h-Muse cells (Fig. 7A). To confirm this, we transduced m-HL-1 cells expressing GATA-4-mCherry fusion protein (GATA-4-m-HL-1), as shown in Figure S15A in SI 15. GFP-h-Muse cells were then incubated with the GATA-4-m-HL-1 DDCs. GATA-4-mCherry-fusion protein was taken up by the GFP-h-Muse cells and distributed to the cytoplasm (SI 15-Fig. S15B, C) and nucleus (Fig. 7C). Thus, DNA, mRNA, and proteins (transcription factors) derived from DDCs were recovered within the h-Muse cells after incubation. Annexin V-treatment suppressed phagocytosis of GATA-4-m-HL-1 DDCs in h-Muse cells (SI 15-Fig. S15D, E).

Chromatin immunoprecipitation sequencing (ChIP-seq) for the anti-HA tag antibody was performed using the genome collected from h-Muse cells that were incubated with HA tag-GATA-4-m-HL-1 DDCs for 16 h. More than 60 million reads were obtained from each replicate. Unmapped reads were excluded, and the remaining reads were mapped to 2445 genes. The distribution of enriched peaks was compared with the random peak found from the input fraction of chromatin, showing that the ChIP-seq successfully enriched multiple sites near the activation sites of transcription across the entire genome (Fig. 7D). The analysis revealed a high enrichment of GATA motifs. Other highly enriched binding motifs included those for transcription factors such as *NKX2.5*, myocyte enhancer factor 2 (*MEF2*), and sp1 transcription factor (*SP1*), known to coordinately regulate cardiomyocyte differentiation with *GATA-4* [50] (Fig. 7E). The detected ChIP-seq signal was also mapped upstream of genes involved in cardiomyocyte differentiation, such as *GATA-4* and t-box transcription factor 5 (*TBX5*) (Fig. 7F).

We compared the ChIP-seq data of h-Muse cells (16 h incubation with DDCs) with that of human iPS cell-derived cardiomyocytes 6 days and 32 days after differentiation induction (obtained from CBI Gene Expression Omnibus [GSE159411, GSE85631]), respectively [51, 52]. There was an overlap of only 30 GATA-4 binding sites when compared with day 32 mature iPS cell-derived cardiomyocytes (SI 16, Fig. S16A), but was increased to 157 when compared with day 6 iPS cell-derived cardiomyocytes (SI 16, Fig. S16B).

Furthermore, immunoprecipitation of GATA-4-mCherry in the nuclear fraction of h-Muse cells revealed co-precipitation of transcriptional machineries, RNA polymerase II and transcription factor II B, suggesting that apoptotic cell-derived transcription factors were involved in the assembly of transcription machineries in h-Muse cells (Figs. 7G, S22).

One possible mechanism of differentiation is that differentiation-related transcription factors originally active in the DDC genome are taken up into stem cells by phagocytosis and translocated to the nucleus to bind to the genome, where they participate in the induction of differentiation to the DDC-cell type. If so, a critical question remains, because phagosomes are wrapped by two cell membranes, that is, the original DDC- and stem cell-derived membranes are separated by a space, how are the contents of the phagosomes discharged into the cytoplasm? To address this question, we performed anti-mCherry immunoelectron microscopy on phagocytosed mCherry-m-Hepa1 DDCs in the GFP-h-Muse cell cytoplasm at D1. We identified the presence of DDC-derived mCherry(+) phagosomes covered by inner DDC-derived and outer h-Muse cell-derived cell membranes, separated by a narrow space, in the h-Muse cell cytoplasm (Fig. 7H, I). In some phagosomes, we observed the fusion of the outer h-Muse cell-derived and inner DDC-derived membranes. This membrane fusion is speculated to be accompanied by the release of phagosome contents into the h-Muse cytoplasm (Fig. 7J). Combining laser confocal microscopy and transmission electron microscopy (TEM) (method described in SI 17), mCherry-m-Hepa1 DDCs were internalized in the cytoplasm of GFP-h-Muse cells (Fig. 7K, SI 17-Fig. S17). The internalization in Fig. 7K was similar to previously reported phagosomes [53].

Generally, phagosomes in macrophages fuse with lysosomes and the contents are digested. Notably, h-Muse cells, which are non-professional phagocytes, have lower lysosomal activity than mouse peritoneal macrophages (Fig. 7L), suggesting that the phagosomes in h-Muse cells have a greater chance of escaping digestion than the phagosomes in macrophages.

## Discussion

The findings of the present study revealed that: (1) phagocytosis-induced differentiation of Muse cells and MSCs to the same lineage as that of the phagocytosed apoptotic differentiated cells, but not other lineages, and promoted differentiation of NSCs into the neural lineage; (2) differentiation was restricted to the inherent capabilities of the stem cells. Muse cells differentiated into triploblastic-lineages, MSCs into adipogenic-/chondrogenic-lineages, and NSCs into neural-lineages; (3) phagocytosis was driven by different receptor subsets than those of macrophages, and inhibition of phagocytosis either by siRNA targeting the phagocytosis receptor or annexin V-treatment impeded the differentiation; (4) apoptotic cells, but not intact cells, were active in this process; (5) the differentiation process had a rapid time course compared with that of cytokine-induced differentiation in vitro, which usually takes at least several weeks [8–10, 54, 55]; (6) one potential mechanism of the differentiation is the direct utilization of machineries, including transcription factors, that originally functioned in the phagocytosed differentiated cells; and (7) the same sequence likely occurred for the in vivo differentiation, as suggested by Muse cells. The putative mechanism for inducing lineage-specific differentiation by phagocytosis is summarized in Fig. 7M.

The scRNA-seq demonstrated that, compared with the naïve state, Muse cells after phagocytosing DDCs expressed different gene sets that corresponded to the DDC-lineage. The differentiation trajectory revealed how Muse cells branched after phagocytosing cardiac-, neural-, or hepatic-DDCs (Fig. 4E). The expression of lineage-specific functional markers (Fig. 4D) and the high proportion of G1 cells among Phago-cardio-, Phago-neuro-, and Phago-hepa-Muse cells phase (Fig. 4F) suggested the differentiation trend of Muse cells after phagocytosis. Interestingly, the gene sets expressed by Phago-cardio-Muse cells were closer to those of Authen-Cardio than Naïve-Muse cells, and genes related to the cardiac lineage were observed in both Phago-cardio-Muse and Authen-Cardio cells (Fig. 5C–E). Together with scRNA-seq, data from qPCR, immunocytochemistry, and a calcium-influx response to biochemical depolarization (Fig. 1B–I) suggested that Muse cells acquired a certain level of functionality after phagocytosing DDCs, while the impact of phagocytosis seemed to be more potent in the early stage of differentiation than in the later stage. In contrast, scRNA-seq results showed that gene expression of phago-Muse cells did not completely match that of mature cells (Authen-cardio), suggesting that phagocytosis alone is not sufficient for differentiation maturation.

Also of interest is that the simple administration of conditioned medium, cell extracts, or extracellular vesicles was not sufficient to activate the lineage-specific differentiation (SI 6-Fig. S6), and the phagocytic actions could not be substituted for by these factors in vitro. These observations, however, do not exclude the involvement of these factors in the differentiation maturation process. For example, the expression of functional markers was promoted in Muse cells when co-cultured with a damaged tissue slice that might have supplied cytokines and extracellular vesicles (Fig. 1D, H). Furthermore, extracellular vesicles are classified as small exosomes, large exosomes, exomeres, and supermeres [56]. Because the contents and efficiency of uptake differs between cells, different outcomes may be obtained with each refined fraction rather than the whole extracellular vesicles. It is also possible that conditioned medium and cell extracts at a higher concentration than that used in this study induce the differentiation of Muse cells.

### Dynamics of phagocytosed DDCs

The docking of phagosomes and cytoplasm, called ‘back-fusion,’ is reported in bacterial phagocytosis and allows for the release of bacteria from phagosomes into the cytoplasm [57, 58]. The precise mechanism is still under debate, but some possible mechanisms are proposed: bacteria-derived lipase and/or toxins, or channels that act on the phagosomal membrane allow bacteria to escape from phagosomes [57]. Our study demonstrated that one possible mechanism of phagosomal release is fusion of the inner- and outer-phagosomal membranes. A similar fusion of the two membranes is suggested in an extracellular vesicle model [59]. The precise mechanism of how the fusion of the two membranes is controlled is unknown and requires further study.

In macrophages, phagosomes immediately fuse with lysosomes and are digested [47]. As shown by Muse cells, however, the lower lysosomal activity than macrophages may provide phagosomes a greater chance to release their contents into the cytoplasm before being digested following fusion with lysosomes (Fig. 7L).

### The putative fate of phagosome-derived DNA, RNA, and proteins after phagocytosis

DDC-derived genomic DNA, mRNA, and proteins (transcription factors) were detected in the Muse cells after phagocytosis. Different from DNA and mRNA, proteins are able to participate directly and rapidly in the lineage-specific differentiation if they are released into the cytoplasm without digestion. GATA-4-mCherry fusion protein derived from apoptotic GATA-4-m-HL-1 was detected in the Muse cell cytoplasm (SI 15-Fig. S15B, C) and nucleus (Fig. 7C) and was suggested to bind to promoter regions of cardiomyocyte-related genes (Fig. 7D–F) where it might have recruited transcriptional machineries such as RNA polymerase II and transcription factor II B (Fig. 7G). However, the ChIP-seq data for GATA-4 in Phago-Muse cells did not exhibit much overlap with that in iPS cell-derived cardiomyocytes (SI 16-Fig. S16), suggesting that Phago-Muse cells did not acquire an epigenetic state similar to cardiomyocytes or that the ChIP-seq signal detected was not specific enough. In regard to the former issue, the insufficient overlap might be due to the difference of differentiation stage; iPS cell-derived cardiomyocytes were at more mature state (day 6 and day 32 after induction) than h-Muse cells (16 h after incubating with DDCs). In any event, these concerns require deeper verification in future research.

Although GATA-4-m-HL-1 cells (mouse) and h-Muse cells (human) are both mammalian cell lines, their transcription factors are not identical. *GATA-4* has 91% homology between humans and mice, while the DNA-binding sequence is identical. This might be why mouse GATA-4 was found to bind to GATA binding motifs in the human genome (Fig. 7E).

### Phagocytic receptors

Phagocytic activity is observed in normal healthy somatic cells and somatic stem cells such as MSCs and NSCs [15–21]. The observed phagocytosis receptors, however, differ from those of macrophages. Macrophages express a variety of receptors, including the TIM-4 family and MFG-E8, and the latter type plays a central role in inflammatory macrophages [47, 48]. On the other hand, CD36, ITGB3, CD91/LRP-1, and RAGE seem to be the main receptors in h-Muse cells because suppressing these receptors affected lineage-specific marker expression when incubated with DDCs. CD36 and SCARF1 were the main receptors after exposure to DDCs in h-MSCs. MERTK was reported to be the major receptor in the subventricular zone-NSCs [16], while our data suggested that CD36, CD91/LRP-1, MERTK, CD14, and ICAM are also active in r-NSCs. Although receptor subsets differ among Muse cells, MSCs, and NSCs, CD36 seemed to be commonly active in these cells.

The major phagocytic receptors in macrophages, such as TIMD4-1, -2, and -3, BAI1, CD300LF, STAB2, and ICAM-3 in h-Muse cells, were under the detection limits in both qPCR and Western blotting. Because somatic stem cells are not professional phagocytes, they expressed some but not all of the receptors expressed on macrophages, and thus, it is possible that the phagocytic machinery in not fully operational in somatic stem cells. These factors might explain why phagocytic activity of somatic stem cells was likely to be lower than that of macrophages.

Annexin V suppressed DDC-induced differentiation of Muse cells, MSCs, and NSCs. Treating DDCs with annexin V substantially reduced, but did not abolish, phagocytosis activity in h-Muse cells. Nevertheless, lineage-specific marker expression was almost completely suppressed (Fig. 6D, SI 12-Fig. S12). This might be because the amount of phagocytosis was substantially lower in each phagocytosis(+) Muse cell when the DDCs were pretreated with annexin V (Fig. 6A, B), making the amount of DDC-derived signals insufficient to drive lineage-specific differentiation. Notably, annexin V remarkably inhibited differentiation to the DDC cell type compared with the suppression of phagocytosis receptors using siRNA in h-Muse cells (Figs. 6H, S13F). This might be because annexin V broadly covers eat-me signals such PS and C1q [47, 48]. Because the majority of receptors expressed on Muse cells bind to PS either directly or indirectly, annexin V could generally block the signal input in Muse cells compared with siRNA. Moreover, the targeted suppression of individual receptors by siRNA might be compensated for by other types of receptors, thus limiting the effect of the suppression on differentiation.

Several published reports have discussed the mode of cellular uptake. Cellular cannibalism refers to a cell engulfing another living cell of its own or another type [60–66]. Emperipolesis is an unusual biological process in which a cell penetrates another living cell. Unlike in phagocytosis where the engulfed cell is killed by macrophage lysosomal enzymes, in emperipolesis, the cell exists as a viable cell within another cell and can exit at any time without any structural or functional abnormalities in either of them [60, 65]. Peripolesis is the attachment of a cell to another cell, which differs from emperipolesis [66]. Cannibalism, emperipolesis, or peripolesis are unlikely to be involved in the phenomenon we observed because the majority of experiments in this study were based on apoptotic cell fragments and not on whole living cells. Even when Muse cells were co-cultured with living cells (i.e., intact primary cultured neonatal m-cardiomyocytes), the phenomenon relevant to these mechanisms was not recognized as far as we observed nor were cardiac markers newly expressed in h-Muse cells (Fig. 1A). On the other hand, transendocytosis is a molecular process in which the cytotoxic T-lymphocyte-associated protein 4 (CTLA-4) ligands CD80 and CD86 are physically removed from the host cell by CTLA-4 expressing cells, internalized, and thus destroyed [64]. Transendocytosis might be involved because h-Muse cells show increased expression of CD80 and CD86 when co-cultured with cardiac-derived DDCs (Fig. S7).

### The meaning of phagocytosis in somatic stem cells

Phagocytosis is assumed to be involved in maintaining tissue homeostasis through clearing cell debris. The purpose of phagocytosis in somatic cells might be basically the same as that of macrophages, namely removing dead cells to avoid inflammation and maintain tissue homeostasis [15–21]. As shown in this study, however, phagocytosis may have a different purpose, namely determination of the differentiation direction, in certain kinds of somatic stem cells such as MSCs, NSCs and Muse cells. This might also contribute to tissue homeostasis, because somatic stem cells need proper differentiation control to replace damaged/dying cells in vivo. In this system, the machineries that were originally active in the dying differentiated cells direct somatic stem cells to differentiate into the same cell type, enabling the stem cells to replace dying cells with fewer errors. Notably, induced pluripotent stem cells (iPS) showed low phagocytic activity; 4.9 ± 1.5% at 5 h and 5.3 ± 0.9% at 20 h, respectively (SI 18-Fig. S18). Thus, the mechanism shown in this study is considered active mainly in somatic stem cells. Because iPS cells do not reside in the body, the underlying cellular mechanism might be different from that of somatic stem cells.

Stem cells are usually induced to differentiate into the target cell type by treatment with a series of cytokines. These cytokines bind to receptors on the cell surface, mediate the activation of specific signal transduction pathways, and induce the expression of genes characterizing differentiated cells [1]. Phagocytosis is an alternative strategy to control lineage-specific differentiation of somatic stem cells with a rapid time course. Muse cells take 4–6 weeks to differentiate into melanocytes and cardiomyocytes, and MSCs takes 3–4 weeks to differentiate into adipocytes and chondrocytes by cytokine induction [9, 10, 54]. The weeks-long differentiation time of cytokine induction can be decreased to days by phagocytosing ‘model cells’ (e.g., apoptotic differentiated cells), as phagocytosis may directly and efficiently transfer differentiation-directing factors that evoke a series of reactions to trigger differentiation into the target cell-lineage in the cytoplasm and nucleus.

The in vivo experiments presented in this study are not under physiological conditions but are caused by an injury or stress (e.g., bilateral common carotid artery occlusion). Whether the mechanisms demonstrated in this study also occur to guide normal differentiation and tissue homeostasis during development remains to be investigated in the future.

## Materials and methods

### Animals

C57BL/6 mice, C57BL/6-TG (CAG-EGFP) mice, and Sprague Dawley rats were used in this study. All animals were treated according to the regulations of the Standards for Human Care and Use of Laboratory Animals of Tohoku University. The animal experiments were approved by the Animal Care and Experimentation Committee of Tohoku University Graduate School of Medicine (permission No. 2019MdA-265-03).

### Preparation of h-Muse cells and -MSCs

Human bone marrow-mesenchymal stem cells (h-BM-MSCs) were purchased from Lonza (Basel, Switzerland, PT-2501). Cells were cultured at 37 °C, 5% CO_2_ in DMEM (Invitrogen, Carlsbad, CA, USA) containing 10% FBS (HyClone, Logan, UT, USA), 1 ng/ml basic fibroblast growth factor (bFGF) (Miltenyi Biotec, Bergisch Gladbach, Germany), 0.1 mg/ml kanamycin (Invitrogen), and 1% GlutaMAX (Invitrogen). Cells from passages 4 through 9 were used. For collecting h-Muse cells, BM-MSCs were incubated with anti-stage-specific embryonic antigen-3 antibody (1:1000; BioLegend, San Diego, CA, USA, 330302) followed by staining with a secondary antibody, fluorescein isothiocyanate-conjugated anti-rat IgM (1:100; Jackson ImmunoResearch Laboratories, Inc., West Grove, PA, USA, 112-095-075), as previously described [6]. SSEA-3(+) Muse cells and SSEA-3 (−) non-Muse cells, used as ‘h-MSCs,’ were collected by fluorescence-activated cell sorting (FACS; BD FACSAria™ II cell sorter, Becton Dickinson, San Jose, CA, USA).

H-Muse cells and h-MSCs stably expressing GFP, mCherry, GCaMP-3, *GATA-4*p-mCherry or *SOX9*p-mCherry were prepared as described previously [6, 13]. For lentivirus production, pMD2G, pCMV deltaR8.74, pWPXL-GFP, pWPXL-mCherry, pWPXL-GCaMP-3, pWPXL-*GATA-4*p-mCherry, or pWPXL-*SOX9*p-mCherry were transfected into LentiX-293T packaging cells (TaKaRa Bio Inc, Shiga, Japan) using Lipofectamine 2000 (Thermo Fisher Scientific, MA, USA). After 3 days, the viral supernatant was collected, centrifuged, and filtered through a 0.45-μm filter. Human BM-MSCs were infected with either GFP-, mCherry-, GCaMP-3-, *GATA-4*p-mCherry-, or *SOX9*p-mCherry-lentivirus, and then, SSEA-3(+) or SSEA-3(−) cells were collected as h-Muse cells or h-MSCs, respectively.

For sorting h-Muse cells or h-MSCs from GFP-, mCherry-, GCaMP-3-, *GATA-4*p-mCherry-, or *SOX9*p-mCherry-labeled human BM-MSCs, BM-MSCs were incubated with rat anti-SSEA-3 antibody (1:1000; BioLegend), followed by incubation with allophycocyanin-conjugated anti-rat IgM (1:100; Jackson ImmunoResearch, 112-136-075) for GFP-, GCaMP-3-h-Muse cells and fluorescein isothiocyanate-conjugated anti-rat IgM (1:100; Jackson ImmunoResearch) for mCherry- and *GATA-4*p-mCherry-h-Muse cells or *SOX9*p-mCherry-h-MSCs, and then sorted by the FACSAria™ II.

For in vivo time-lapse imaging, *NeuroD1*p-cyan fluorescent protein (CFP)-mCherry-h-Muse cells were prepared. Briefly, the EF1-α promoter and GFP in pWPXL were replaced by the h*NEUROD1* promoter from p*NEUROD*-ires-GFP (Addgene, 61403) and CFP from pECFP-Golgi (TaKaRa Bio USA), respectively. The mCherry-BM-MSCs were then transduced with lentiviruses produced according to the above-described protocol. Cells that were double-positive for both SSEA-3 and mCherry were collected before injection into a mouse focal stroke model.

### Preparation of rat neural stem cells

Rat NSCs were prepared from embryonic brains in the form of neurospheres, which are floating clonal aggregates formed by NSCs in vitro. In brief, single-cell suspensions prepared from the embryonic rat brains at embryonic day (E) 14.5 by trypsinization and mechanical trituration were cultured in Neurobasal medium (Invitrogen) with B27 supplement (Invitrogen), 20 ng/ml bFGF, and 20 ng/ml epidermal growth factor (EGF) (Peprotech, Rocky Hill, NJ). Neurospheres formed after 1 week were collected for passage or further analyses.

### Preparation of “differentiation-directing cells (DDCs)”

All primary cultures were prepared from animals killed by a lethal dose of isoflurane anesthesia. Mouse and rats were obtained from SLC (Hamamatsu, Japan). Cardiomyocytes, neural cells, liver cells, renal cells and intestinal cells were prepared by primary culture.

#### Mouse cardiomyocytes (m-cardiomyocytes)

Neonatal hearts isolated from 1-day old C57BL/6 mice were minced and digested for 10 min at 37 °C in 0.1% trypsin (Invitrogen) and collagenase/dispase (Roche, Mannheim, Germany) in phosphate-buffered saline (PBS). Cardiomyocytes were centrifuged 5 min at 500×*g*, and the supernatant was discarded. Cells were plated on laminin/collagen-coated dishes and maintained in DMEM and 15% FBS [67]. GFP-m-cardiomyocytes were collected from C57BL/6-TG (CAG-EGFP) mice in the same way.

#### Rat renal cells

Fetuses were obtained from a pregnant Sprague–Dawley rat (pregnant day 18) after administering a lethal dose of anesthesia. Isolated fetal kidneys were minced into 1-mm pieces and dispersed in 1 ml of digestion buffer comprising DMEM high-glucose (Gibco, 10,569,010), 10% FBS, 0.15% collagenase (Gibco, 17,100,017), and hyaluronidase (MilliporeSigma, St. Louis, MO, USA, H3506) at 37 °C for 30 min with gentle shaking. Cells were collected by centrifuging at 400×*g* for 5 min and cultured in 2 ml of culture medium comprising DMEM high-glucose, 20 mM HEPES, 5% FBS, and 0.2% Insulin (MilliporeSigma, l1882) at 3.5 × 10^5^ cells/35-mm dish overnight for the following apoptosis treatment [68].

#### Rat neural cells

Fetuses were obtained from a pregnant Sprague–Dawley rat (pregnant day 17) after administering a lethal dose of anesthesia. The hippocampus was isolated from each brain, dispersed in 1 ml of TrypLE Select (Gibco, 12,563,011) at 37 °C for 25 min with gentle shaking. Cells were collected by centrifuging at 400×*g* for 5 min and then, cultured on poly-d-lysine-coated dishes in neurobasal media plus B27 supplement at 3.5 × 10^5^ cells/35-mm dish for 3 days for the following apoptosis treatment [69].

#### Rat intestinal cells

Fetuses were obtained from a pregnant Sprague–Dawley rat (pregnant day 18) after administering a lethal dose of anesthesia. Isolated fetal intestines were minced into 1-mm pieces and dispersed in 1 ml of digestion buffer at 37 °C for 30 min with gentle shaking. Cells were collected by centrifuging at 400×*g* for 5 min and cultured in 2 ml of culture medium at 3.5 × 10^5^ cells/35-mm dish overnight for the following apoptosis treatment [70].

#### Rat hepatic cells

Fetuses were obtained from a pregnant Sprague–Dawley rat (pregnant day 18) after administering a lethal dose of anesthesia. Isolated fetal livers were minced into 1-mm pieces and dispersed in 1 ml of digestion buffer at 37 °C for 30 min with gentle shaking. Cells were collected by centrifuging at 200×*g* for 3 min after filtration using a 40-μm cell strainer. The pellet was suspended in 10 ml of DMEM and centrifuged at 50×*g* for 3 min. This procedure was repeated three times to wash the cells. Cells were then cultured in 2 ml of culture medium at 3.5 × 10^5^ cells/35-mm dish overnight for the following apoptosis treatment [71].

#### Mouse adipocytes

3T3-L1 cells were obtained from the Japanese Collection of Research Bioresources Cell Bank (JCRB, Osaka, Japan) and cultured in DMEM containing 10% FBS at 37 °C under 5% CO_2_. For adipocyte differentiation, 3T3-L1 cells were seeded to 4-well plate at a density of 1.1 × 10^4^ cells/cm^2^ and cultured for 24 h. Then, the cultures were induced by DMEM containing 10% FBS, 10 μg/ml insulin, 1 μM dexamethasone and 0.5 mM 1-methyl-3-isobutylxanthine. After 4 days, the medium was changed to 10% FBS-DMEM containing only 10 ug/ml insulin for an additional 3 days.

#### Mouse chondrocytes

ATDC5 cells were obtained from the RIKEN Cell Bank (Tsukuba, Japan). The cells were maintained in DMEM/F-12 medium supplemented with 10% FBS at 37 °C under 5% CO_2_. The cells were seeded to 4-well plate at a density of 1 × 10^5^ cells/well and cultured in the growth medium until confluent. To induce differentiation, confluent cells were treated with DMEM/F-12 containing 1% insulin-transferrin-selenium solution (Thermo Fisher Scientific) and 50 μg/ml l-ascorbic acid 2-phosphate. Differentiation was conducted for 7 days and medium was changed every 2 days.

#### Human neural cells

ReNcells, an immortalized human neural progenitor cell line, were obtained from MilliporeSigma. The cells were maintained in ReNcell NSC maintenance medium (MilliporeSigma) supplemented with 20 ng/ml bFGF and 20 ng/ml EGF at 37 °C under 5% CO_2_. The cells were seeded onto 4-well plates at a density of 2 × 10^4^ cells/well and cultured overnight. The next day, differentiation was induced by replacing the medium in each well with fresh ReNcell NSC Maintenance Medium containing no FGF-2 or EGF. Differentiation was conducted for 2 weeks, and the medium was changed every 2–3 days.

HL-1 (mouse cardiac muscle cell line, MilliporeSigma, Darmstadt, Germany), Hepa1-6 (mouse liver cell line, American Type Culture Collection), C2C12 (Mouse skeletal muscle cell line, American Type Culture Collection, Manassas, Virginia), rat keratinocytes (Cell Applications, San Diego, CA, USA), and rat alveolar cells (Creative Bioarray, Shirley, NY, USA) were obtained commercially. Gene-introduced cells were prepared as follows:

#### mCherry introduced m-Hepa1-6

For lentivirus production, pMD2G, pCMV deltaR8.74, or pWPXL-mCherry were transfected into LentiX-293 T packaging cells (TaKaRa) using Lipofectamine 2000 (Thermo Fisher Scientific). After 3 days, the viral supernatant was collected, centrifuged, and filtered through a 0.45-μm filter. M-Hepa1-6 cells were infected with mCherry-lentivirus and then expanded.

#### *GATA-4*-mCherry reporter m-HL-1

Robust generation of a *Gata-4*-mCherry knock-in m-HL-1 cell line was performed by using CRISPR-Cas9. CMV-Cas9N-2A-GFP (ATUM, Newark, CA, USA) was used as the nickase expression vector. Guide RNA was designed by UNITECH (Chiba, Japan). M-HL-1 cells were maintained in Claycomb medium (MilliporeSigma) with 10% FBS. M-HL-1 cells were transfected with CRISPR-Cas9 and gene-targeting plasmids using FuGENE6 transfection reagent following the manufacturer’s instructions. M-HL-1 clones that had stably integrated the targeting vector were identified by selection with 2 μg/ml puromycin. Drug-resistant clones were manually collected into 96-well plates and expanded for genomic DNA extraction and continued culturing. Targeting efficiency was quantified by PCR screening of genomic DNA from drug-resistant clones. Amplicon identity and gene targeting were confirmed by sequencing (UNITECH, Japan).

#### HA tag-GATA-4-m-HL-1 DDCs

For lentivirus production, pMD2G, pCMV deltaR8.74, or pWPXL- *GATA-4* promoter-HA tag-*GATA-4*-T2A-mCherry were transfected into LentiX-293 T packaging cells (TaKaRa) using Lipofectamine 2000 (Thermo Fisher Scientific). After 3 days, the viral supernatant was collected, centrifuged, and filtered through a 0.45-μm filter. M-HL-1 cells were infected with *GATA-4* promoter-HA tag-GATA-4-T2A-mCherry, and then, mCherry-positive cells were isolated by FACS.

### Collection of dead cell fragments

To generate apoptotic-m-cardiomyocytes, -Hepa1-6 and -GATA-4-mCherry reporter m-HL-1, 1 × 10^7^ cells were seeded onto 100-mm dishes; apoptosis was induced the next day with 50 μM etoposide (MilliporeSigma, E1382) in DMEM with 10% FBS for 24 h [72]. More than 95% of cells died and floated in the culture medium.

To induce apoptosis in renal cells, neural cells, intestinal cells, hepatic cells, alveolar cells, keratinocytes, adipocytes, chondrocytes, and human neural cells, 3.5 × 10^5^ cells were treated with 100 μM rotenone (MilliporeSigma, R8875) and 100 μM antimycin A (MilliporeSigma, A8674) to generate apoptotic DDCs [73]. More than 95% of cells died by apoptosis within 24 h. The DDCs were vortexed at 2000 rpm for 10 s (MS1 Minishaker, IKA Works, Staufen, Germany), filtered by a 40-μm cell strainer (BD Falcon, 352,340), collected by centrifugation at 800×*g* for 5 min, and stored in 1 ml of CELLBANKER 1 Plus (Juji-Field, Japan; Cat. No. BLC-1P) at − 80 °C until use.

Apoptotic cell fragments were collected and washed twice with PBS to remove etoposide, antimycin, or rotenone from the fragments. GFP-labeled h-Muse cells were incubated with apoptotic cell fragments for 3 days and stained with PE-labeled anti-annexin V, an apoptotic cell marker. Few GFP-h-Muse cells were positive for annexin V.

### Incubation of h-Muse cells, h-MSCs and r-NSCs with apoptotic DDCs

For co-culture of intact-m-cardiomyocytes and h-Muse cells, 2 × 10^4^ intact m-cardiomyocytes were resuspended in DMEM with 10% FBS and then added to 1 × 10^4^ h-Muse cells that were seeded onto 4-well plates. The cells were incubated and analyzed.

For apoptotic-m-cardiomyocytes, floating cell fragments were collected after treatment and washed twice with PBS as described above. The number of cells for apoptosis treatment was adjusted to 2 × 10^4^ cells, and collected fragments were washed with PBS for two times to wash out apoptosis-inducing reagents and then, added to 1 × 10^4^ h-Muse cells that were seeded onto 4-well plates to produce DDCs: h-Muse cell incubation ratios 2:1 in DMEM with 10% FBS. The samples were incubated with DDCs for the first 3 days, washed out, and then, the culture medium was changed every 3 days.

For rat renal cells, neural cells, intestinal cells, hepatic cells, alveolar cells, and keratinocytes, 1 × 10^4^ h-Muse cells (seeded onto 4-well plates with DMEM containing 10% FBS and 1 ng / μl basic fibroblast growth factor) were supplied with DDCs collected from 2.0 × 10^4^ apoptotic cells and incubated for 3 days. DDCs were washed out, and then, the culture medium was changed every 3 days to Neurobasal with B-27 supplement for neural cells, and DMEM with 5% FBS for the other cells.

For mouse adipocytes, 1 × 10^4^ h-MSCs (seeded onto 4-well plates with DMEM containing 10% FBS) were supplied with DDCs collected from 1.0 × 10^5^ apoptotic cells and were incubated for 3 days. DDCs were washed out, and then, the culture medium was changed every 3 days.

For mouse chondrocytes, 1 × 10^4^ h-MSCs (seeded onto 4-well plates with DMEM containing 10% FBS) were supplied with DDCs collected from 1.0 × 10^5^ apoptotic cells and were incubated for 3 days. DDCs were washed out, then the cells were cultured in suspension, and culture medium was changed every 3 days. For functional gene expression analysis, 1 × 10^4^ h-MSCs were supplied with DDCs collected from 1.0 × 10^5^ apoptotic mouse chondrocytes and incubated for 3 days. The DDCs were washed out, and then, the cells were cultured in suspension with a mouse articular cartilage slice for 7 days.

For human neural cells, 1 × 10^4^ rat NSCs (seeded onto 4-well plates with Neurobasal medium with B27supplement) were supplied with DDCs collected from 1.0 × 10^5^ apoptotic cells and incubated for 3 days. The DDCs were washed out, and the culture medium was changed every 3 days. For functional gene expression analysis, 1 × 10^4^ rat NSCs (seeded onto 4-well plates with Neurobasal medium with B27 supplement) were supplied with DDCs collected from 1.0 × 10^5^ apoptotic human neural cells and incubated for 3 days. Then, rat-NSCs were seeded onto the lower chamber of a Boyden chamber (BD Falcon) in Neurobasal medium with B27 supplement at a density of 5 × 10^4^ cells/well, and a damaged brain tissue slice was placed onto the upper chamber. The upper and lower chambers were separated by a porous septum. Damaged brain tissue was cut into ~ 2 mm-thick slices and placed into the Boyden chamber for co-culture with phagocytosed rat-NSCs for 7 days.

### Collection of conditioned medium

For intact-derived conditioned medium, neonatal m-cardiomyocytes (2 × 10^4^, 1 × 10^5^, or 1 × 10^6^ cells) were cultured in DMEM without FBS for 3 days. For apoptotic-m-cardiomyocytes conditioned medium, the number of cells for apoptosis treatment was adjusted to 2 × 10^4^, 1 × 10^5^, or 1 × 10^6^ and collected fragments were washed with PBS for two times to wash out etoposide, resuspended in DMEM without FBS, and cultured for 3 days. The conditioned medium was centrifuged at 400×*g* for 5 min, filtered through a 0.45-μm filter to eliminate cellular debris, and then added to h-Muse cells plated at 1 × 10^4^ cells/24-well plate.

### Collection of cell extract

Intact m-cardiomyocytes (2 × 10^4^, 1 × 10^5^, or 1 × 10^6^ cells) were trypsinized for collection, resuspended in 100 μl PBS, homogenized using a pestle at 4 °C, and sonicated and centrifuged at 10,000×*g* for 10 min. For apoptotic-m-cardiomyocytes extract, 2 × 10^4^, 1 × 10^5^, or 1 × 10^6^ cells were treated to induce apoptosis. Only floating cell fragments were collected, resuspended in 100 μl PBS, homogenized using a pestle at 4 °C, and sonicated and centrifuged at 10,000×*g* for 10 min. After centrifugation, the supernatant was collected and stored at − 80 °C until use. H-Muse cells (1 × 10^4^) were cultured overnight in DMEM with 10% FBS. The cells were treated with intact, or apoptotic cell extract.

### Enzyme-linked immunosorbent assay

Conditioned medium and cell extracts, collected from 1 × 10^6^ apoptotic m-cardiomyocytes, were incubated with antibodies to detect hepatocyte growth factor (HGF; Abcam, Cambridge, UK), transforming growth factor beta 1 (TGFβ-1; Abcam, ab119557), and cardiotrophin-1 (Thermo Fisher Scientific, EMCTF1), followed by incubation with horseradish peroxidase (HRP)-streptavidin and substrate, and then measured with a microplate reader at 450 nm according to the manufacturer’s instructions. We used DMEM without FBS and PBS as a control.

### Collection of extracellular vesicles

Intact m-HL-1 cells were cultured in Claycomb medium (MilliporeSigma) without FBS. After 3 days, conditioned medium was centrifuged at 10,000×*g* for 30 min and passed through a 0.22-μm filter to remove cell debris and large fragments. Finally, the extracellular vesicles were pelleted by ultracentrifugation at 100,000×*g* for 70 min and then resuspended in PBS for use.

For collection of apoptotic extracellular vesicles, apoptosis was induced as described above. Next, m-HL-1 cells were cultured in Claycomb medium without FBS. After 3 days, conditioned medium was centrifuged at 10,000×*g* for 30 min and passed through a 0.22-μm filter to remove cell debris and large fragments. Finally, extracellular vesicles were pelleted by ultracentrifugation at 100,000×*g* for 70 min、 resuspended in PBS, and centrifuged again at 100,000×*g*, and then resuspended in PBS for use. Tunable resistive pulse sensing analysis was performed to analyze the extracellular vesicles using the qNano system (Izon, Christchurch, New Zealand). The protein amount in each sample was measured by using a BCA protein assay kit. Evaluation of the extracellular vesicles by TEM was performed as previously described [74].

For labeling extracellular vesicles, 100 μg of intact extracellular vesicles was labeled with PKH67 Green Fluorescent Cell Linker Kit (MilliporeSigma) according to the manufacturer’s instructions. H-Muse cells (2 × 10^4^) were treated with 100 μg of PKH67-labeled-intact extracellular vesicles. Three days later, h-Muse cells were fixed in 4% paraformaldehyde (PFA) in 0.1 M phosphate buffer saline, and counterstained with DAPI. The cells were inspected under a laser confocal microscope (Nikon). To analyze the effect of intact-, and apoptotic DDC-derived extracellular vesicles on h-Muse cell differentiation, 2 × 10^4^ h-Muse cells were treated with 50 μg/ml and 200 ng/ml extracellular vesicles.

### Detection of micro-RNA from extracellular vesicles

The total exosome RNA was extracted using a Total Exosome RNA and Protein Isolation Kit (Invitrogen) according to the manufacturer’s recommendations: exosome pellets were resuspended in PBS and denatured, and RNA was purified by acid-phenol/chloroform extraction.

Total RNA extracted from exosomes was reverse-transcribed to cDNA using a TaqMan MicroRNA Reverse Transcription Kit (Thermo Fisher Scientific) according to the manufacturer’s protocol. qPCR was performed at a volume of 20 μl containing 10 μl TaqMan Universal master mix, 1 μl TaqMan miRNA Assay, and 9 μl cDNA + RNase free water. The reactions were incubated at 95 °C for 10 min and then amplified 40 cycles, with each cycle comprising an incubation step at 95 °C for 15 s followed by 60 °C for 60 s. Small nuclear RNA U6 was used as an internal reference. The TaqMan gene expression assay was performed using the following primers: U6 snRNA, TM:001973; has-miR-208, TM:000511; has-miR-133a, TM:002246; has-miR-499, TM:001352; has-miR-206, TM:000510; and has-miR-1, TM:002222.

### Single-cell RNA sequencing

mCherry- or GFP-h-Muse cells (1 × 10^5^) were incubated for 3 days with m-cardiomyocyte DDCs, r-neural cell DDCs, and r-hepatic cell DDCs derived from 2 × 10^5^ cells to make Phago-cardio-, Phago-neuro-, and Phago-hepa-Muse cells, respectively, then washed out and cultured in medium (DMEM + 10% FBS) for 4 days. Next, mCherry- or GFP-h-Muse cells were sorted using the FACSAria™ II. For the control, naïve Muse cells were sorted from BM-MSCs as described above. The AC16 cell line, immortalized adult human cardiomyocytes (Millipore), was used for authentic human cardiomyocytes (Authen-Cardio). Cells for all samples were stained with barcode tag-conjugated β2-microglobulin antibody and CD298 antibody (BioLegend), counted and multiplexed, and prepared for single-cell capture. Single-cell capture and cDNA synthesis were performed by the BD Rhapsody Single-Cell Analysis System according to the manufacturer’s instructions. All libraries were sequenced using Novaseq 6000 to a read depth of at least 100,000 reads/cell. The deep sequencing and initial analysis were performed using ImmunoGeneTeqs (Tokyo, Japan) [32]. This study was analyzed with 1 biological replicate per group.

For scRNA-seq data analysis, Seurat R package v3 [75] was used for filtering, normalization, dimensionality reduction analysis, DEG analysis, and cell-cycle analysis. Doublets or cells with low quality (genes > 1500, genes < 10,000, or < 10% genes mapping to mitochondrial genome) were removed. Genes that were detected in fewer than three cells were removed. Normalization was performed by SCtransform [76] based on the number of unique molecular identifiers and the percentage of mitochondrial genes. Unsupervised clustering was performed by the FindClusters function with the parameters “Dim = 30, Res = 0.85”. To visualize cell-to-cell variations, the top 30 principal components were applied to the t-SNE algorithm [77]. We used the Wilcoxon rank-sum test to identify DEGs among each Phago-group and Naïve-Muse group. Genes with a fold-change greater than 1.5 or smaller than 2/3 times and a *p* value 0.05 or less were considered to be upregulated or downregulated, respectively. The CellCycleScoring function was used to determine a cell cycle score for each cell according to its gene expression of G2/M and S phase markers [78]. The cell cycle phase was decided on the basis of this score. Cluster 3.0 [79] and JAVA Treeview [80] were used to perform hierarchical clustering with correlation distance and complete linkage. Monocle2 R package [81, 82] was used for trajectory analysis and DEG analysis according to pseudo-timeline. The Database for Annotation, Visualization, and Integrated Discovery (DAVID: http://david.abcc.ncifcrf.gov) [83, 84] was used for the GO analysis. The gene expression of Tissue and Cell Type in the human brain, liver, and muscle tissue (heart and skeletal muscle) was acquired from The Human Protein Atlas (http://www.proteinatlas.org) [33]. For pseudotemporal depiction of the heatmap, The Human Protein Atlas [33], and BigGPS (http://biogps.org/#goto=welcome) [45] were referred to for selecting lineage-specific markers. Fetal transcriptome data were downloaded from the NCBI Gene Expression Omnibus (GSE157329) [85]. “Hepatocyte” and “hepatic stellate cell” were extracted as “Fetal (Liver cell)” from the data; “ventricle cardiomyocyte” as “Fetal (Cardiomyocyte)”; “GABAergic neuron,” “MNs,” “MNv,” “Schwann progenitor,” and “sympathetic neuron” as Fetal (Neuronal cell). Fetal data were normalized by SC transform and exported as corrected UMI count. A total of 200 cells was randomly selected from the fetal cells and each cluster, and genes that were detected in more than 2 UMI in 100 cells were applied for hierarchical clustering and heatmap visualization. Hierarchical clustering was performed using a simple linkage method.

### Next-generation sequencing (NGS)

For next-generation RNA sequencing, DDCs (with or without annexin V treatment) collected from 1.0 × 10^5^ apoptotic mouse adipocytes were supplied to 1 × 10^4^ h-MSCs (seeded in 4-well plates in DMEM containing 10% FBS) and incubated for 3 days. After washing the DDCs, total RNA was extracted after an additional 4 days of culture. Also, DDCs (with and without annexin V treatment) collected from 1.0 × 10^5^ apoptotic human neural cells were supplied to 1 × 10^4^ h-MSCs (seeded in 4-well plates in DMEM containing 10% FBS) and incubated for 3 days. After washing the DDCs, total RNA was extracted after an additional 4 days culture. RNA sequence library preparation, sequencing, mapping, and gene expression were performed by DNAFORM (Yokohama, Kanagawa, Japan). Qualities of total RNA were assessed with a Bioanalyzer (Agilent) to ensure an RNA integrity number > 7.0. After poly (A) + RNA enrichment by NEBNext Poly(A) mRNA Magnetic Isolation Module (New England BioLabs), double-stranded cDNA libraries (RNA-seq libraries) were prepared using a SMARTer stranded Total RNA sample Prep Kit – HI Mammalian (Clontech), and MGIEasy Universal Library Conversion Kit (App-A) (MGI) according to the manufacturer’s instructions. RNA-seq libraries were sequenced using paired end reads (50nt of read1 and 25nt of read2) on a NextSeq 500 instrument (Illumina). Obtained raw reads were trimmed and quality-filtered using the Trim Galore! (version 0.4.4), Trimmomatic (version 0.36), and cutadapt (version 1.16) software. Trimmed reads were then mapped to the human GRCh38 genome using STAR (version 2.7.2b). Reads on annotated genes were counted using featureCounts (version 1.6.1). FPKM values were calculated from mapped reads by normalizing to total counts and transcript. Differentially expressed genes were detected using the DESeq2 package (version 1.20.0). The list of differentially expressed genes detected by DESeq2 (basemean > 5 and fold-change < 0.25, or basemean > 5 and fold-change > 4) were used for GO enrichment analysis by clusterProfiler package [86]. The following software and tools were used for RNA-seq data analysis: Heatmapper, http://heatmapper.ca/expression/.

### Quantitative PCR

Total RNA was collected using the NucleoSpin^®^ RNA XS (Macherey–Nagel, Duren, Germany), and cDNA was synthesized using Oligo(dT)_20_ primers (Invitrogen) and SuperScript^®^ III reverse transcriptase (Invitrogen). DNA was amplified with the Applied Biosystems 7500 Fast real-time PCR system according to the manufacturer’s instructions. To evaluate the differentiation of h-Muse cells, we used quantitative reverse transcriptase PCR (q-PCR) assays with confirmed for species-specific primers. Cardiac markers were performed using TaqMan gene expression assay with the following primers: *NKX2.5*: Hs00231763_m1, *GATA-4*: Hs00171403_m1, A-type natriuretic peptide *(ANP*): Hs00383231_m1, *TnT*: Hs00943911_m1, *MLC2a*: Hs01085598_g1; *MLC2v*: Hs00166405_m1, *HCN2*: Hs00606903_m1; *CACNA1A*: Hs01579431_m1; *CACNA1C*: Hs00167681_m1; *CACNA1G*: Hs00367969_m1; *KCNJ2*: Hs01876357_s1, and *beta actin*: Hs01060665_g1.

For human specific-hepatic and -skeletal muscle markers, the following primers were used: *PROX1*: Hs00896293_m1; *KRT18*: Hs02827483_g1; *AFP*: Hs01040598_m1; *ALB*: Hs00609410_m1; *MYOD*: Hs0000159528_m1; *MYOGENIN*: Hs00231167_m1; and *PAX7*: Hs00242962_m1.

For evaluation of triploblastic lineage cells, the following primers were used: *FOXP2*: Hs00362818_m1; *PAX6*: Hs01088114_m1; *MATH1*: Hs00245453_s1; *NEUN*: Hs01370653_m1; *SCN2A*: Hs01109871_m1; *KCNB1*: Hs00270657_m1; *PSD95*: Hs01555373_m1; *Synaptophysin*: Hs00300531_m1: *MBP*: Hs00921945_m1; *LGR5*: Hs00969420_m1; *ATOH1*: Hs00245453_s1; *LYZ*: Hs00426232_m1; *FABP2*: Hs01573162_m1; *GFI1*: Hs00382207_m1; *WT1*: Hs01103754_m1; *EYA1*: Hs00166804_m1; *AQP1*: Hs00166067_m1; *AQP5*: Hs00893081_m1; *SCNN1A*: Hs00168906_m1; *PDPN*: Hs00366766_m1; *CDH1*: Hs01023895_m1; *KRT10*: Hs01043110_g1; *P63*: Hs00978341_m1; *KRT15*: Hs00267035_m1; and *DSG3*: Hs00951897_m1.

For human specific-adipocytes and –chondrocytes markers, the following primers were used: *CEBPB*: Hs00942496_s1; *PPARG*: Hs01115513_m1; *FABP4*: Hs01086177_m1; *SOX9*: Hs01107818_m1; *MMP13*: Hs00233992_m1; *COL10A1*: Hs00166657_m1; *ACAN*: Hs00153936_m1; *THBS4*: Hs00170261_m1; *SIX1*: Hs00195590_m1; *COL2A1*: Hs00264051_m1.

For rat-specific neural cells markers, the following primers were used: *NeuroD1*: Rn00824571_s1; *Neurofilament*: Rn00566763_m1; *Olig2*: Rn01767116_m1; *Sox10*: Rn00569909_m1; *Gfap*: Rn00566603_m1; *Vimentin*: Rn00579738_m1; *Hcn2*: Rn01408572_mH; *Kcnb1*: Rn00755102_m1; *Scn2a*: Rn00680558_m1; *Psd95*: Rn00571479_m1; *Synaptophysin*: Rn01528256_m1: *Mbp*: Rn01399619_m1; *Actb*: Rn00667896_m1.

For human specific-cardiac, -neural, and -hepatic markers, primers below were used for SYBR Green expression assay:

*EGLN1*-F: 5′-GTTTTTCTGGTCTGACCGTCG-3′

*EGLN1*-R: 5′-CTCGTGCTCTCTCATCTGCAT-3′

*SVIL*-F: 5′-GGAAGAGCCGGGTAGATTCTG-3′

*SVIL*-R: 5′-CTTCATGGGCTTCTCCTTCAGT-3′

*SLC16A7*-F: 5′-GACACGTCAGGGGCCATAAA-3′

*SLC16A7*-R: 5′-GCTGTCAACGGGTGAATGGA-3′

*EPHA4*-F: 5′-ACGCGTGCTCATCTTGTGTA-3′

*EPHA4*-R: 5′-GCAAACTCAGAGCCGAGCTA-3′

*ETV1*-F: 5′-CAGTGTCCCCACTGCATCAT-3′

*ETV1*-R: 5′-TGGGGTAGCTGCTATCTGGT-3′

*AKR1C1*-F: 5′-TCATGGTGGGGACATGATAGC-3′

*AKR1C1*-R: 5′-AAAGGCAGCGAAGGATTCAGA-3′

*SERPINA3*-F: 5′-ACTCCAGACAGACGGCTTTG-3′

*SERPINA3*-R: 5′-TCCATTCTCAACTCTGCCTCAG-3′

*CEACAM1*-F: 5′-GAACCTGACCTGCTCCACAA-3′

*CEACAM1*-R: 5′-GGACAGCTTCATCCTCTCCG-3′

Macrophage marker expression was evaluated using the GeneQuery Human Macrophage Polarization Markers qPCR Array Kit (ScienCell Research Laboratories, Carlsbad, CA, USA) according to manufacturer’s protocol. The kit included: C–C motif chemokine ligand 2 *(CCL2),* Cluster of differentiation 163 *(CD163),* Cell surface transmembrane glycoprotein 200 receptor 1 *(CD200R1),* Cluster of differentiation 68 *(CD68),* Cluster of differentiation 80 *(CD80),* Cluster of differentiation 86 *(CD86),* Hypoxia-induced factor 1-α *(HIF1A),* Major histocompatibility complex, class II, DQ alpha 1 *(HLA-DQA1),* Major histocompatibility complex, class II, DQ beta 1 *(HLA-DQB1),* HLA class II histocompatibility antigen, DR alpha chain *(HLA-DRA),* HLA class II histocompatibility antigen, DRB1 beta chain *(HLA-DRB1),* Interferon gamma *(IFNG),* Interleukin 10 (IL-10), Interleukin 12 A *(IL-12A),* Interleukin 1 beta *(IL1B),* Interleukin 1receptor, type I *(IL1R1),* Interleukin 1 receptor, type II *(IL1R2),* Interleukin 23 subunit alpha *(IL23A),* Interleukin 6 *(IL-6),* Interleukin regulatory factor 4 *(IRF4),* Kruppel-like factor 4 *(KLF4),* Mannose receptor C-type 1 *(MRC1),* Nuclear factor kappa B subunit 1 *(NFKB1),* Nitric oxide synthase 2 *(NOS2),* Platelet endothelial cell adhesion molecule 1 *(PECAM1),* Peroxisome proliferator-activated receptor gamma *(PPARG),* Suppressor of cytokine signaling 3 *(SOCS3),* Signal transducers and activators of transcription 1 *(STAT1),* Signal transducers and activators of transcription 3 *(STAT3),* Signal transducers and activators of transcription 6 *(STAT6),* Transforming growth factor β 1 *(TGFB1),* Toll like receptor 1 *(TLR1),* Toll like receptor 2 *(TLR2),* Toll like receptor 4 *(TLR4),* Toll like receptor 8 *(TLR8),* Tumor necrosis factor *(TNF),* Vascular endothelial growth factor A *(VEGFA)*.

For phagocytosis receptors in h-Muse cells, the following primers were used: *STAB2*: Hs01000844_m1; *CD36*: Hs00354519_m1; *MEGF10*: Hs00260998_m1; *CD300LF*: Hs01090530_m1; *ITGB3*: Hs01001469_m1; *CD14*: Hs02621496_s1; *SCARF1*: Hs01092477_m1; *CD91/LRP-1*: Hs00233856_m1; *MERTK*: Hs01031979_m1; *BAI1*: Hs00181777_m1; *RAGE*: Hs00542584_g1; *TIMD4-1*: Hs00293316_m1; *TIMD4-2*: Hs00383726_m1; *TIMD4-3*: Hs0038722_m1; *ICAM1*: Hs00164932_m1; *ICAM3*: Hs00913466_g1; and *LFA-1/CD11A*: Hs00158218_m1.

Positive controls were prepared from the following samples: human-specific cardiomyocyte markers: human fetal heart total RNA (TaKaRa); human-specific neural, hepatic, renal cells, and keratinocytes: human fetus, whole poly A^+^ RNA (TaKaRa); human-specific intestinal cells: human small intestine total RNA (TaKaRa); and human-specific alveolar cells: human fetal lung poly A^+^ RNA (TaKaRa).

### Western blotting

For confirmation of the extracellular vesicle recovery, the expression of HSP70, CD63, and TSG101 was examined [87, 88]. The protein amount in each sample was measured using a bicinchoninic acid (BCA) protein assay kit (Thermo Fisher Scientific) according to the manufacturer’s instructions, and samples containing 5 μg protein were loaded onto the gel. The anti-HSP70 antibody (1:1000, ENZO Life Sciences, ADI-SPA-810, Farmingdale, NY, USA), anti-CD63 antibody (1:1000, GeneTex, GTX17441, Taiwan), and anti-TSG101 antibody (1:1000, Abcam, ab83) were used. The primary antibody was detected with HRP-conjugated anti-rabbit IgG antibody. A C57/B6 mouse kidney homogenized sample was used as the positive control, as described previously [88–90].

For phagocytic receptors, protein amounts of naïve h-Muse cells, hMSCs, and rat NSCs at D2 after exposure to DDCs were measured using a BCA protein assay kit and samples containing 5 μg protein were loaded onto the gel. The anti-SCARF1 antibody (1:1000, Abcam, ab92308), anti-CD91/LRP-1 antibody (1:1000, Abcam, ab92544), anti-MEGF10 antibody (1:1000, LSBio, LS-C668447, Seattle, WA, USA), anti-LFA-1 antibody (1:1000, Abcam, ab89725), anti-ICAM-1 antibody (1:1000, Abcam, ab2213), anti-ITGB3 antibody (1:1000, Santa Cruz Biotechnology, sc-365679), anti-RAGE antibody (1:1000, Santa Cruz Biotechnology, sc-80652), anti-MerTk antibody (1:1000, Abcam, ab52968 (for human)), anti-MerTk antibody (1:1000, SantaCruz, sc-365499 (for rat))anti-CD14 (1:1000, Abcam, ab45870), anti-CD36 (1:1000, Abcam, ab133625), anti-TIMD4 antibody (1:1000, R&D Systems, AF2929, Minneapolis, MN, USA), anti-BAI1 antibody (1:1000, LSBio, LS-C177193), anti-CD300LF antibody (1:1000, LSBio, LS-C168845), anti-STAB2 antibody (1:1000, Santa Cruz Biotechnology, sc-518006), and anti-ICAM3 antibody (1:1000, Abcam, ab212469) were used for Western blotting. The primary antibody was detected with HRP-conjugated anti-rabbit, goat, and mouse IgG antibody.

### Immunocytochemistry

Cells were fixed with 4% PFA in 0.1 M PBS, as described previously [13]. The following primary antibodies were used for cardiac cell markers: GATA-4 (1:500; Santa Cruz Biotechnology, sc-1237), ANP (1:500; Santa Cruz Biotechnology, sc-18811), and TNT (1:100; Millipore, MAB1691); for renal cell markers: WT1 (1:100; Santa Cruz Biotechnology, sc-192) and MEGSIN (1:100; Bioss Inc, Woburn, MA, USA, bs-0815R); for neural cell markers: NESTIN (1:200; Millipore, MAB5326), TUBULIN β3 (1:5000; BioLegend, MRB435P), and NEUROFILAMENT M (1:200; Millipore, AB1987); for keratinocyte markers: CYTOLERATIN 5 (1:100; Abcam, ab75869) and CYTOLERATIN 14 (1:100; Abcam, ab51054); for intestinal cell markers: LGR5 (1:100; Acris Antibodies, AP12376PU-N), ATOH1 (1:200; Millipore, AB5692), ELF3 (1:100; Thermo Fisher Scientific, PA5-47776), and FABP2 (1:200; Novus Biologicals, Centennial, CO, USA; NBP1-51589); for hepatic cell markers: PROX1 (1:50; R&D Systems, AF2727), SOX9 (1:1000; Millipore, AB5535), ALBUMIN (1:50; Bethyl, Montgomery County, TX, USA, A80-229A) and CYTOKERATIN 7 (1:100; DAKO, M7018); for alveolar cell markers: surfactant protein C (SFTPC; 1:25; Thermo Fisher Scientific, PA5-71680), AQP5 (1:100; Abcam, ab92320); for adipocytes marker: FABP4 (1:50; R&D systems, 967799); and for chondrocytes marker: AGGRECAN (ACAN; 1:50; R&D systems, 967800). These primary antibodies were detected with either Alexa568-conjugated donkey anti-goat IgG (1:500: Thermo Fisher Scientific, A11057), anti-rabbit IgG (1:500: ThermoFisher Scientific, A10042) or anti-mouse IgG antibody (1:500: Thermo Fisher Scientific, A10037). The cells were then stained with 4′,6-diamidino-2-phenylindole (DAPI). The samples were observed under a confocal laser microscope (A1; Nikon, Tokyo, Japan).

To assess the specificity of the primary antibody, a positive control was used as follows: tissue sections of rat heart tissue were used as a positive control for GATA-4, ANP, and TNT; mouse kidney for WT1 and MEGSIN; mouse brain for NEUROFILAMENT, and TUJ1; mouse skin for CYTOKERATIN 5 and CYTOKERATIN 14; mouse intestine for LGR5, ATOH1, ELF3, and FAPB2; and mouse lung for AQP5 and SFTPC. NTERA-2 cl. D1 (ATCC-CRL-1973) was used as a positive control for nestin; HepG2 (ATCC, HB-8065) cells were used as a positive control for PROX1, SOX9, and albumin; and HeLa (ATCC, CCL-2) were used as a positive control for cytokeratin 7. NTERA-2 cl. D1 were maintained in DMEM high-glucose supplemented with 10% FBS, 0.1 mg/ml kanamycin. HepG2 and HeLa were cultured in DMEM low-glucose containing 10% FBS, 0.1 mg/ml kanamycin, and 1% GlutaMAX.

For positive control of adipocytes and chondrocytes, human-MSCs were induced into adipocytes and chondrocytes by Human Mesenchymal Stem Cell Functional Identification Kit (R&D Systems, SC006), according to the manufacturer’s instructions.

Sections were stained with only the secondary antibodies and then stained with DAPI as a nuclear counterstain for the negative control. Anti-rabbit IgG antibody conjugated to Alexa Fluor 568 was used for WT-1, MEGSIN, NEUROFILAMENT, TUJ1, CYTOKERATIN 5, CYTOKERATIN 14, LGR5, ATOH1, SOX9, AQUAPORIN 5, and SFTPC. Anti-goat IgG antibody conjugated to Alexa Fluor-568 was used for GATA-4, ANP, ELF3, PROX1, and ALBUMIN. Anti-mouse IgG antibody conjugated to Alexa Fluor-568 was used for TNT, NESTIN, FABP2, and CYTOKERATIN 7. Anti-goat IgG antibody conjugated to Alexa Fluor-488 was used for FABP4 and ACAN.

### Intracellular Ca^2+^ imaging

The intracellular Ca^2+^ imaging was recorded by using GCaMP-3-h-Muse cells, the popular genetically encoded Ca^2+^ indicator. For cardiac or neural differentiation, 1 × 10^4^ GCaMP-3-h-Muse cells were incubated with DDCs derived from 2 × 10^4^ apoptotic m-cardiomyocytes or rat neural cells for 3 days. Then, GCaMP-3-h-Muse cells were seeded onto the lower chamber of a Boyden chamber (BD falcon) in DMEM with 10% FBS at the density of 5 × 10^4^ cells/well, and damaged heart or brain tissue slice was placed onto the upper chamber. The upper and lower chambers were separated by pore septum. The mouse damaged heart was induced by intravenous injection of doxorubicin, and the rat damaged brain was prepared by BCCAO in order to make global cerebral ischemia, according to the previously described methods [91, 92]. Damaged heart and brain were cut into ~ 2-mm-thick slices and were placed into the Boyden chamber for co-culture with phagocytosed GCaMP-3-h-Muse cells. After 1 week, GCaMP-3-h-Muse cells were re-plated onto 13 mm glass-bottom dish at 2 × 10^4^ cells and subjected to live Ca^2+^ imaging following 1 day incubation. For the remaining cells, total RNA was collected and gene expression analysis was performed by qPCR. Cells were stimulated by 50 mM KCl for neural cells [93] or 70 mM KCl for cardiomyocytes [94] in FluoroBrite DMEM, and GFP fluorescence excited by 950 nm IR laser was recorded by using a Nikon A1 MP multiphoton laser scanning microscope.

### Immunohistochemistry

Cryosections (6-µm thick) were treated with Tris–EDTA (pH 9.0) for antigen retrieval for 20 min at 80 °C, rinsed with PBS, and incubated with blocking solution containing 5% bovine serum albumin for 30 min at room temperature, as previously reported [13]. The sections were incubated with anti-LAMP-1 (1:1000; Abcam, ab24170) at 4 °C overnight. After overnight incubation with the primary antibodies, the sections were rinsed with PBS and incubated with Alexa 568-conjugated anti-rabbit IgG antibody (1:1000; Thermo Fisher Scientific, A10042). The sections were then stained with DAPI and washed three times with PBS. Samples were mounted and observed under a confocal laser microscope (Nikon).

For evaluating phagocytic-receptor suppressed 4-siRNA-h-Muse cell differentiation in the post-infarct stroke brain, cryosections were stained with primary antibodies of GFP (1:1000, ab13970, Abcam) and NEUN (1:500; ab177487, Abcam), followed by secondary antibody incubation with anti-mouse and rabbit IgG conjugated with Alexa Fluor-594, and inspected under laser confocal microscope (Nikon). Ten randomly taken images were subjected to analysis.

To examine adipogenesis, the cells were fixed with 4% PFA in PBS for 30 min, rinsed with water followed by 70% ethanol, and stained with Oil Red O solution (MUTO PURE CHEMICALS, Tokyo, 40491) for 30 min. Excess stain was removed by washing once with 70% ethanol, and three times with water.

In Alcian Blue staining, the sections were stained with hematoxylin for 15 min prior to being stained with 0.5% Alcian Blue (MUTO PURE CHEMICALS, 19861) for 10 min.

### Fluorescence in situ hybridization analysis

All procedures were performed as described previously [13]. H-Muse cells (1 × 10^5^) incubated for 3 days with either 1 × 10^5^ intact and 2 × 10^5^ apoptotic-m-Hepa1-6 DDCs were harvested and treated with 0.075 M KCl for 20 min at room temperature. They were fixed with Carnoy’s solution for 5 min at 4 °C, then rinsed in Carnoy’s solution, and spread onto glass slides. After drying for 3 h at 37 °C, the slides were heated at 70 °C and treated with 70% formamide/2 × saline sodium citrate (SSC) for 2 min. The cells were dehydrated in 70%, 90%, and 100% ethanol for 2 min each at room temperature and air dried. A mixture of human genomic DNA-specific probe (SPH-20, Chromosome Science Labo Inc., Sapporo, Japan) labeled with Green-dUTP and mouse genomic DNA-specific probe (SPM-20, Chromosome Science Labo Inc.) labeled with Red-dUTP was denatured for 10 min at 75 °C and placed onto the slides. The sections were covered with coverslips and hybridized in a humid chamber overnight at 37 °C. The sections were then washed with 2 × SSC for 5 min at 37 °C and the coverslips carefully removed. The sections were washed with preheated 50% formamide/2 × SSC for 20 min at 37 °C, with 1 × SSC for 15 min at room temperature, and then counterstained with DAPI. The samples were analyzed under a laser confocal microscope (A1, Nikon), and more than 4500 cells were counted.

### In vitro live image observations

Incubation of (1) GFP-h-Muse cells and apopototic-mCherry-m-Hepa1 DDCs and (2) GFP-h-Muse cells and apoptotic-GATA-4-mCherry reporter m-HL-1 DDCs, both with and without annexin V were recorded under a laser confocal microscope (Nikon) at 37 °C, 5% CO_2_ for live image analysis. The cells were counterstained with Hoechst 33,342 (Thermo Fisher Scientific, H3570). Images were captured every 15 min with a Z-series for three-dimensional imaging. BITPLANE IMARIS system (Zeiss, Jena, Germany) was used to analyze the obtained live image data.

For *GATA-4*p-mCherry expression in h-Muse cells after phagocytosis of apoptotic-GFP-m-cardiomyocytes, h-Muse cells (plated at 2 × 10^4^ cells) were incubated with etoposide-treated apoptotic-GFP-m-cardiomyocyte DDCs generated from 4 × 10^4^ cells and were recorded by the IncuCyte live cell imaging system (Essen Bioscience, Hertfordshire, UK) and laser confocal microscopy.

For *SOX9*p-mCherry expression in h-MSCs after phagocytosis of apoptotic-GFP-m-chondrocytes, h-MSCs (plated at 2 × 10^4^ cells) were incubated with 100 μM rotenone-treated apoptotic-GFP-m-chondrocyte DDCs generated from 4 × 10^4^ cells and were recorded by laser confocal microscopy.

### Generating animal models and in vivo imaging

A rat acute myocardial infarction model was generated as described previously [95]. The left coronary artery of deeply anesthetized male Sprague Dawley rats (body weight 250–300 g) was ligated, and 2 × 10^4^ GFP-h-Muse cells were injected via the tail vein 24 h after the ligation. Five days after the h-Muse cell injection, the rats were deeply anesthetized and the heart was removed and fixed with periodate lysine paraformaldehyde solution (0.01 M NaIO_4_, 0.075 M lysine, 2% PFA, pH 6.2). Cryosections were cut and immunostained for LAMP-1 as described above.

A mouse stroke model was generated according to the previously described method [12]. Briefly, 12-week-old C57BL/6-Tg (CAG-EGFP) mice (Japan SLC, Inc., Shizuoka, Japan) were anesthetized with 2.5% isoflurane using a face mask, and rectal temperature was maintained at 37 °C using a self-regulating heating pad during the operation. A hole (φ2 mm) was drilled in the parietal bone, and 2 μl of Endothelin 1 (ET-1) (E7764, MilliporeSigma, dissolved in sterile saline at 1 mg/ml) was injected into the parietal cortex using a microliter syringe (Hamilton Company) at a flow rate of 2 μl/min. After 24 h, 1.0 × 10^5^ *NEUROD1*p-CFP-mCherry-h-Muse cells were suspended in 3 μl sterile saline and injected into the same spot using a microliter syringe at a flow rate of 3 μl/min. Part of the parietal bone around the injection site was replaced with a thin plastic cover made from polyvinyl chloride using a drill and tweezers. Time-lapse imaging was started using a Nikon A1 MP multiphoton laser scanning microscope (Nikon) with CFI75 LWD 16X objective lens (Nikon) 6 h after the cell injection. The time-lapse images were analyzed and edited using NIS-Elements software (ver. 4.13, Nikon). For visualization of the infarct site, triphenyltetrazolium chloride (TTC) staining was performed at 1 day after the onset of ischemia [96]. To confirm the expression of CFP in injected mCherry-h-Muse cells, mice were perfused with 4% PFA in PBS one day after receiving cell injection and cryosections were made. Samples were mounted and observed under a confocal laser microscope (A1, Nikon).

For evaluating phagocytosis receptor suppressed 4-siRNA-h-Muse cell differentiation in vivo, 120 min BCCAO model was made in 8-week-old male C57BL/6 N under deep anesthesia. Then, 5 × 10^4^ naïve GFP-h-Muse cells or 4-siRNA-h-Muse cells were injected stereotaxically into the ischemic cortex (from the bregma: anterior–posterior, − 2.0 mm; medial–lateral, − 2.0 mm; dorsal–ventral (from the dural surface), − 3.0 mm) at 2 day after onset. One week after the cell injection, animals were perfused with 4% paraformaldehyde in PBS and subjected to immunohistochemistry.

### TEM

GFP-h-Muse cells were incubated for 12–24 h with apoptotic-mCherry-m-Hepa-1-6 DDCs on cover glass containing a grid (#GC1310, Matsunami Glass Ind., Osaka, Japan), allowing the Muse cells to phagocytose DDCs. The cover glass containing a grid was then fixed with 2.5% glutaraldehyde + 4% PFA in 0.1 M phosphate buffer for 24 h and incubated with DAPI for 5 min. Confocal images were obtained with minimum exposure using a laser confocal microscope to identify phagocytosed mCherry(+)-GFP-h-Muse cells. The region of interest was identified by the confocal images, and images were recorded with care not to bleach the area. The sample was washed in 0.1 M phosphate buffer (5 × 2 min) and post-fixed in 1% osmium tetroxide in 0.1 M phosphate buffer for 2 h on ice. Cells were washed three times in 0.1 M phosphate buffer and rinsed in distilled water. The samples were dehydrated through increasing concentrations of ethanol and finally in propylene oxide and impregnated with epoxy resin; the cover glass was removed by hydrofluoric acid for 1 h on ice, cut into ultrathin sections, and observed under an electron microscope (JEM-1011, JEOL, Akishima, Tokyo, Japan).

### Immunoelectron microscopy

All immunoelectron microscopy procedures were performed as described previously [97]. GFP-h-Muse cells that had been incubated for 3 days with apoptotic-mCherry-m-Hepa-1 DDCs were harvested, and phagocytosed GFP-h-Muse cells with mCherry + were sorted by a cell sorter. The collected cells were plated onto dishes for 24 h, fixed with 4% PFA + 0.5% glutaraldehyde in phosphate buffer, incubated with 3% bovine serum albumin (MilliporeSigma) and 5% goat serum in PBS for blocking. The samples were then reacted with rabbit anti-mCherry antibody (1:10,000, Abcam, ab167453) followed by FluoroNanogold-anti-rabbit IgG (1:200, Nanoprobes, Yaphank, NY, USA, 7204) and then subjected to silver intensification and gold toning as described previously [97]. The anti-mCherry antibody was omitted for the negative control. The samples were dehydrated through increasing concentrations of ethanol and finally in propylene oxide, impregnated with epoxy resin, cut into ultrathin sections, and observed under an electron microscope (JEM-1011, JEOL, Akishima, Tokyo, Japan).

### Annexin V treatment

For incubation of h-Muse cells with apoptotic DDCs (m-cardiomyocytes), 2 × 10^5^ DDC cells were treated to induce apoptosis as described above, followed by treatment with 1 μg/ml annexin V (BioVision, Milpitas, CA, USA) in DMEM with 10% FBS for 8 h [98]. Annexin V-pretreated DDCs were supplied to 1 × 10^5^ h-Muse cells and cultured in DMEM with 10% FBS and 1 μg/ml annexin V for 3 and 5 days, and 1, 2, and 3 weeks for qPCR analysis.

For neutralization of annexin V, 5 μg/ml of anti-annexin V antibody (clone RUU-WAC2A; Nordic-MUbio, Susteren, Netherlands, GTX103250) was added to the annexin V incubation medium for apoptotic-m-cardiomyocytes [98]. For evaluation of the cytotoxicity of annexin V, 1 × 10^4^ h-Muse cells were cultured in the presence of 1 μg/ml and 5 μg/ml annexin V, and the amount of lactase dehydrogenase (LDH) released into the cultured medium was then calculated by LDH release assay (Cytotoxicity LDH Assay Kit; Dojin Chemical, Kumamoto, Japan) according to the manufacturer’s protocol.

For incubation of 1 × 10^4^ h-MSCs with apoptotic DDCs (m-adipocytes), 1 × 10^5^ DDC cells were treated to induce apoptosis as described above, followed by treatment with 1 μg/ml annexin V (BioVision) in DMEM with 10% FBS for 8 h. Annexin V-pretreated DDCs were supplied to 1 × 10^5^ h-MSCs and cultured in DMEM with 10% FBS and 1 μg/ml annexin V for 3 days, and 1 and 2 weeks for qPCR analysis.

For incubation of 1 × 10^4^ r-NSCs with apoptotic DDCs (h-neural cells), 1 × 10^5^ DDC cells were treated to induce apoptosis as described above, followed by treatment with 1 μg/ml annexin V (BioVision) in DMEM with 10% FBS for 8 h. Annexin V-pretreated DDCs were supplied to 1 × 10^5^ r-NSCs and cultured in Neurobasal medium with B27 supplement and 1 μg/ml annexin V for 3 days, and 1 and 2 weeks for qPCR analysis.

### Small interference-RNA for phagocytic receptors

For siRNA transfection, 2 × 10^5^ h-Muse cells were cultured in 6-well plates. Cells were transfected with siRNA as follows: 20 nM of the siRNA was added to 200 μl of Opti-MEM (Invitrogen) and mixed gently. At the same time, 2 μl of Lipofectamine RNAiMax (Invitrogen) was added to 200 μl of Opti-MEM. After 5 min, the Opti-MEM and siRNA solution was mixed gently with the Opti-MEM and Lipofectamine RNAiMax solution. After 20 min, the mixture was added to the wells, and the plates were incubated at 37 °C and 5%CO_2_. After 48 h, total RNA was collected using the NucleoSpin^®^ RNA XS. For Western blotting, protein was collected at 2, 3, 4, and 5 days after transfection with the siRNA. The protein amount in each sample was measured using a BCA protein assay kit, and samples corresponding to 5 μg protein were loaded for Western blotting. siRNA was performed using the following: *RAGE*: siGENOME Human *AGER* (177) siRNA (Horizon, Cambridge, UK); *CD36*: siGENOME Human *CD36* (948) siRNA (Horizon); *CD91/LRP-1*: Silencer Select, s8279 (ThermoFisher Scientific); and *ITGB3*: Silencer Select, s534922 (Thermo Fisher Scientific).

For preparation of phagocytic receptors suppressed 4-siRNA-h-Muse cells, 2 × 10^5^ GFP-h-Muse cells were cultured in 6-well plates. At day1 and day3, GFP-h-Muse cells were transfected with four kinds of siRNA (*RAGE, CD36, CD91/LRP-1* and *ITGB3*) simultaneously. For in vitro experiment, GFP-h-Muse cells 5 days after transfection were incubated with DDCs derived from 4 × 10^5^ apoptotic m-cardiomyocytes for 3 days and 7 days and performed qPCR to detect human specific cardiomyocyte markers. For in vivo experiment, 5 × 10^4^ naïve GFP-h-Muse cells and siRNA-GFP-h-Muse cells were injected into the post-infarct region of BCCAO model 2 days after infarction.

### Mouse genome and mRNA detection in h-Muse cells

GFP-h-Muse cells that phagocytosed apoptotic mCherry-m-Hepa1 DDCs were sorted by the cell sorter after 24 h incubation. Medium containing annexin V was changed every 8 h. Genomic DNA was collected using the REDExtract-*N*-Amp Tissue PCR kit protocol (MilliporeSigma). Mouse genomic DNA in h-Muse cells was detected using a mouse gDNA detection kit (Primerdesign, UK) according to the manufacturer’s instructions. A standard curve was used to measure the amount of mouse genomic DNA in h-Muse cells.

For fractionation of the cytoplasmic fraction, the samples were processed using an SF PTS Kit (GL Sciences, Tokyo, Japan) according to the manufacturer’s instructions. Then, total RNA in the cytoplasmic fraction was purified using the NucleoSpin^®^ RNA XS. qPCR for detection of mouse mRNA in h-Muse cells was performed using a TaqMan gene expression assay with the following mouse-specific primers: *Foxa2*, Mm01976556_s1; *Hnf4a*, Mm01247712_m1; and *Afp*, Mm00431715_m1.

### Chromatin immunoprecipitation sequencing

HL-1 cells infected with pWPXL-*GATA-4* promoter-HA tag-GATA-4-T2A-mCherry lentivirus were generated for ChIP sequencing. mCherry-positive cells were isolated by FACS, treated with etoposide and were used for DDCs. The h-Muse cells were incubated with DDC-fragments for 32 h and then fixed with 1% formaldehyde for 10 min at room temperature to crosslink histone and non-histone proteins to DNA. The formaldehyde was inactivated by the addition of 1.5 M glycine for 5 min. After washing with PBS, the cells were suspended in lysis buffer (50 mM Tris–HCl pH8.0, 10 mM EDTA pH8.0, 1% sodium dodecyl sulfate (SDS), 0.5 mM phenylmethylsulfonyl fluoride (PMSF), and cOmplete protease inhibitor cocktail (MilliporeSigma) and were incubated for 20 min at 4 °C. Cells were sonicated for 10 cycles of 60 s on/ 60 s off on high power using a Bioruptor II (Sonicbio, Kanaagawa, Japan). Samples were centrifuged at 10,000*g* for 10 min at 4 °C, and supernatants were collected and were tenfold diluted using ChIP dilution buffer (50 mM Tris–HCl pH8.0, 150 mM NaCl, 1% TritonX-100, 0.1% sodium deoxycholatye, 0.5 mM PMSF, and cOmplete protease inhibitor cocktail). Chromatin extracts containing DNA fragments with an average size of 500 bp were incubated overnight using 5 μg anti-HA tag antibody (ab9110; ChIP grade, Abcam) at 4 °C under rotation. Chromatin extracts containing DNA fragments before immunoprecipitation were used as an input sample. Antibody-protein complexes were immunoprecipitated using Dynabeads Protein G (ThermoFisher Scientific) at 4 °C for 3 h under rotation. Beads were washed five times with cold RIPA buffer (50 mM Tris–HCl pH7.5, 150 mM NaCl, 1 mM EDTA, 1% TritonX-100, 0.1% SDS), followed by one wash in cold Tris–EDTA buffer. Immunoprecipitated chromatin was eluted at 65 °C with agitation for more than 4 h in elution buffer (10 mM Tris–HCl pH8.0, 300 mM NaCl, 5 mM EDTA, 0.5% SDS). Proteinase K was added overnight at 65 °C to reverse the crosslinking. Samples were treated with RNase A, and the DNA was purified by ethanol precipitation. DNA libraries were prepared by NEBNext Ultra II DNA Library Prep Kit for Illumina (New England BioLabs). All libraries were sequenced using the NextSeq 500 (Illumina, CA, USA) to a read depth of at least 3,000,000 reads/sample. The ChIP sequencing and initial analysis were performed by Kazusa Genome Technologies (Chiba, Japan). This study was analyzed with one biological replicate per group. For data analysis, ChIP sequencing reads were aligned to reference genome (UCSC hg19) using Bowtie (v1.1.2) under default parameters in the Maser Platform (https://cell-innovation.nig.ac.jp/) [99]. Peaks were called using ZINBA under default parameters with a *q* value of 0.001 [100]. Differential peaks were called using HOMER (v4.7) [101] and STAMP [102]. Human iPS cell-derived cardiomyocyte ChIP data were downloaded from the NCBI Gene Expression Omnibus (GSE159411, GSE85631) [51, 52].

### Co-immunoprecipitation for RNA polymerase II and transcription factor IIB

GFP-h-Muse cells that phagocytosed DDC-fragments (apoptotic GATA-4-mCherry reporter m-HL-1) were sorted by cell sorter after incubating for 8 h. The cells were added to lysis buffer (10 mM HEPES (pH8.0), 10 mM KCl, 1.5 mM MgCl_2_, 1 mM DTT, protease inhibitor and 1.25% NP-40) to a final volume of 1 × 10^7^ cells/200 μl. The cells were incubated at 4 °C for 5 min before centrifugation at 8000 rpm for 5 min at 4 °C to fractionate the nucleic and cytosolic fraction. The pellet of nucleic fraction was added to lysis buffer (20 mM HEPES (pH7.5), 20% glycerol, 100 mM NaCl, 0.25 mM EDTA, 1.5 mM MgCl_2_, 7.5 mM NaF, 0.1 mg/ml BSA, 1 mM DTT, 0.025% NP-40 and protease inhibitor) and incubated at 4 °C for 30 min before centrifugation at 13000 rpm for 5 min at 4 °C. The supernatant was collected and incubated for overnight at 4 °C with 1 μg of anti-mCherry antibody (immunoprecipitation grade, BioLegend) and Dynabeads protein G (20 μl, Thermo Fisher Scientific). Beads were washed six times for 5 min each time with 1 ml of wash buffer (10 mM Tris–HCl (pH8.0), 100 mM NaCl, 2 mM EDTA (pH8.0) and 0.01%NP-40) on ice using a Dynamag-2 magnet (Thermo Fisher Scientific). Proteins were eluted from beads in 20 μl 1 × SDS –PAGE sample buffer and subjected to SDS-PAGE electrophoresis and Western blotting with rabbit anti-mCherry (1:1000, Abcam, ab183628), mouse anti-RNA polymerase II (1:1000, Abcam, ab817) and rabbit anti-transcription factor II B (1:1000, Abcam, ab109106) as described above. Rabbit and mouse normal IgG were used for negative control.

### Lysosomal activity

Lysosomal activity was assessed using a Lysosomal Intracellular Activity Assay Kit (BioVision, K448-50) according to the manufacturer’s instructions. As a positive control, C57/B6 mouse peritoneal macrophages were used.

### Statistics

The data were analyzed by one-way analysis of variance and an unpaired Student’s t-test to determine statistical significance using Microsoft^®^ Excel software. All data represent three independent experiments. The results are presented as the mean ± SE of the mean. A *p* value of less than 0.05 was considered significant.

## Supplementary Information

Below is the link to the electronic supplementary material.

**Movie 1.** Intracellular calcium dynamics after biochemical depolarization with 70 mM KCl indicated by GFP intensity in GCaMP-h-Muse cells incubated with m-cardiomyocyte fragments and damaged cardiac tissue (MP4 1996 KB)

**Movie 2.** Time-lapse of GFP-h-Muse cells incubated with mCherry-m-Hepa1 DDCs for 24 h analyzed by Imaris software (MP4 30750 KB)

**Movie 3.** Time-lapse of *GATA-4*p-mCherry-h-Muse cells incubated with GFP-m-cardiomyocyte DDCs (MP4 2426 KB)

**Movie 4.** Time-lapse of* GATA-4*p-mCherry-h-Muse cells incubated with GFP-m-cardiomyocyte DDCs in laser confocal microscopy (MP4 5031 KB)

**Movie 5.** Time-lapse of *SOX9*p-mCherry-h-MSCs incubated with GFP-m-chondrocyte DDCs in laser confocal microscopy (MP4 9446 KB)

**Movie 6.** Time-lapse of GFP-h-Muse cells incubated with annexin V-pretreated mCherry-m-Hepa1 DDCs for 24 h analyzed by Imaris software (MP4 37848 KB)

**Movie 7.** Multiphoton laser scanning microscopy live-cell imaging of *NEUROD1*p-CFP expression in phagocytosing mCherry-h-Muse cells after being injected into the infarcted area of a GFP-mouse focal stroke model (MP4 743 KB)

Supplementary file8 (DOCX 27467 KB)

## Data Availability

The data that support the findings discussed here are available from the corresponding author upon reasonable request.
