# Supplementary material for: Phagocytosing differentiated cell-fragments is a novel mechanism for controlling somatic stem cell differentiation within a short time frame
Source: Cell Mol Life Sci. 2022 Oct 6;79(11):542. doi: 10.1007/s00018-022-04555-0 (PMC9537123; doi:10.1007/s00018-022-04555-0)
Supplement: Supplementary file 8 — Supplementary file8 (DOCX 27467 KB) [file 18_2022_4555_MOESM8_ESM.docx]

**Supplementary Information and Figures**

**SI 1) Evaluation of apoptotic-cell fragments.**

Floating dead cell fragments were collected from apoptotic m-cardiomyocytes by centrifugation after treatment with etoposide. These fragments generated no adherent living cells after they were re-plated to a culture dish for up to 7 days, indicating that the samples contained no living cells. Soon after collection, 99.5±0.2% of the fragments were positive for propidium iodide (PI) staining, an indicator of dead cells. Therefore, contamination by live cells was unlikely when the floating cells were collected 24 h after etoposide treatment.


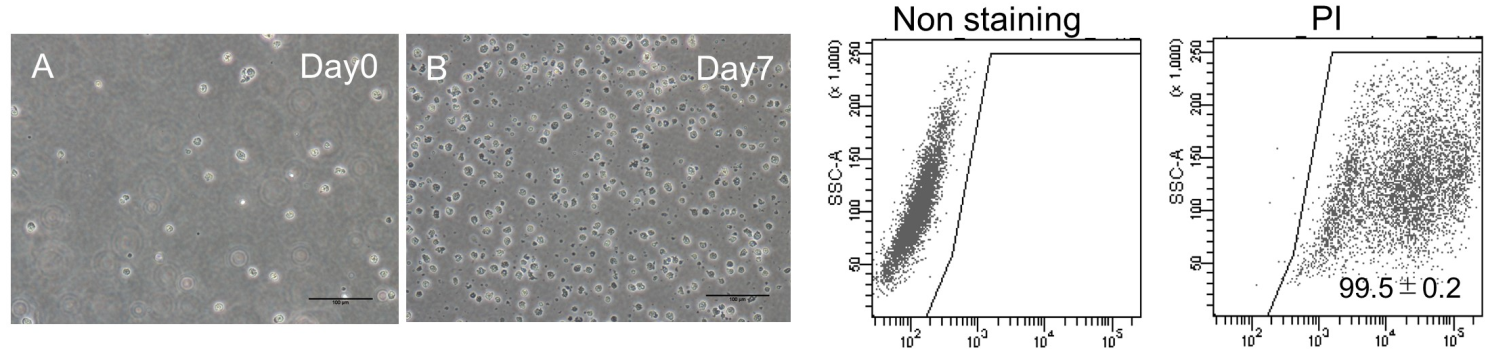


**Figure S1. Apoptotic-cell fragments of m-cardiomyocytes.** (A) m-cardiomyocytes treated with 50 μM etoposide for 24 h. Floating dead cell fragments were collected and plated on a culture dish. The picture was taken soon after plating. (B) No adherent live cells were observed 7 days after plating the cell fragments. Bars = 100 μm. (C) PI staining of apoptotic-cell fragments derived from m-cardiomyocytes.

**SI 2) Controls for immunocytochemistry.**  **
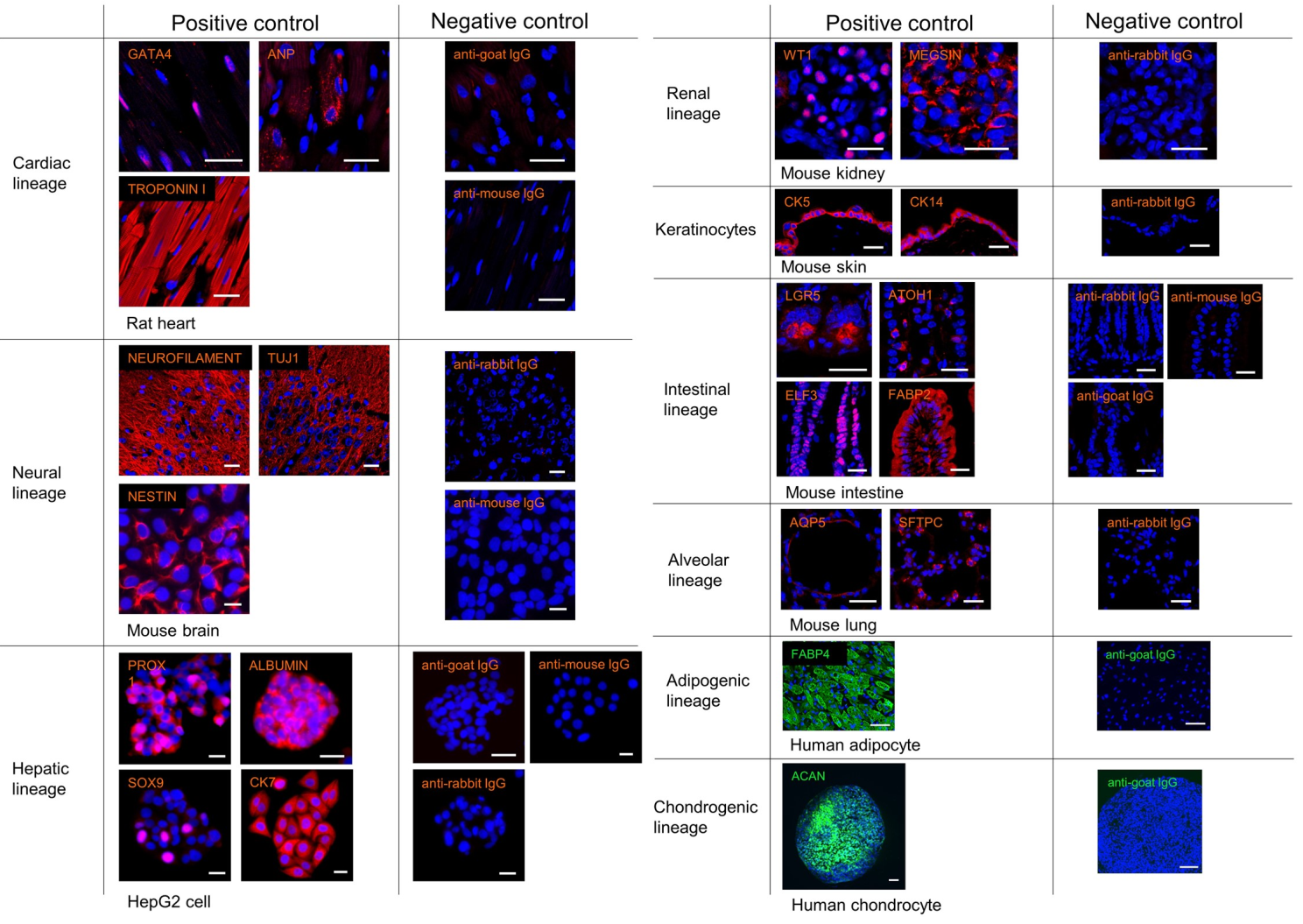
Figure S2A. Positive and negative controls for each immunocytochemical marker.** Bars = 50 μm.


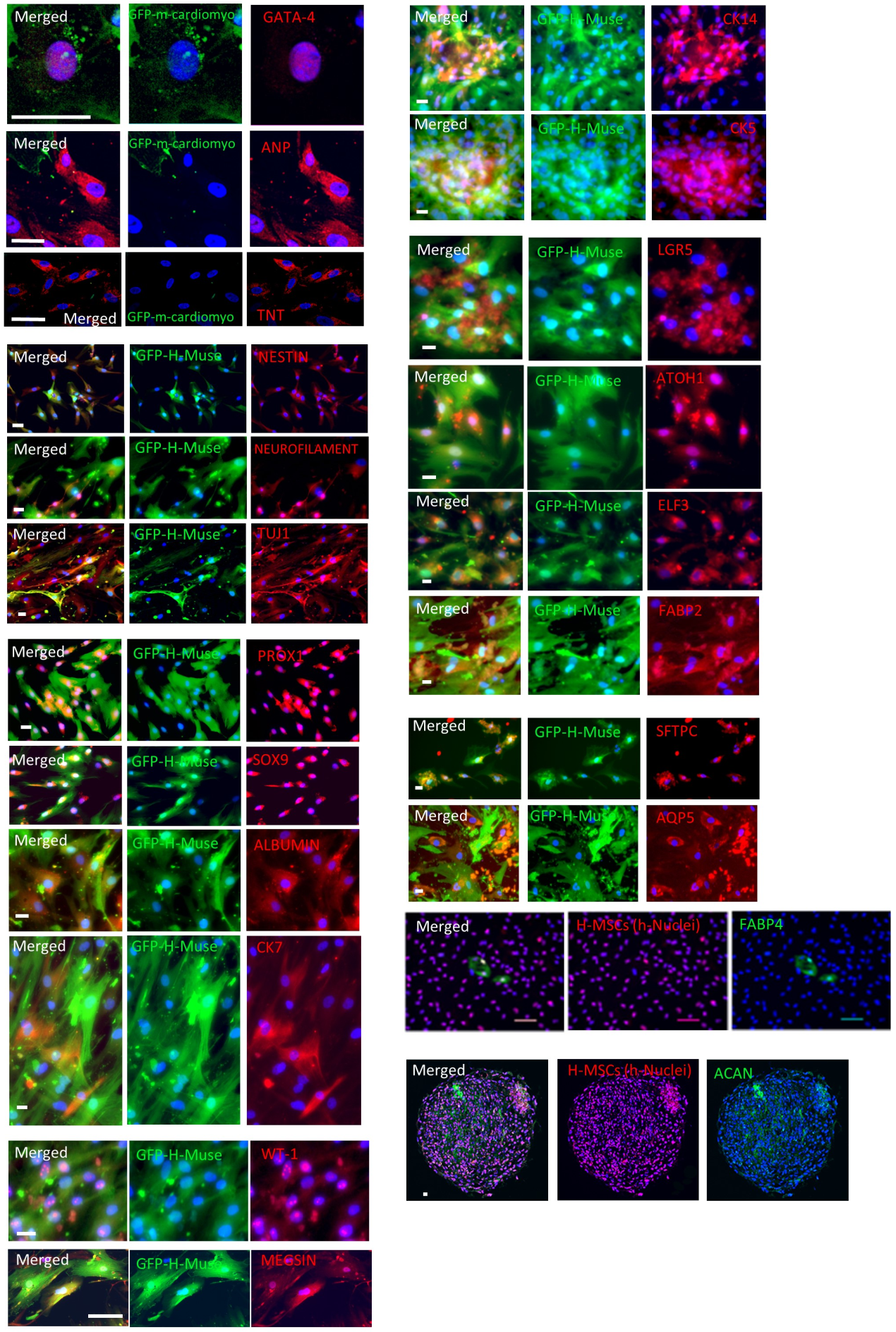


**Figure S2B. Color codes for the immunocytochemical images.** Bars = 25 μm.

NESTIN, NEUROFILAMENT, and TUJ1 images are low magnification images of Figure1G.


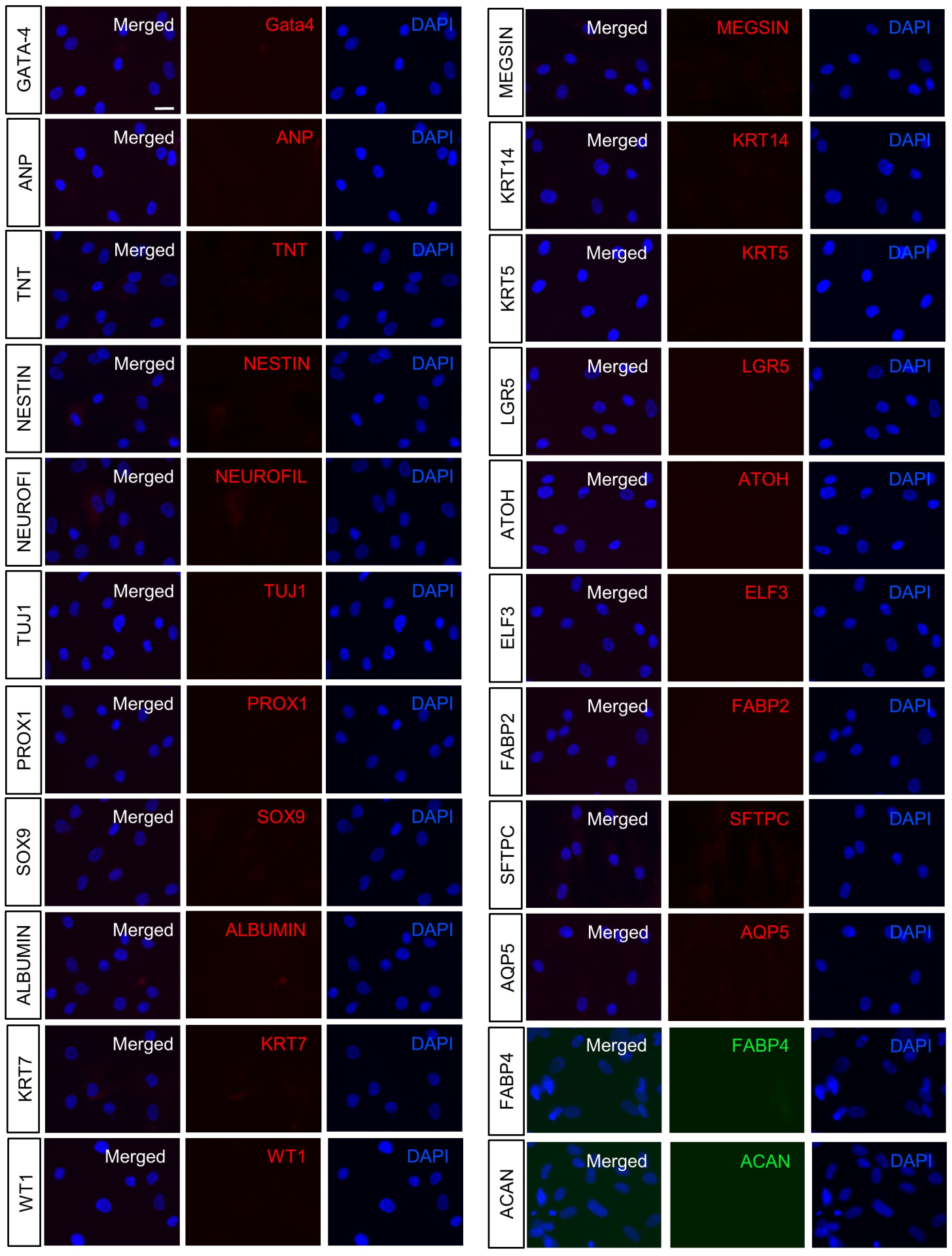


**Figure S2C. Immunocytochemistry of naïve Muse cells.**

None of the differentiation markers examined were expressed in naïve Muse cells. Bar = 50 μm.

**SI 3) Differentiation marker expression in h-Muse cells after incubating with rat apoptotic fragments.**

Rat differentiated cells were selected from mesodermal-, ectodermal-, and endodermal-lineages and apoptotic fragments were supplied to h-Muse cells for the first 3 days and then washed out. The h-Muse cells were transduced with GFP-lentivirus for identification in immunocytochemistry. Confirmed species-specific primers were used for quantitative reverse transcription-PCR (qPCR).

Co-culture with apoptotic renal cell fragments induced the expression of the human-specific progenitor markers wilms tumor protein (*WT-1*) and –EYA transcriptional coactivator and phosphatase 1 (*EYA1*) at D3 (p<0.001) in h-Muse cells. The maturity marker human-aquaporin-1 (*AQP1*) was faintly expressed at D3 and upregulated at D14 and D21 (p<0.001; Fig. S3A). Ectodermal (human-keratin 10 [*KRT10*], -neuronal nuclei [*NEUN*]) and endodermal (human-podoplanin [*PDPN*], -alpha fetoprotein [*AFP*]) markers were consistently not detected by qPCR up to D21. Immunocytochemistry revealed expression of WT-1 at D14 and MEGSIN at D21 in h-Muse cells (Fig. S3B).

Keratinocyte fragments induced the expression of the stem cell markers human-tumor protein p63 (*P63)* and -keratin15 (*KRT15*) at D3 (p<0.001), and the keratinocyte marker human desmoglein-3 (*DSG3*) at D5 (p<0.001). Another keratinocyte marker, human-*KRT10,* was faintly expressed at D3, upregulated at D14, and maximum expression was observed at D21 (p<0.001; Fig. S3C). Mesodermal (human-NK-2 transcription factor related, locus 5 [*NKX2.5*], -atrial natriuretic peptide [*ANP*], -troponin-T [*TNT*]) and endodermal (human-prospero homeobox protein-1 [*PROX1*], *-*leucine rich repeat containing G protein-coupled receptor 5 [*LGR5*], *-PDPN*) markers were consistently under the detection limit in h-Muse cells in qPCR. Two keratinocyte markers, cytokeratin-14 (KRT14) and cytokeratin-5 (KRT5), were expressed at D21 in h-Muse cells (Fig. S3D).

Intestinal cell fragments induced human-*LGR5* (stem cell), -atonal BHLH transcription factor 1 (*ATOH1*; secretory progenitor), and –growth factor independent 1 transcriptional repressor (*GFI1*; goblet and Paneth cell progenitors) at D3 (p<0.001); and –lysozyme 1 (*LYZ1*; Paneth cell) and –fatty acid binding protein 2 (*FABP2*; enterocyte) at D8 (p<0.001) in h-Muse cells (Fig. S3E). Mesodermal (human-*NKX2.5*, -*ANP*, -*TNT*) and ectodermal (human-*KRT10*, -*NEUN*) markers were consistently under the detection limit in qPCR. LGR5 and ATOH1 were detectable in h-Muse cells at D5, E74 like ETS transcription factor 3 (ELF3; enterocyte progenitor) at D14, and FABP2 at D21 by immunocytochemistry (Fig. S3F).

Alveolar cell fragments induced human-*PDPN* (progenitor/type 1 alveolar cell), aquaporin-5 (*AQP5*; type 1 alveolar cell), sodium channel epithelial 1α subunit (*SCNN1A*; lung epithelial), and cadherin 1 (*CDH1*; lung epithelial) at D3 (p<0.001), and these markers were upregulated over time (Fig. S3G). Mesodermal (human-*NKX2.5*, -*ANP*, -*TNT*) and ectodermal (human-*KRT10*, -*NEUN*) markers were consistently under the detection limit in qPCR. Surfactant protein-C (SFTPC; progenitor/ type 2 alveolar cell) was expressed at D7 and AQP5 at D14 in immunocytochemistry in h-Muse cells (Fig. S3H).

The percentage of each lineage-specific marker in h-Muse cells is summarized in Fig. S3I. Positive and negative controls for each marker in immunocytochemistry (Fig. S2A) and the color codes for immunocytochemistry (Fig. S2B) are shown in Fig. S2. Immunocytochemistry confirmed that expression of the differentiation markers was negative in naïve Muse cells (Fig. S2C).


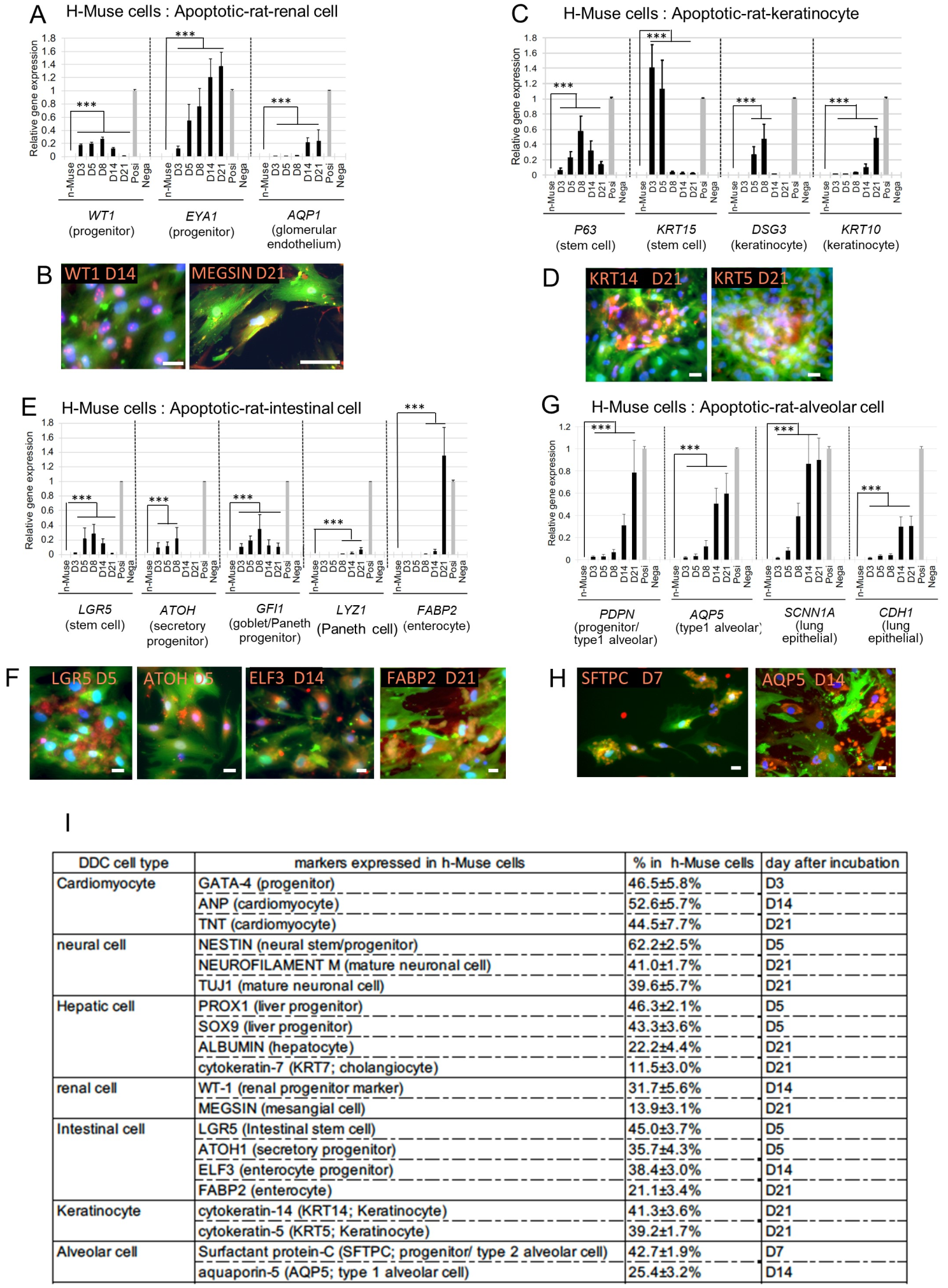


**Figure S3. Differentiation marker expression in h-Muse cells after incubating with rat apoptotic fragments.** (A, C, E, G) Lineage-specific marker expression in qPCR and (B, D, F, H) immunocytochemistry in h-Muse cells after incubation with rat apoptotic cells. In qPCR, naïve h-Muse cells (n-Muse) and apoptotic cell fragments (Nega) were used as negative controls. ***: p<0.001. For the positive control, human fetus total RNA was used for renal cells, and keratinocytes; human small intestine RNA was used for intestinal cells; and human fetal lung RNA was used for alveolar cells. Graphs are represented as mean ± SEM. Bars; B, D, F, H = 25 μm. (I) Percent of marker expression in GFP-h-Muse cells after incubation with rat apoptotic cell fragments.

**SI 4) Cardiac marker expression in h-Muse cells after incubation with apoptotic m-HL-1-fragments.**

To evaluate the differentiation of h-Muse cells in qPCR assays, confirmed species-specific primers were used. When h-Muse cells were incubated with cell fragments derived from apoptotic m-cardiac muscle cell line (m-HL-1)-fragments at a 1:2 ratio for 3 days and then the fragments were washed out, the h-Muse cells became positive for human-*NKX2.5* and – GATA binding protein 4 (*GATA-4*) at D3 after starting the incubation and the positivity was maintained at D21. The h-Muse cells became positive for human-*ANP* at D7 and for human-*TNT* at D14. Ectodermal (human-*KRT10*, -*NEUN*) and endodermal (human-*PROX1*, *-LGR5*, *-PDPN*) markers were consistently under the detection limit.


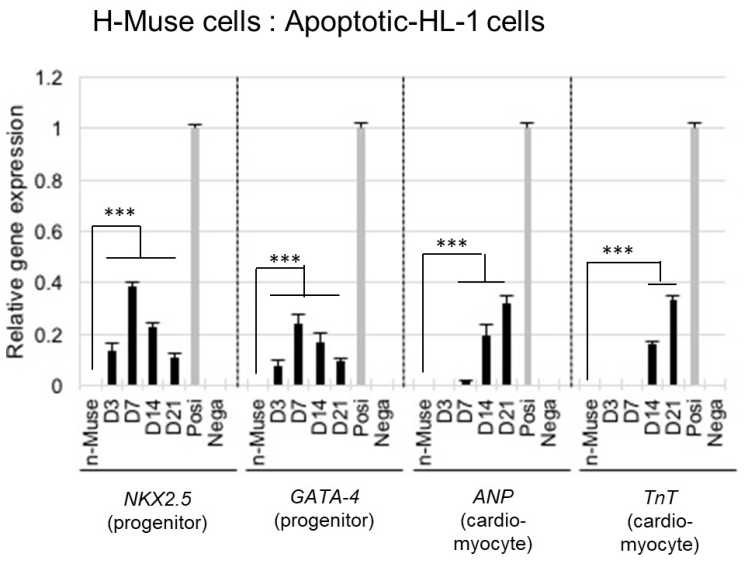


**Figure S4. Cardiac marker expression in h-Muse cells after incubation with apoptotic m-HL-1 fragments.**  qPCR of human-specific *NKX2.5, GATA-4*, *ANP*, and *TNT* in h-Muse cells after incubation with apoptotic m-HL-1 fragments. Naïve h-Muse cells (n-Muse) and apoptotic cell fragments (Nega) were used as negative controls, and human fetal heart total RNA was used as a positive control (Posi). ***: p<0.001

**SI 5) Evaluation of fusion by FISH analysis.**

Cultures of the apoptotic mouse liver cell line Hepa1-6 (m-Hepa1-6) comprised dead cell fragments. Even so, it is considered that these cells might be revived by interacting with h-Muse cells during incubation. If they were revived, expression of the above-mentioned differentiation markers might be explained by the fusion of dead m-Hepa1-6 fragments and h-Muse cells. Therefore, to test the propensity of Muse cells to fuse with dead cells, intact m-Hepa1-6 and h-Muse cells were co-cultured at a 1:1 ratio and fluorescence in situ hybridization (FISH) was performed. While over 4500 cells were analyzed, no fused cells were detected. Similarly, fusion between apoptotic m-Hepa1-6 fragments and h-Muse cells incubated at a 1:2 ratio for 3 days was not detected. Thus, fusion does not appear to be a major mechanism of h-Muse cell differentiation.


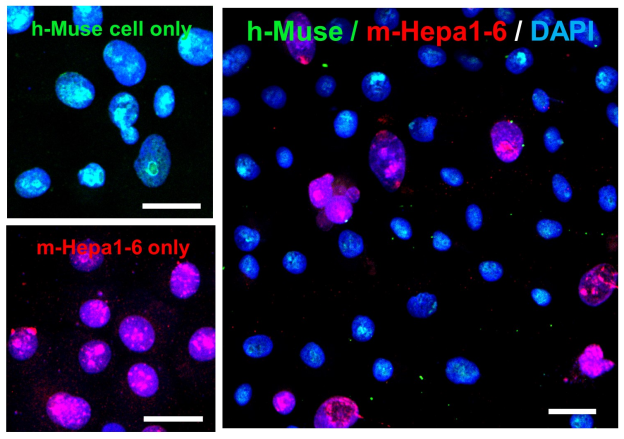


**Figure S5. FISH analysis.** Co-culture of h-Muse cells and intact m-Hepa1-6 at a 1:1 ratio for 3 days. Human nuclei are labeled green and mouse nuclei are labeled red. At 3 days, fusion between h-Muse cells and m-Hepa1-6 could not be detected, even after counting ~4500 cells. Incubation of h-Muse cells with apoptotic-m-Hepa1-6-fragments at a 1:2 ratio for 3 days showed the same tendency. Bars = 50 μm.

**SI 6) Effect of conditioned medium, cell extract, and extracellular vesicles from apoptotic cells.**

Apoptotic cells were prepared from m-cardiomyocytes and m-HL-1 cells.

**Conditioned medium:**  One possible explanation for our observations is that humoral factors initiated the commitment of Muse cells to a specific-cell lineage. To test this possibility, we collected conditioned media from intact and apoptotic m-cardiomyocyte cultures at numbers that were 2x, 10x, or 100x higher than that of the h-Muse cells. These 6 kinds of conditioned media were centrifuged at low speed and passed through a 0.45-µm filter to remove intact cells and cell fragments. The h-Muse cells were then incubated with the conditioned medium, and qPCR was performed at 3, 7, 14, and 21 days. The conditioned media contained factors relevant to cardiac differentiation [1]; e.g., the conditioned medium from the culture containing 100x more apoptotic m-cardiomyocytes than Muse cells contained hepatocyte growth factor (HGF), cardiotrophin-1, and transforming growth factor beta 1 (TGF-β1; Fig. S6A). Human-specific cardiac markers, however, were consistently under the detection limit in h-Muse cells treated with any of the 6 kinds of conditioned media at any time-point. The same experiment was repeated using m-Hepa1-6 and mouse skeletal muscle cell line C2C12 (m-C2C12). Human-specific *PROX1*, α-fetoprotein (*AFP*), or albumin for incubation with m-Hepa1-6 conditioned media, and human-specific paired box 6 (*PAX7*), myoblast determination protein 1 (*MYOD*), and *MYOGENIN* for m-C2C12 were under the detection limit for all conditions and time-points examined.

**Cell extract:**  To test the possible involvement of cell extracts in Muse cell differentiation, intact m-cardiomyocyte cell extract was prepared by sonication. To avoid contamination by living cells in the apoptotic cell extracts, only floating dead cell fragments were collected after etoposide treatment, centrifuged, and subjected to sonication. Six cell extracts of intact and apoptotic m-cardiomyocyte fragments from cultures containing a 2x, 10x, and 100x or greater number of m-cardiomyocytes than h-Muse cells were prepared and incubated with h-Muse cells. The cell extract containing 100x or more apoptotic m-cardiomyocyte fragments than Muse cells contained HGF, cardiotrophin-1, and TGF-1 (Fig. S6A). Human-specific cardiac markers, however, were under the detection limit in all conditions examined and at all time-points from 3 to 21 days. The same result was obtained with m-Hepa1-6 and m-C2C12 cells.

**Extracellular vesicles:**  Another possible explanation for the h-Muse cell differentiation is that secreted extracellular vesicles containing cargo initiated the differentiation. To test this possibility, extracellular vesicles positive for cluster of differentiation 63 (CD63), tumor susceptibility 101 (TSG101), and 70 kDa heat shock proteins (HSP70) were collected by ultracentrifugation of medium from intact and apoptotic m-HL-1 cells (Fig. S6B, S6C). Extracellular vesicles contain microRNAs that regulate lineage specification[2]. Intact and apoptotic m-HL-1-derived extracellular vesicles contained miR-1, miR-133a, miR206, miR208, and miR-499, which are related to cardiac differentiation (Fig. S6D). Although the amount of extracellular vesicles required for differentiation induction differed among cell types in previous reports, most cases fall into the range of 25~100 μg/ml [3, 4]. Therefore, we supplied 50 μg/ml and 200 μg/ml extracellular vesicles derived from intact and apoptotic m-HL-1 cells to h-Muse cells. Human-specific markers *NKX2.5*, *GATA-4*, *ANP*, and *TNT*, however, were under the detection limit in all conditions examined and at all time-points from 3 to 21 days. The uptake of extracellular vesicles into h-Muse cells was confirmed by incubating the h-Muse cells with PKH67-labeled extracellular vesicles for 3 days (Fig. S6E).


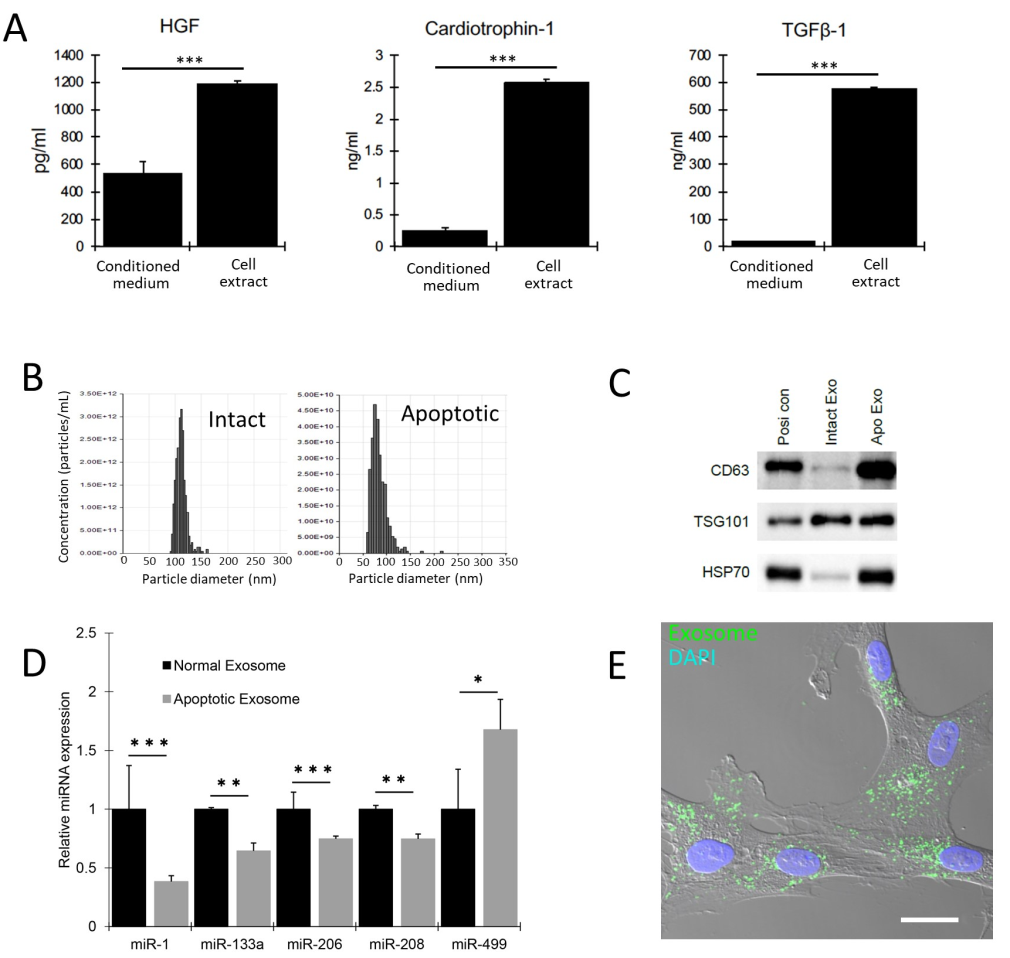


**Figure S6. Conditioned medium, cell extract, and extracellular vesicles from apoptotic-m-cardiomyocytes had limited effects on cardiac marker expression in h-Muse cells.**

(A) ELISA for HGF, cardiotrophin-1, and TGF-β1 in apoptotic-m-cardiomyocyte-derived conditioned medium and cell extract. According to a previous report, 20 ng/ml HGF, 200 ng/ml cardiotrophin-1, and 2.5 ng/ml TGF-β were used for in vitro cardiac differentiation of Muse cells (Amin, et al., 2018). The amount of TGF-β1 was higher while the amounts of HGF and cardiotrophin-1 were lower in the conditioned medium and cell extract compared with the medium used for in vitro differentiation. ***; p<0.001. (B-E) Collection of extracellular vesicles from m-cardiomyocytes. (B) Tunable resistive pulse sensing analysis of extracellular vesicles derived from intact- and apoptotic-m-cardiomyocytes. (C) Western blots of extracellular vesicles derived from intact- (Intact Exo) and apoptotic- (Apo Exo) m-cardiomyocytes. Positive control was from mouse kidney (Posi con). Samples corresponding to 5 μg protein were loaded in each lane. (D) Cardiac differentiation-related microRNAs in extracellular vesicles derived from intact- (Normal Exosome) and apoptotic- (Apoptotic Exosome) m-cardiomyocytes in qPCR (mean ± SE). *; p<0.05, **; p<0.01, ***; p<0.001. (E) Uptake of apoptotic-m-cardiomyocyte-derived extracellular vesicles into h-Muse cells. Extracellular vesicles (100 μg) prelabeled with green fluorescent PKH67 were added to 2x10^4^ h-Muse cells and incubated for 3 days. Bar = 25 μm.

**SI 7) Macrophage marker expression in h-Muse cells and phagocytic activity of h-MSCs.**


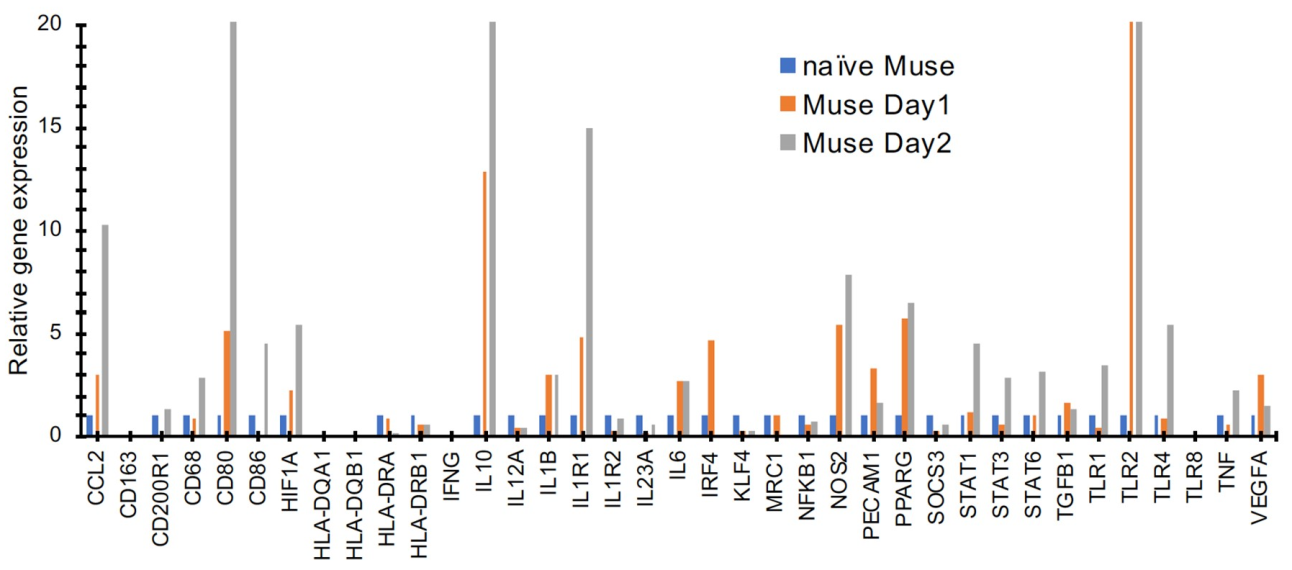


**Figure S7.** qPCR of macrophage markers in naïve h-Muse cells and h-Muse cells after 1 and 2 days exposure to m-cardiomyocyte-DDCs.

**SI 8) Expression of functional markers in phagocytosed h-MSCs and r-NSCs co-cultured with damaged tissue.**

We investigated whether or not h-MSCs and r-NSCs incubated with apoptotic fragments for the first 3 days and then co-cultured with damaged tissue slices in the Boyden chamber [called D7 (P+T)] had increased gene expression levels of functional markers.

When h-MSCs were incubated with apoptotic-m-chondrocyte DDCs for 3 days and then co-cultured in suspension with a mouse-articular cartilage slice for 7 days, expression of the mature chondrocyte markers h-matrix metalloproteinase 13 (*MMP-13*), h-collagen type X alpha 1 chain (*COL10A1*)*,* h-aggrecan (*ACAN*)*,* h-thrombospondin 4 (*THBS4*)*,* and h-SIX homeobox 1 (*SIX1*) was significantly upregulated compared with those at D21 (*COL10A1*, *ACAN*, and *SIX1*; p<0.05, *MMP13* and *THBS4*; p<0.01). Collagen type II alpha 1 chain (*COL2A1*), undetectable in both naïve h-MSCs and h-MSCs at D21, became positive in qPCR (Figure S8A).

When r-NSCs were incubated with apoptotic-h-neural cell DDCs for 3 days and then co-cultured with a rat-brain tissue slice for 7 days, expression of the functional neural markers hyperpolarization activated cyclic nucleotide gated potassium and sodium channel 2 (*Hcn2*), potassium voltage-gated channel subfamily B member 1 (*Kcnb1*), and sodium voltage-gated channel alpha subunit 2 (*Scn2a*) was significantly upregulated compared with that at D14 (p<0.001). Postsynaptic density protein 95 (*Psd95*), synaptophysin, and myelin basic protein (*Mbp*), undetectable in both naïve r-NSCs and r-NSCs at D14, became positive in qPCR (Figure S8B).


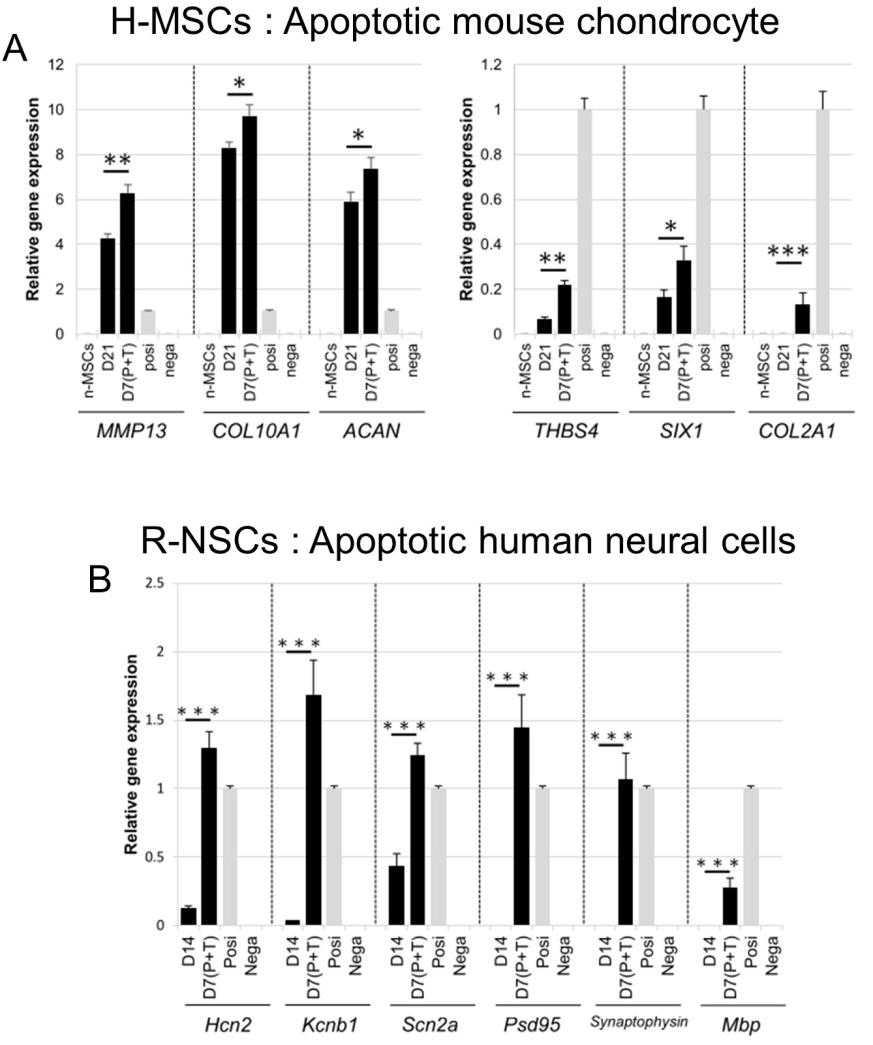


**Figure S8. Functional marker expression of phagocytosed h-MSCs and r-NSCs.** (A) D7 (P+T) refers to h-MSCs incubated with mouse apoptotic chondrocyte fragments for 3 days and then co-cultured in suspension with a mouse articular cartilage slice for 7 days (mean ± SEM). Confirmed species-specific primers were used in the qPCR. Apoptotic cell fragments were used as a negative control (Nega). For the positive control, total RNA obtained from chondrocytes induced from h-MSCs according to the method reported by Crisan M et al. [5] was used (Posi). (B) D7 (P+T) refers to r-NSCs incubated with human apoptotic neural cell fragments for 3 days and then co-cultured with a damaged rat brain tissue slice for 7 days (mean ± SEM). Confirmed species-specific primers were used in the qPCR. Positivity of each marker in adult rat brain total RNA and negativity in h-neural cells were confirmed for each primer.

**SI 9) Single-cell RNA sequencing of h-Muse cells after incubation with apoptotic cell fragments.**

When cell cycle analysis was merged with t-SNE, the majority of naïve-Muse cell-cluster 2, as well as Phago-cardio-Muse, Phago-neuro-Muse 1~3, and Phago-hepa-Muse 1~4 were at G1 phase, whereas naïve 1 was in G2M and S phases (Fig. S9A). Thus, naïve 1 represented an actively proliferating population, whereas h-Muse cells became less proliferative after phagocytosing m-cardiomyocyte-, r-neural cell-, and r-hepatic cell-DDCs, showing that they began to differentiate into a specific lineage compatible with the cell type they had phagocytosed.


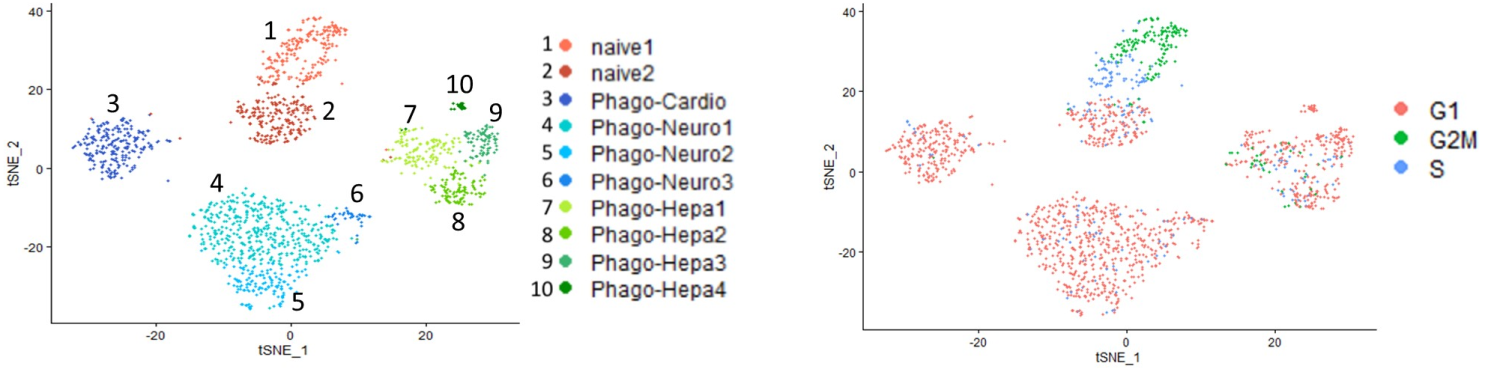


**Figure S9A. Overlay of cell cycle analysis with t-SNE.** T-SNE exhibited 2 clusters in naïve-Muse cells (naïve), 3 clusters in Phago-neuro-Muse, and 4 clusters in Phago-hepa-Muse, for a total 10 clusters including Phago-cardio-Muse, comprised of a single cluster (left, same as Figure 4B). These clusters are overlaid with the cell cycle analysis (right).

Because naïve-Muse cells were separated into subpopulations cluster-1 and cluster-2 by cell cycle as mentioned above, Phago-neuro-Muse and Phago-hepa-Muse groups that were separated into several clusters were further subjected to subpopulation analysis.

Phago-neuro-Muse was separated into the 3 clusters, namely Phago-neuro-Muse 1, -2, and -3. Glial-related markers, such as nuclear factor I X (*NFIX*) [6], semaphorin 3F (*SEMA3F*) [7], slit homolog 3 protein (*SLIT3*) [8], galactocerebrosidase (*GALC*) [9], junction adhesion molecule 3 (*LAM3*) [10], and NGFI-A-binding protein 2 (*NAB2*) [11], were mainly expressed in Phago-neuro-Muse 1/2 clusters; neuronal markers, such as B-cell translocation gene 2 (BTG2) [12], Ras GTPase SynGAP1 [13], bHLH family member e41 (*BHLHE41*) [14], and high-mobility group protein 2 (*HMG-B2*) [15], were mainly expressed in Phago-neuro-Muse 3 (Fig. S9B). Because r-neural cell-DDCs were generated from primary culture of rat fetal hippocampus, both apoptotic-glial and -neuronal cells were exposed to DDCs. Therefore, h-Muse cells that phagocytosed r-neural cell-DDCs, namely Phago-neuro-Muse, were considered to express both glial and neuronal markers.

Phago-hepa-Muse cells were separated into 4 clusters. Marker expression was analyzed with reference to the Human Protein Atlas [16]. Phago-hepa-Muse cluster-1 was characterized by a higher expression of endothelin 1 (*EDN1*), semaphorin 6B (*SEMA6B*), Kruppel-like factor 10 (*KLF10*), and integrin subunit alpha 1 (*ITGA1*), which are related to endothelial cells; Phago-hepa-Muse cluster-2 cells were characterized by the expression of Gardner-Rasheed feline sarcoma viral (*V-FGR*) oncogene homolog (*FGR*), guanylyl cyclase-coupled A (*GCA*), MX2, and C-X-C motif chemokine ligand 10 (*CXCL10*), which are related to Kupffer cells. On the other hand, Phago-hepa-Muse cluster-3/4 cells were higher in methyltransferase-like 7A (*METTL7A*), metallothionein 1M (*MT1M*), aldo-keto reductase family 1 member C2 (*AKR1C2*), nicotinamide N-methyltransferase (*NNMT*), Tsukushi, small leucine rich proteoglycan (*TSKU*), and aldehyde oxidase 1 (*AOX1*), which are related to hepatocytes. Similar to Phago-neuro-Muse cells, Phago-hepa-Muse cells were induced by phagocytosis of r-hepatic cell-DDCs prepared by the apoptotic treatment of primary cultured rat fetal liver. Because the primary culture of the liver contains liver components, hepatocytes, sinusoid endothelial cells, and Kupffer cells, h-Muse cells that phagocytosed r-hepatic cell-DDCs might have differentiated into these lineages.


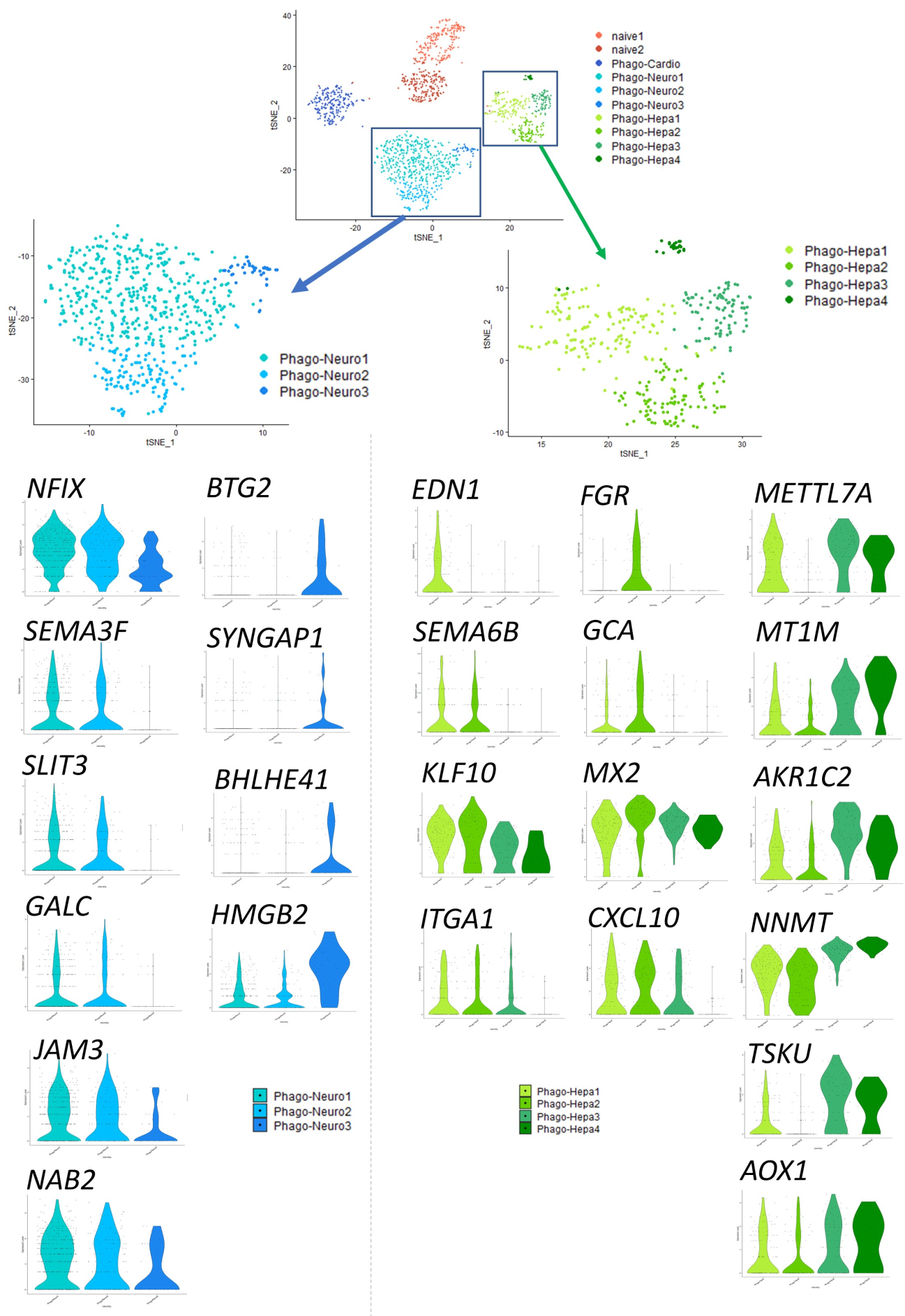


**Figure S9B. Subpopulation analysis of Phago-neuro-Muse and Phago-hepa-Muse cells.** Violin plots of each marker in each cluster are shown.


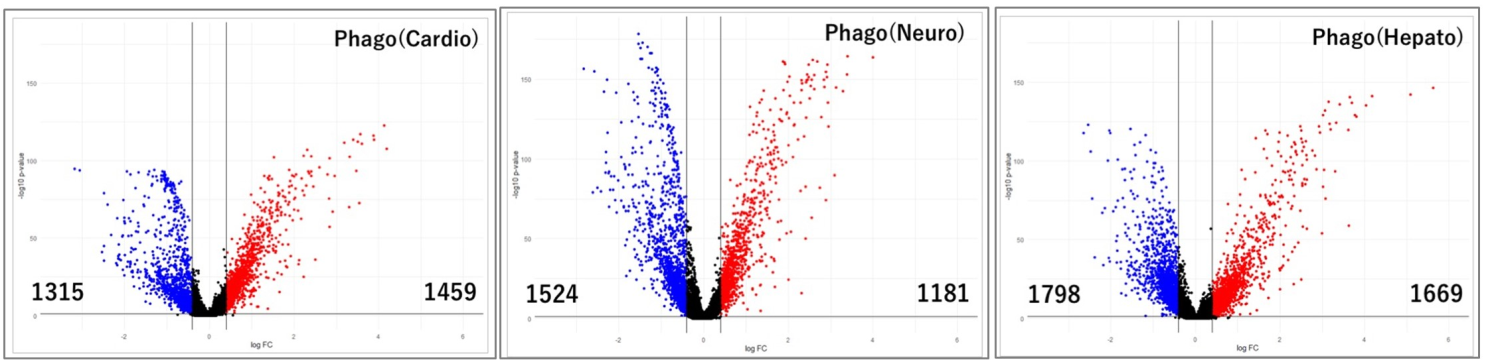


**Figure. S9C. Volcano plot.** Volcano plot of RNA-seq transcriptome data displaying the difference in the gene expression pattern between the naïve-h-Muse cells and each Phago-Muse group. Significantly differentially expressed genes were defined as those with a fold-change (FC) more than 1.5 times or less than 2/3 times, with p<0.05. Upregulated genes are shown in red, downregulated genes are shown in blue, and the black lines represent the boundary for identification of the upregulated or downregulated genes based on the p-value and FC. Genes that were increased (number of genes indicated on the right) and decreased (number of genes indicated on the left) were used for the GO analysis.


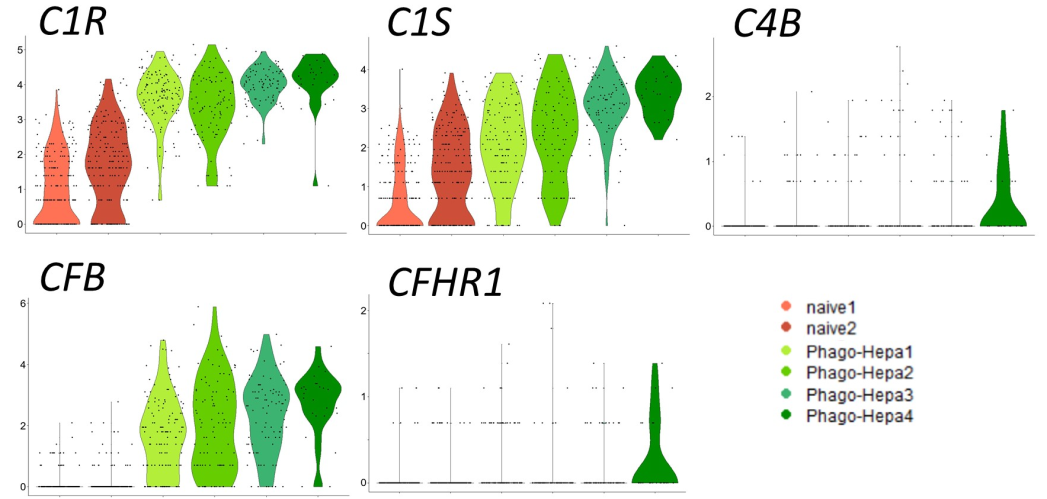


**Figure. S9D. Gene expression analysis of innate immune response in Phago-hepa-Muse clusters.**

Violin plots of innate immune response in Phago-hepa-Muse clusters are shown. complement C1r : *C1R*, complement C1s : *C1S*, complement C4B : *C4B*, complement factor B : *CFB*, and complement factor H related 1: *CFHR1*.

**SI 10) Single-cell RNA sequencing of Phago-cardio-, -neural-, and -hepa-Muse cells in comparison with human fetal cells.**

To compare the similarities and differences between committed h-Muse cells after phagocytosis and authentic human fetal cells, human fetal transcriptome data obtained by single cell RNA-seq was downloaded from the NCBI Gene Expression Omnibus (GSE157329). “Hepatocyte” and “hepatic stellate cell” were extracted as “Fetal (Liver cell)” from the human fetal transcriptome data; “ventricle cardiomyocyte” as “Fetal (Cardiomyocyte)”; “GABAergic neuron”, “MNs”, “MNv”, “Schwann progenitor”, and “sympathetic neuron” as “Fetal (Neuronal cells)”. A total of 200 cells from each fetal cell cluster were randomly selected for comparing gene expression with 1228 single Phago-Muse cells (250 Phago-cardio-Muse cells, 621 Phago-neuro-Muse cells, and 357 Phago-hepa-Muse cells).

In the hierarchy heatmap and Gene Ontology (GO) terms, genes related to cardiac differentiation were identified in both Phago-cardio-Muse cells and Fetal (Cardiomyocyte; red line in Figure S10A). In Figure S10B, genes related to neuronal differentiation ware identified in both Phago-neuro-Muse cells 1 and 2, and Fetal (Neuronal cells; red line in Figure S10B). In Figure S10C, genes related to hepatic differentiation ware identified in both Phago-hepa-Muse cells 1, 2, 3, 4 and Fetal (Liver cell; red line in Figure S10C).


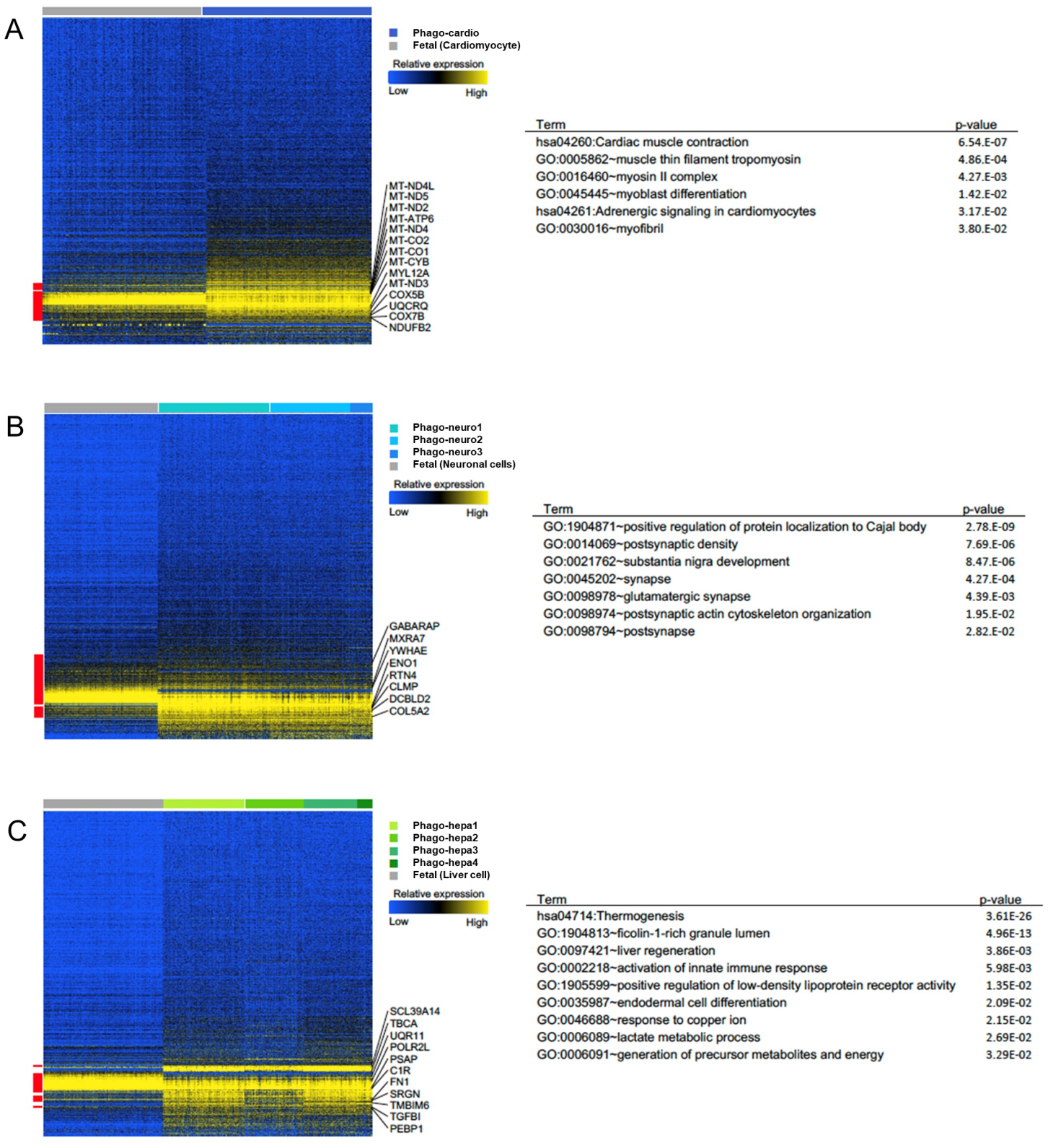


**Figure S10. Hierarchy heat map and GO term analysis.** (A) Hierarchy heatmap and GO term analysis of Phago-cardio-Muse (Phago-cardio) and fetal human cardiomyocytes (Fetal [Cardiomyocyte]). (B) Hierarchy heatmap and GO term analysis of Phago-neuronal-Muse cell clusters (Phago-neuro-1, -2, -3) and fetal human neuronal cells (Fetal [Neuronal cell]). (C) Hierarchy heatmap and GO terms analysis of Phago-hepa-Muse cell clusters (Phago-hepa-1, -2, -3, -4) and Fetal (Liver cell).

**SI 11) Validation of scRNA seq data by qPCR**

In order to verify the validity of the scRNA seq data of one biological replicate, we select several genes that were upregulated in the scRNA seq data (Phago-Cardio-Muse: *EGLN1*, *SVIL,* *SLC16A7*; Phago-neuro-Muse: *EPHA4*, *ETV1*, *FAM167A*; Phago-hepa-Muse: *AKR1C1*, *SERPINA3*, *CEACAM1*) and those genes were analyzed in qPCR with three biological replicates at 7 days after phagocytosis. These results showed that all genes were up-regulated in Phago-Muse compared to naïve Muse cells with statistical significance (Figure S11).


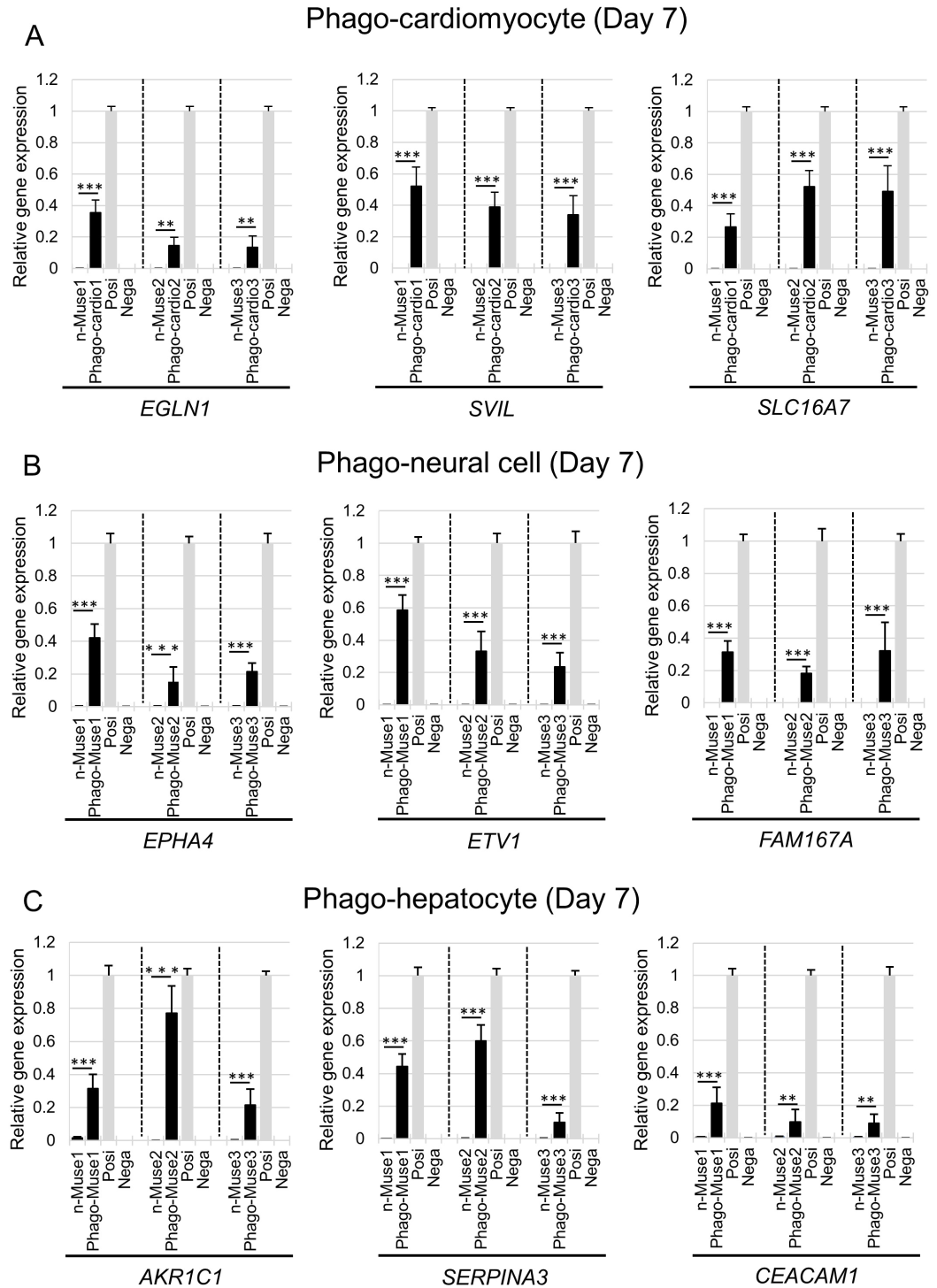


**Figure S11. Three biological replicates of qPCR.**

Lineage-specific marker expression in h-Muse cells 7 days after incubation with apoptotic-cardiomyocyte (A), -neural cell (B), and -hepatocyte (C) (n=3 for each sample).

**SI 12) Next-generation sequencing** **(NGS) of h-MSCs and r-NSCs after incubating with mouse/human-apoptotic cell fragments.**

To confirm global gene expression after DDC phagocytosis, NGS was performed on h-MSCs (naïve h-MSCs), as well as on h-MSCs incubated with m-adipocyte DDCs (Phago-MSCs), or on h-MSCs incubated with annexin V-treated m-adipocyte DDCs (Phago-MSCs + AnnexinV) after 1 week. More than 90 million reads were obtained from each replicate, unmapped reads were excluded, and the remaining reads were mapped to 28,695 genes. Gene expression in each sample was calculated individually, and then gene expression was analyzed. The DEGs results between Naïve-MSCs, Phago-MSCs, and Phago-MSCs + AnnexinV are shown in Figure S12A. The expression of mesenchymal markers was decreased and that of adipogenesis markers was increased in Phago-MSCs compared with naïve MSCs (SI12-Figure S12B). On the other hand, h-MSCs incubated with annexin V-treated m-adipocyte DDCs (Phago-MSCs + AnnexinV) showed a gene expression pattern similar to that of naïve MSCs, suggesting that h-MSC differentiation was inhibited by pretreatment of DDCs with annexin-V (SI 12-Figure S12B). The GO terminology analysis of naïve and phago-MSCs revealed GO terms associated with adipogenic differentiation in phago-MSCs (SI 12-Figure S12C).

Similarly, in r-NSCs, NGS analysis was performed in control r-NSCs (naïve r-NSCs), as well as in r-NSCs incubated with h-neural cell DDCs (Phago-NSCs), or in r-NSCs incubated with annexin V-treated h-neural cell DDCs (Phago-NSCs + AnnexinV) after 1 week. The results of DEGs between Naïve-NSCs, Phago-NSCs, and Phago-NSCs + AnnexinV are shown in Figure S12A. More than 70 million reads were obtained from each replicate, unmapped reads were excluded, and the remaining reads were mapped to 20,333 genes. The DEGs results between Naïve-NSCs, Phago-NSCs, and Phago-NSCs + AnnexinV are shown in Figure S12D. The expression of neurogenesis markers was increased in Phago-NSCs compared with those in naïve NSCs (SI 12-Figure S12E). Incubation of r-NSCs with annexin V-treated h-neural cell DDCs (Phago-NSCs + AnnexinV) showed a gene expression trend similar to that of naïve NSCs (SI12-Figure S12E).


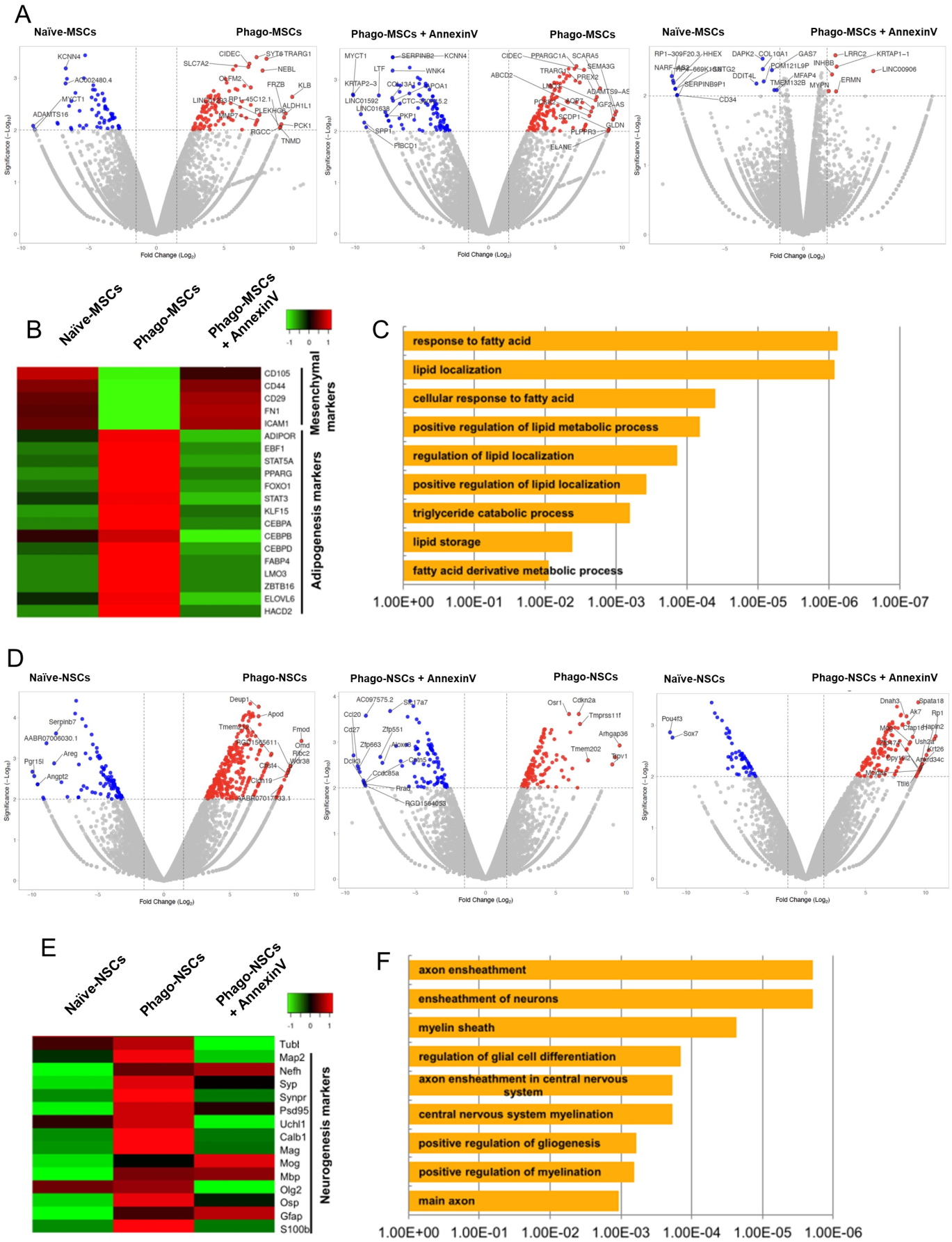


**Figure SI 12) NGS data analysis of h-MSCs and r-NSCs after incubating with mouse/human-apoptotic fragments.** (A) Volcano plot of NGS transcriptome data displaying the difference in the gene expression pattern between the naïve-ｈMSCs, Phago-MSCs, and Phago-MSCs + AnnexinV. The genes that passed the p-value threshold (Log10 1/p-value >2.0) and log fold-change (Log2 fold-change >+1 or <-1 are colored red (upregulated) or blue (downregulated). (B) Heatmaps for mesenchymal stem cell markers and adipogenesis markers between the naïve-MSCs, Phago-MSCs, and Phago-MSCs + AnnexinV. (C) GO term of DEGs between Naïve-MSCs and Phago-MSCs. (D) Volcano plot of NGS transcriptome data displaying the difference in the gene expression pattern between the naïve-ｒNSCs, Phago-NSCs, and Phago-NSCs + AnnexinV. The genes that passed the p-value threshold (Log10 1/p-value >2.0) and log fold-change (Log2 fold-change >+1 or <-1 are colored red (upregulated) or blue (downregulated). (E) Heatmaps for neurogenesis markers between the naïve-rNSCs, Phago-NSCs, and Phago-NSCs + AnnexinV. (F) GO term of DEGs between Naïve-NSCs and Phago-NSCs.

**SI 13) Effect of phagocytosis suppression on differentiation.**

Effect of annexin V on h-Muse cell survival was evaluated (A). Effect of phagocytosis suppression by annexin V on the lineage-specific differentiation in h-MSCs (B) and r-NSCs (C). (B) Apoptotic m-adipocyte DDCs were treated with annexin V and incubated with h-MSCs. (C) Apoptotic h-neuronal cell DDCs were treated with annexin V and incubated with r-NSCs. (D-E) Western blot of each phagocytosis receptor in h-MSCs (D) and r-NSCs (E) in the naïve state and D2 after incubation with m-adipocyte- and h-neuronal cell-DDCs, respectively. Beta-actin (ACTB) is common to all the blots. The signal in the naïve cells is defined as 1. (F) CD36, ITGB3, CD91/LRP-1, and RAGE were each suppressed by small interference RNA (siRNA) in h-Muse cells, which were then exposed to m-cardiomyocyte DDCs. qPCR and Western blotting confirmed the downregulated expression of each receptor type. On the basis of an on-target effect, significant decreases of both human-*NKX2.5* and -*GATA-4* (p<0.05) in CD36 and ITGB3, and significant decreases of either -*NKX2.5* or -*GATA-4* (p<0.05) in CD91/LRP-1 and RAGE were recognized at D7.


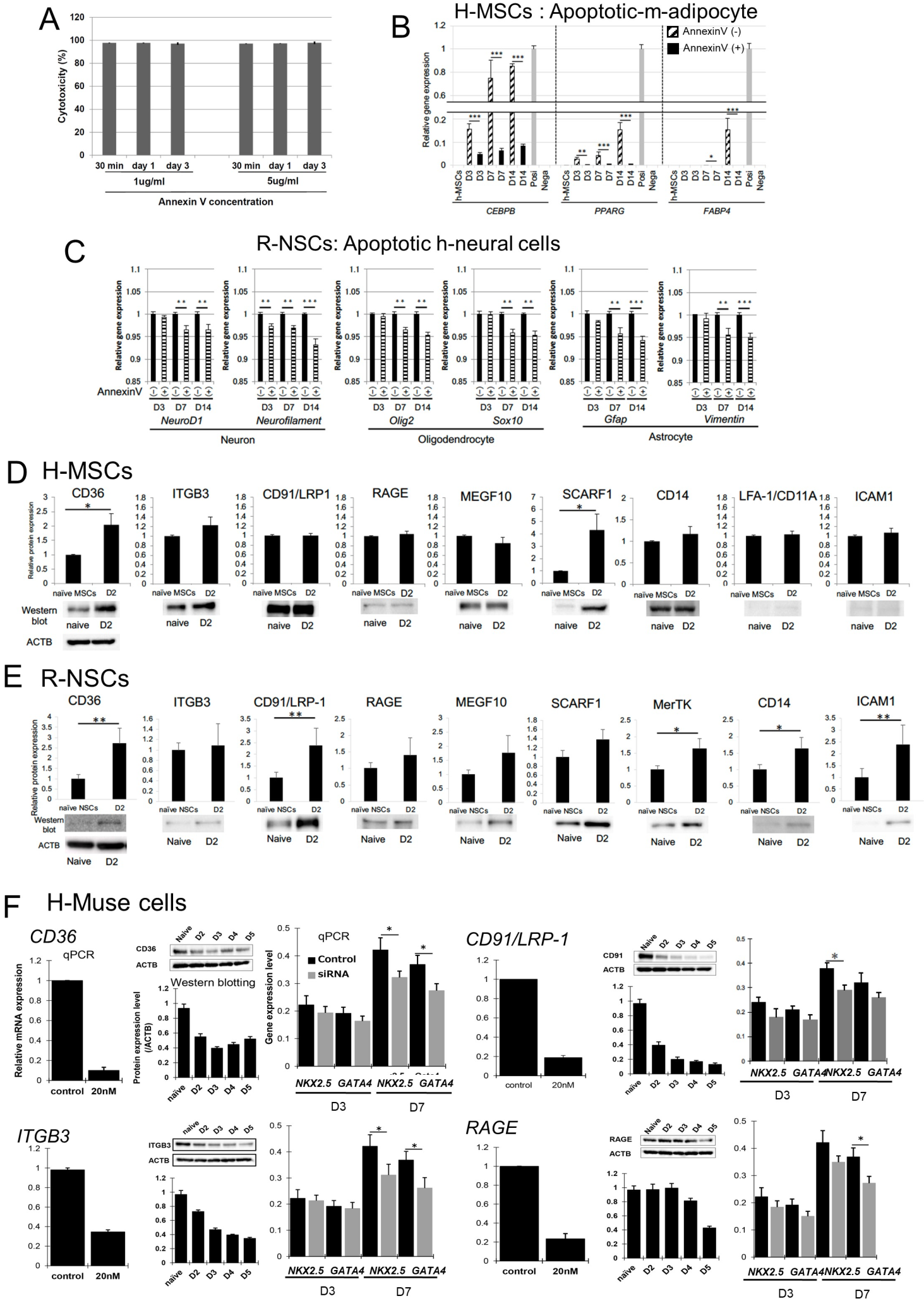


**Figure S13. Effect of phagocytosis suppression on differentiation.** (A) The h-Muse cells were incubated in the presence of either 1 μg/ml or 5 μg/ml annexin V and the resulting cytotoxicity was measured at 30 min, 1 day, or 3 days using a Cytotoxicity LDH Assay Kit (Dojin Chemical, Japan). Annexin V did not induce cytotoxicity at any time-point at either concentration (mean ± SE). (B-C) Effect of phagocytosis suppression by annexin V and lineage-specific differentiation in h-MSCs (B) and r-NSCs (C). (D-E) Western blot of each phagocytosis receptor in h-MSCs (D) and r-NSCs (E). Beta-actin (ACTB) is common to all the blots. The signal in the naïve is set as 1. (F) siRNA for each phagocytosis receptor was introduced into h-Muse cells and then incubated with m-cardiomyocyte-DDCs. qPCR and Western blotting confirmed the downregulated expression of each receptor type. The reduced expression of receptors was confirmed by qPCR and Western blotting. Human-cardiac markers were examined by qPCR (mean ± SEM). *: p<0.05, **:p<0.01, ***:p<0.001.

**SI 14) The in vivo sham group data.**

In a mouse model of focal ischemic stroke induced by local injection of endothelin-1 (ET-1) and a mouse model of bilateral common carotid artery occlusion (BCCAO), we generated sham groups that received either no injection of *NEUROD1*p-CFP-mCherry-labeled h-Muse cells or GFP-labeled h-Muse cells, respectively.

The ET-1-injected C57BL/6-Tg (CAG-EGFP) mouse focal ischemic stroke model was used to detect *NEUROD1*p-CFP-mCherry signal by time-lapse imaging (Figure 6I, Movie 7). In the sham group at 1 day after ET-1 injection, no autofluorescence or artifact in the blue-color code (background for *NEUROD1*p-CFP) or red-color code (background for mCherry) was observed (SI14-Figure S14A).

The BCCAO mouse model was used to examine the effect of siRNA-induced phagocytosis receptor inhibition on GFP-h-Muse cell differentiation in vivo (Figure 6J-N). At 2 days after ischemia induction, the BCCAO mouse model sham group was perfused and brain sections were stained with 2,3,5-triphenyltetrazolium chloride to confirm the ischemic area (SI14-Figure S14B). Then, cryosections were cut and observed under a confocal laser microscope. Autofluorescence or artifact in the green-color code (background for GFP-h-Muse cells) was not observed in the sham group (SI14-Figure S14B, 14C).


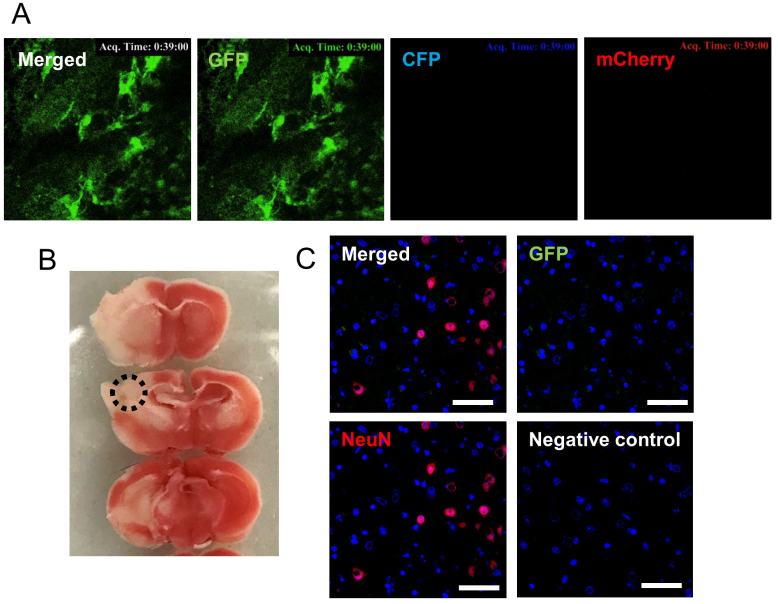


**Figure S14. The sham group data in vivo.** (A) ET-1-injected C57BL/6-Tg (CAG-EGFP) mouse focal ischemic stroke model without topical injection of *NEUROD1*p-CFP-mCherry-h-Muse cells 1 day after ET-1 injection. Multiphoton laser scanning microscopy images of the infarct area. No autofluorescence or artifact in the blue-color code (background for *NEUROD1*p-CFP) or red-color code (background for mCherry) was observed. (B) 2,3,5-Triphenyltetrazolium chloride staining of the BCCAO model sham group at 2 days after stroke, confirming the ischemic area. (C) NeuN(+) host cells in the infarcted area enclosed by the black dotted circle in Figure S13B. Autofluorescence or artifact in the green-color code, suggesting GFP(+)-h-Muse cells, was not observed. Bar = 100 μm.

**SI 15) Effect of annexin V on phagocytosis activity of h-Muse cells.**

(A) M-HL-1 cells expressing GATA-4-mCherry fusion protein (GATA-4-m-HL-1) was confirmed. (B-E) After annexin V treatment, DDCs derived from GATA-4-m-HL-1 were not successfully phagocytosed by h-Muse cells.
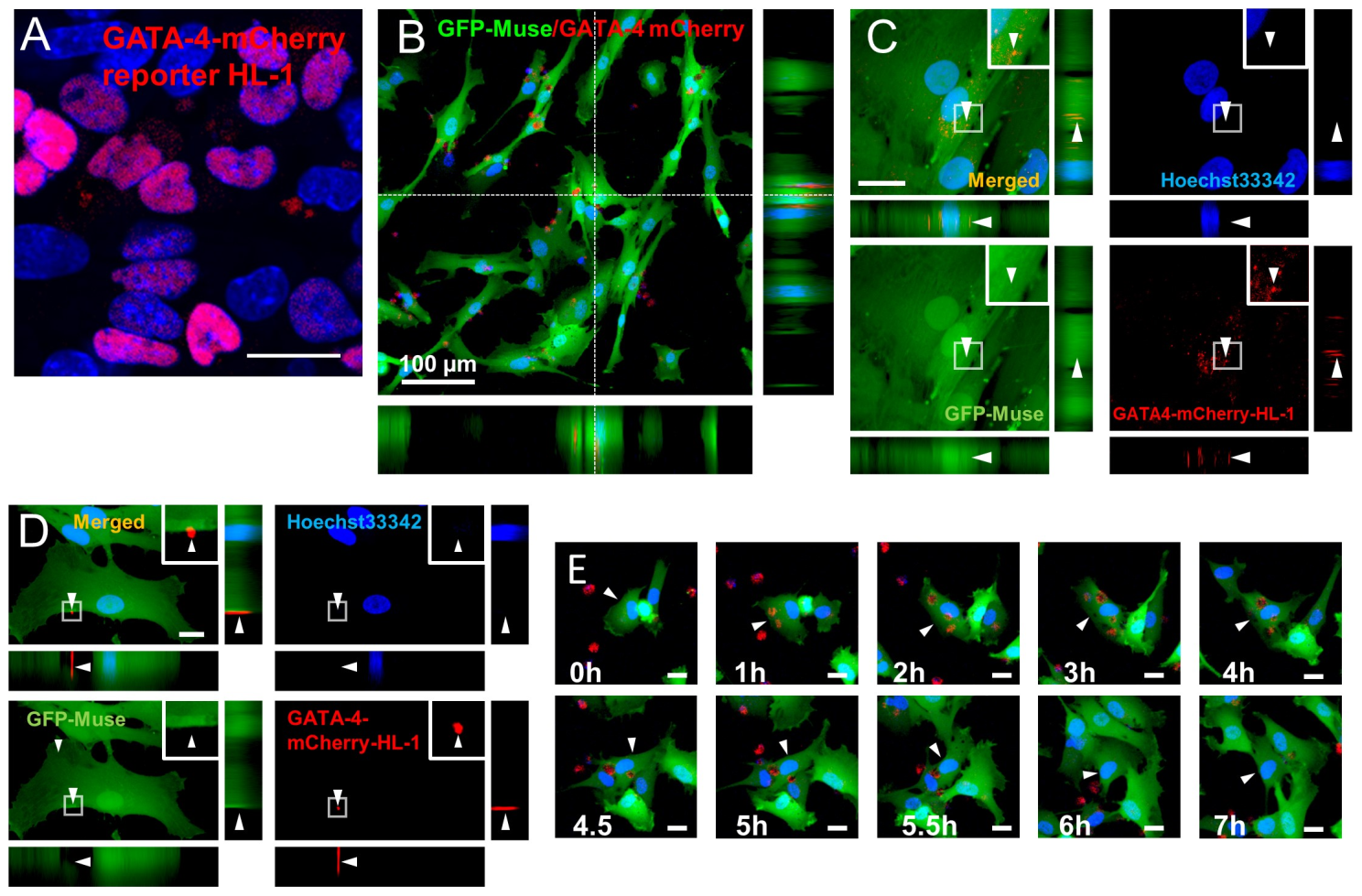


**Figure S15. Phagocytosis of annexin V-treated apoptotic GATA-4-m-HL-1 fragments.**

(A) GATA-4-mCherry fusion protein expression in the nucleus of GATA-4-m-HL-1. (B-E) Laser confocal microscopy images of GFP-h- Muse cells incubated with apoptotic-GATA-4 -m-HL-1-DDCs. (B) 10 h after incubation. GATA-4-mCherry fusion protein was taken up in the GFP-h-Muse cell cytoplasm. (C) 9 h after incubation. Phagocytosed GATA-4-mCherry fusion protein located in the GFP-h-Muse cell cytoplasm. (D) Incubation of GFP-h-Muse cells and annexin V-treated apoptotic-GATA-4-m-HL-1-DDCs at 24 h. The GATA4-mCherry signal was not incorporated into the GFP-h-Muse cell cytoplasm but was incorporated on the cell surface. (E) Incubation with annexin V-treated apoptotic GATA-4m-HL-1-DDCs. GATA-4-mCherry was at once phagocytosed and released to the extracellular space. Bars; A, C, D, E = 50 μm, B = 100 μm.

**SI 16) Comparison of ChIP seq data between Phago-Muse cells and previously reported iPS cell-derived cardiomyocytes.**

We compared the GATA4-ChIP seq data of Phago-Muse cells 16 hrs after incubating with DDCs with that of iPS cell-derived cardiomyocytes at day 6 (GSE159411; Cardiomyocyte) and day 32 (GSE85631; Cardiomyocyte) after differentiation induction, respectively, in order to examine an overlap of GATA4 binding sites [17, 18]. Thirty overlaps were found when compared with day 32 mature iPS cell-derived cardiomyocytes (SI 16-Figure S16A). When compared with day 6 iPS cell-derived cardiomyocyte, the overlap of GATA4 binding sites increased to 157 (SI 16-Figure S16B).


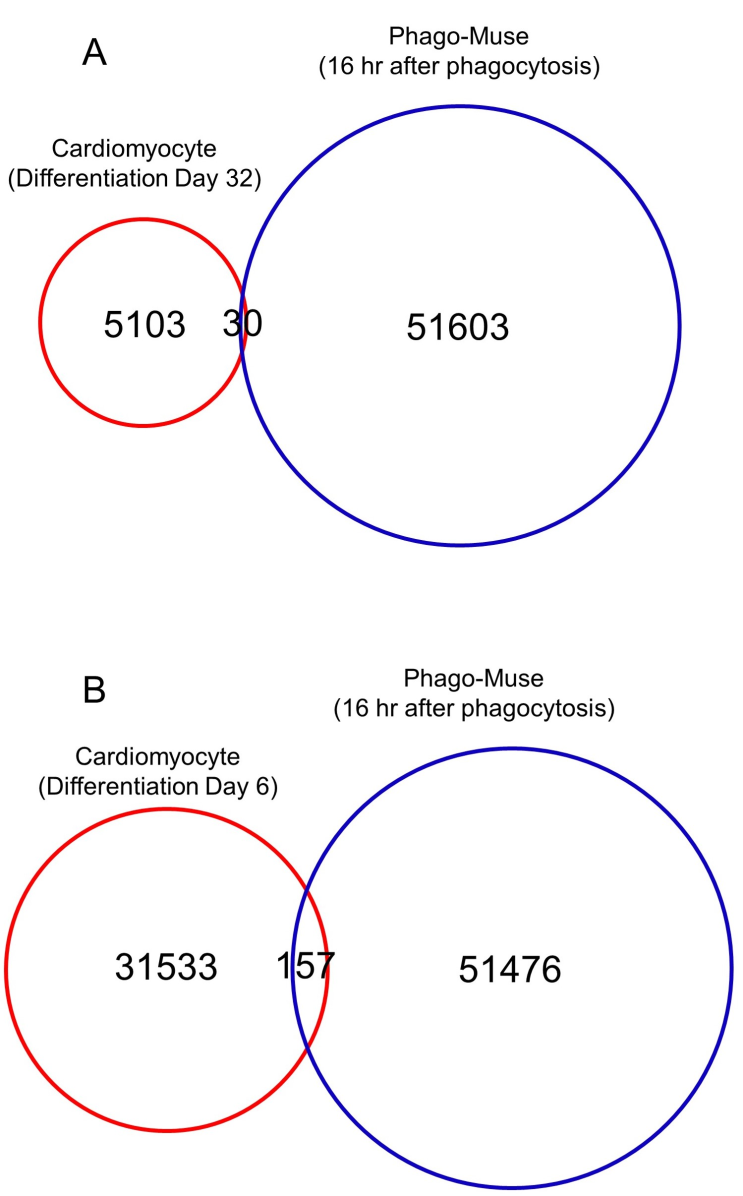


**Fig. S16. Overlap of GATA4 binding sites.** (A) Venn diagram comparing Phago-Muse with iPS cell-derived cardiomyocytes at day 32 after induction (GSE85631). (B) Venn diagram comparing Phago-Muse with iPS cell-derived cardiomyocytes at day 6 after induction (GSE159411).

**SI 17) Transmission electron microscopy.**

GFP-h-Muse cells were incubated for 12–24 h with apoptotic-mCherry-m-Hepa-1-6 DDCs on a cover glass with a grid, allowing Muse cells to phagocytose DDCs. The cover glass was then fixed with 2.5% glutaraldehyde + 4% PFA in 0.1 M phosphate buffer and incubated with DAPI for obtaining images under a laser confocal microscope. The sample was subjected to post fixation with 1% osmium tetroxide in 0.1 M phosphate buffer, dehydrated in ethanol and propylene oxide, and embedded in epoxy resin; the cover glass was removed, and after cutting the sample into ultrathin sections, it was observed under an electron microscope.


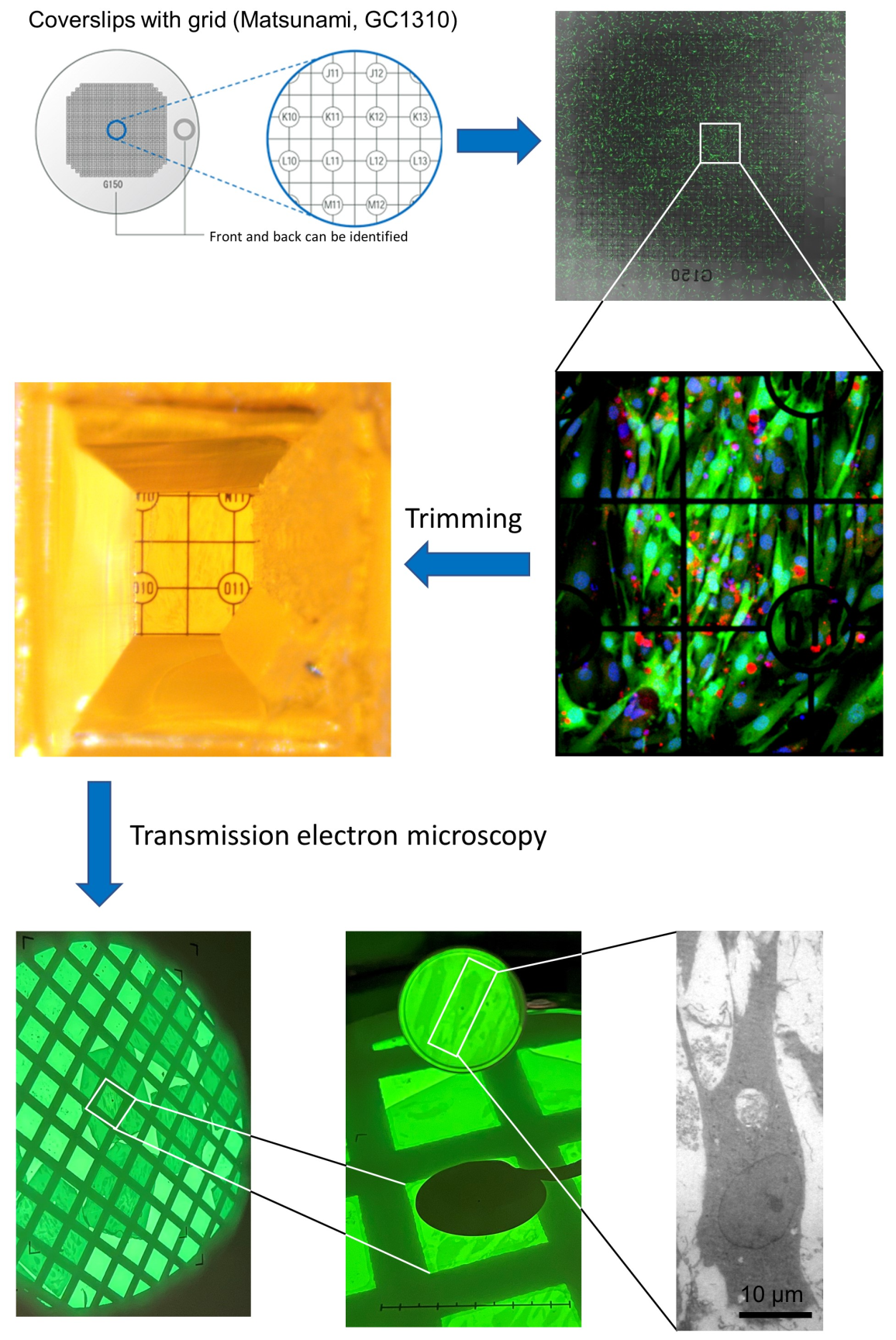


**Figure S17. Preparation of transmission electron microscopy sample.**

The sample preparation process, namely incubation of h-Muse cells with DDCs on a cover slide containing a grid and trimming the epon-embedded sample, for observation under transmission electron microscope is described.

**SI 18) Phagocytotic activity of iPS cells.**

Human fibroblast-derived iPS cells [19] labeled with PKH67 were incubated with apoptotic mCherry-m-Hepa1 DDCs. The phagocytosis rate was 4.9±1.5% at 5 h and 5.3±0.9% at 24 h, respectively.


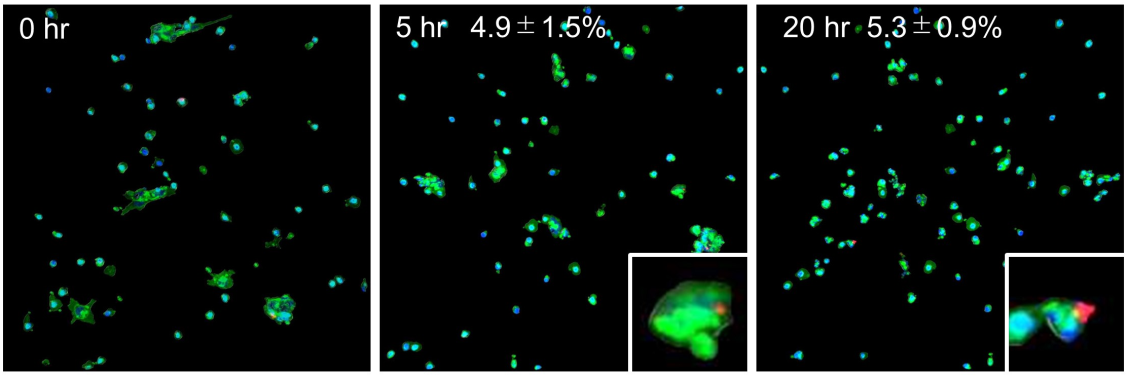


**Figure S18. Images of h-iPS cells incubated with apoptotic mCherry-m-Hepa1 DDCs.**

**Figure S19. Full scans of uncropped Western blots in Figure 6.**

**
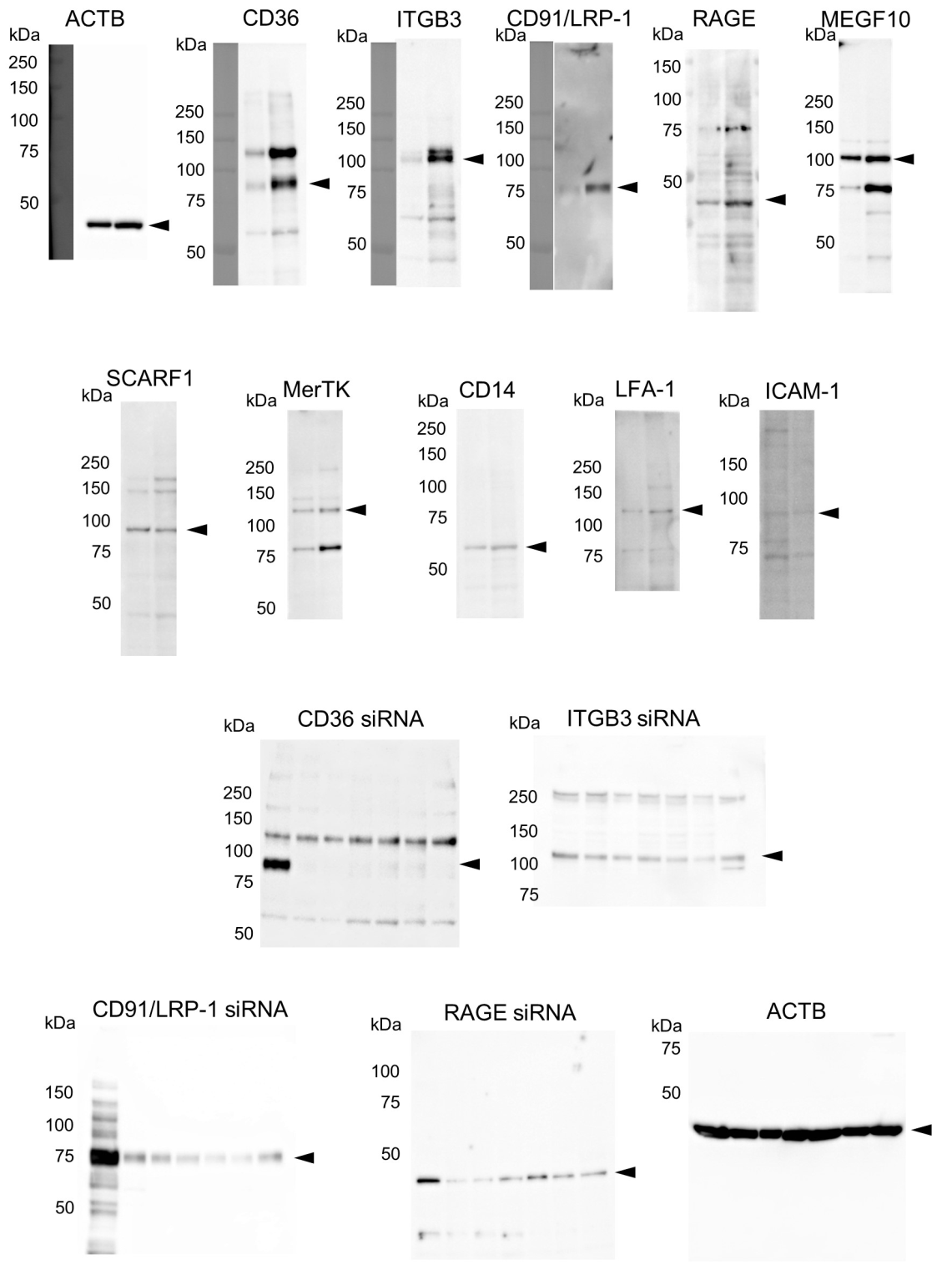
**

**Figure S20. Full scans of uncropped Western blots in Figure S12.**

**
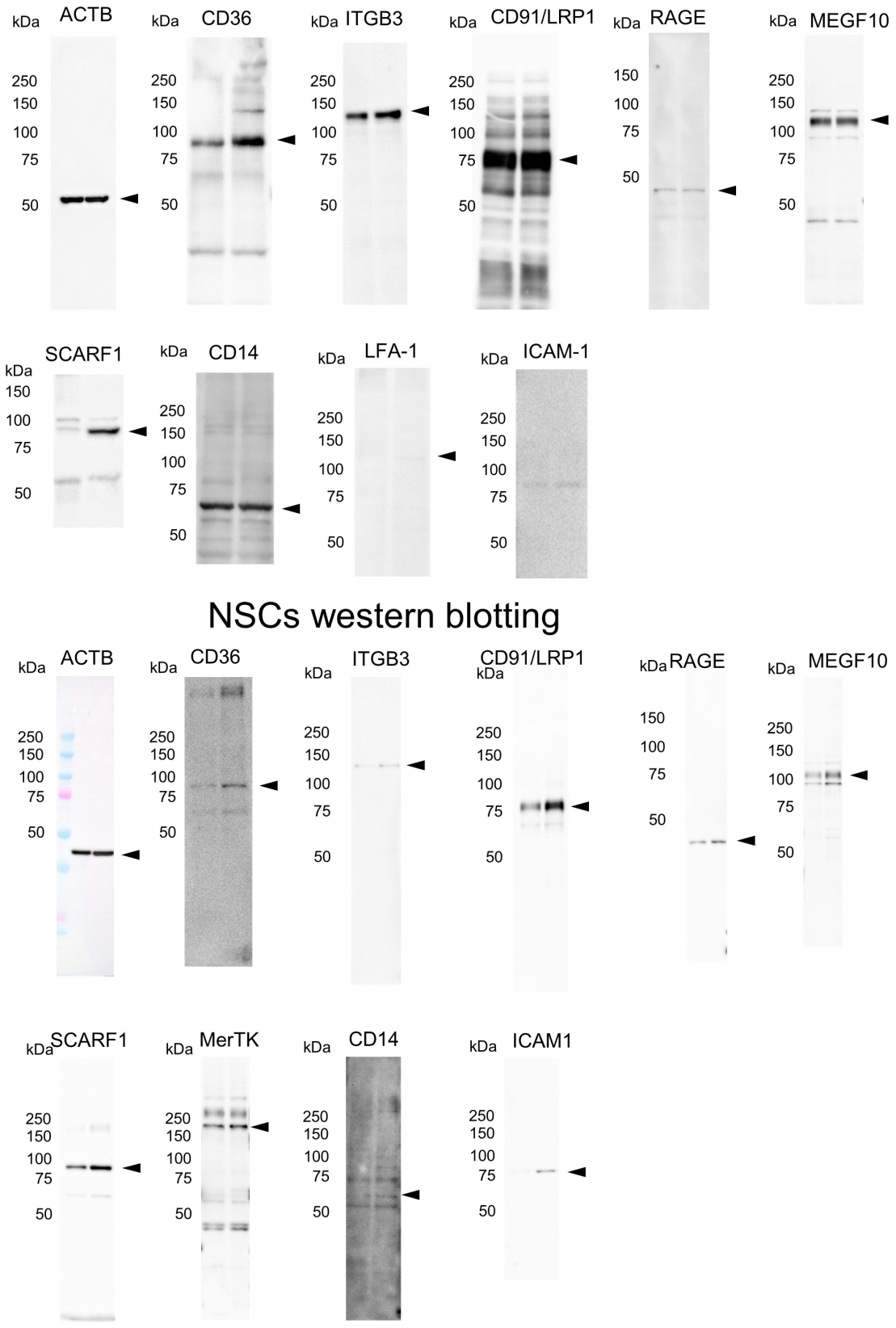
**

**Figure S21. Full scans of uncropped Western blots in Figure S12.**

**
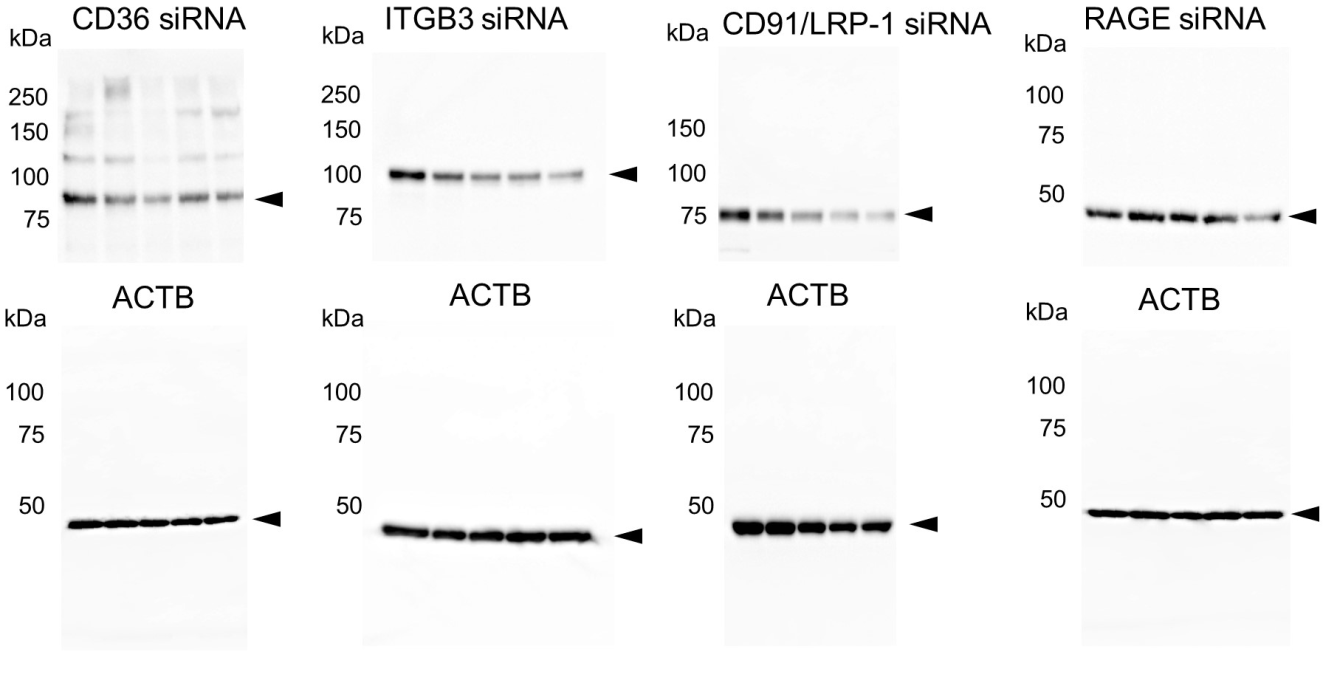
**

**Figure S22. Full scans of uncropped Western blots in Figure 7.**

**
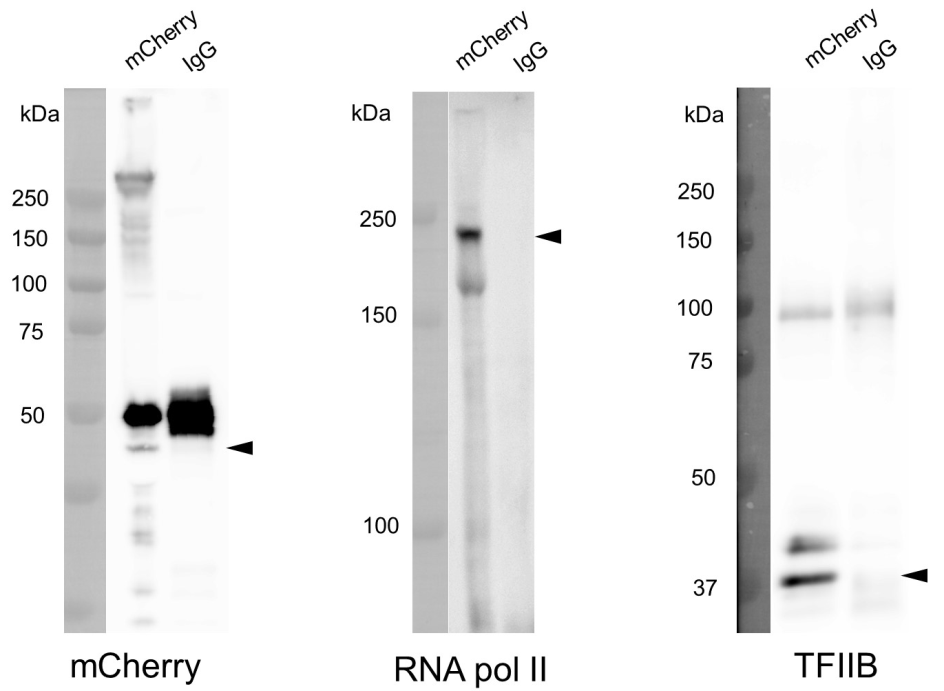
**

**References**

1. M. Amin, Y. Kushida, S. Wakao, M. Kitada, K. Tatsumi, M. Dezawa, Cardiotrophic Growth Factor-Driven Induction of Human Muse Cells Into Cardiomyocyte-Like Phenotype. *Cell Transplantation* **27**, 285-298 (2018).
2. A. Raso, E. Dirkx, Cardiac regenerative medicine: At the crossroad of microRNA function and biotechnology. *Noncoding RNA Res* **2**, 27-37 (2017).
3. J. S. Choi, H. I. Yoon, K. S. Lee, Y. C. Choi, S. H. Yang, I. S. Kim, Y. W. Cho, Exosomes from differentiating human skeletal muscle cells trigger myogenesis of stem cells and provide biochemical cues for skeletal muscle regeneration. *J Control Release* **222**, 107-115 (2016).
4. Q. Xu, Y. Cui, J. Luan, X. Zhou, H. Li, J. Han, Exosomes from C2C12 myoblasts enhance osteogenic differentiation of MC3T3-E1 pre-osteoblasts by delivering miR-27a-3p. *Biochem Biophys Res Commun* **498**, 32-37 (2018).
5. M. Crisan, S. Yap, L. Casteilla, C. W. Chen, M. Corselli, T. S. Park, G. Andriolo, B. Sun, B. Zheng, L. Zhang, C. Norotte, P. N. Teng, J. Traas, R. Schugar, B. M. Deasy, S. Badylak, H. J. Buhring, J. P. Giacobino, L. Lazzari, J. Huard, B. Peault, A perivascular origin for mesenchymal stem cells in multiple human organs. *Cell Stem Cell* **3**, 301-313 (2008).
6. E. Matuzelski, J. Bunt, D. Harkins, J. W. C. Lim, R. M. Gronostajski, L. J. Richards, L. Harris, M. Piper, Transcriptional regulation of Nfix by NFIB drives astrocytic maturation within the developing spinal cord. *Dev Biol* **432**, 286-297 (2017).
7. S. A. Russell, G. J. Bashaw, Axon guidance pathways and the control of gene expression. *Dev Dyn* **247**, 571-580 (2018).
8. P. Palmquist-Gomes, J. A. Guadix, J. M. Perez-Pomares, Avian embryonic coronary arterio-venous patterning involves the contribution of different endothelial and endocardial cell populations. *Dev Dyn* **247**, 686-698 (2018).
9. Y. Kondo, I. D. Duncan, Myelin repair by transplantation of myelin-forming cells in globoid cell leukodystrophy. *J Neurosci Res* **94**, 1195-1202 (2016).
10. O. Jahn, S. Tenzer, H. B. Werner, Myelin proteomics: molecular anatomy of an insulating sheath. *Mol Neurobiol* **40**, 55-72 (2009).
11. G. M. Mager, R. M. Ward, R. Srinivasan, S. W. Jang, L. Wrabetz, J. Svaren, Active gene repression by the Egr2.NAB complex during peripheral nerve myelination. *J Biol Chem* **283**, 18187-18197 (2008).
12. P. Iacopetti, G. Barsacchi, F. Tirone, L. Maffei, F. Cremisi, Developmental expression of PC3 gene is correlated with neuronal cell birthday. *Mech Dev* **47**, 127-137 (1994).
13. J. S. Oh, P. Manzerra, M. B. Kennedy, Regulation of the neuron-specific Ras GTPase-activating protein, synGAP, by Ca2+/calmodulin-dependent protein kinase II. *J Biol Chem* **279**, 17980-17988 (2004).
14. Y. Sun, H. Zhang, L. Wang, J. Li, H. Jin, Z. Wang, S. Tian, L. Qi, X. Liu, Loss of the basic helix-loop-helix transcription factor Bhlhe41 induces cell death and impairs neurite outgrowth in Neuro2a cells. *Mol Cell Biochem* **450**, 167-174 (2019).
15. A. Kimura, T. Matsuda, A. Sakai, N. Murao, K. Nakashima, HMGB2 expression is associated with transition from a quiescent to an activated state of adult neural stem cells. *Dev Dyn* **247**, 229-238 (2018).
16. M. Uhlen, L. Fagerberg, B. M. Hallstrom, C. Lindskog, P. Oksvold, A. Mardinoglu, A. Sivertsson, C. Kampf, E. Sjostedt, A. Asplund, I. Olsson, K. Edlund, E. Lundberg, S. Navani, C. A. Szigyarto, J. Odeberg, D. Djureinovic, J. O. Takanen, S. Hober, T. Alm, P. H. Edqvist, H. Berling, H. Tegel, J. Mulder, J. Rockberg, P. Nilsson, J. M. Schwenk, M. Hamsten, K. von Feilitzen, M. Forsberg, L. Persson, F. Johansson, M. Zwahlen, G. von Heijne, J. Nielsen, F. Ponten, Proteomics. Tissue-based map of the human proteome. *Science* **347**, 1260419 (2015).
17. B. Gonzalez-Teran, M. Pittman, F. Felix, R. Thomas, D. Richmond-Buccola, R. Huttenhain, K. Choudhary, E. Moroni, M. W. Costa, Y. Huang, A. Padmanabhan, M. Alexanian, C. Y. Lee, B. E. J. Maven, K. Samse-Knapp, S. U. Morton, M. McGregor, C. A. Gifford, J. G. Seidman, C. E. Seidman, B. D. Gelb, G. Colombo, B. R. Conklin, B. L. Black, B. G. Bruneau, N. J. Krogan, K. S. Pollard, D. Srivastava, Transcription factor protein interactomes reveal genetic determinants in heart disease. *Cell* **185**, 794-814 e730 (2022).
18. Y. S. Ang, R. N. Rivas, A. J. S. Ribeiro, R. Srivas, J. Rivera, N. R. Stone, K. Pratt, T. M. A. Mohamed, J. D. Fu, C. I. Spencer, N. D. Tippens, M. Li, A. Narasimha, E. Radzinsky, A. J. Moon-Grady, H. Yu, B. L. Pruitt, M. P. Snyder, D. Srivastava, Disease Model of GATA4 Mutation Reveals Transcription Factor Cooperativity in Human Cardiogenesis. *Cell* **167**, 1734-1749 e1722 (2016).
19. S. Wakao, M. Kitada, Y. Kuroda, T. Shigemoto, D. Matsuse, H. Akashi, Y. Tanimura, K. Tsuchiyama, T. Kikuchi, M. Goda, T. Nakahata, Y. Fujiyoshi, M. Dezawa, Multilineage-differentiating stress-enduring (Muse) cells are a primary source of induced pluripotent stem cells in human fibroblasts. *Proc Natl Acad Sci U S A* **108**, 9875-9880 (2011).
